# Further *N*-Frame networking dynamics of conscious observer-self agents via a functional contextual interface: predictive coding, double-slit quantum mechanical experiment, and decision-making fallacy modeling as applied to the measurement problem in humans and AI

**DOI:** 10.3389/fncom.2025.1551960

**Published:** 2025-04-01

**Authors:** Darren J. Edwards

**Affiliations:** Department of Public Health, Swansea University, Swansea, United Kingdom

**Keywords:** predictive coding, functional contextualism, *N*-Frame, quantum mechanics, artificial intelligence

## Abstract

Artificial intelligence (AI) has made some remarkable advances in recent years, particularly within the area of large language models (LLMs) that produce human-like conversational abilities via utilizing transformer-based architecture. These advancements have sparked growing calls to develop tests not only for intelligence but also for consciousness. However, existing benchmarks assess reasoning abilities across various domains but fail to directly address consciousness. To bridge this gap, this paper introduces the functional contextual *N*-Frame model, a novel framework integrating predictive coding, quantum Bayesian (QBism), and evolutionary dynamics. This comprehensive model explicates how conscious observers, whether human or artificial, should update beliefs and interact within a quantum cognitive system. It provides a dynamic account of belief evolution through the interplay of internal observer states and external stimuli. By modeling decision-making fallacies such as the conjunction fallacy and conscious intent collapse experiments within this quantum probabilistic framework, the *N*-Frame model establishes structural and functional equivalence between cognitive processes identified within these experiments and traditional quantum mechanics (QM). It is hypothesized that consciousness serves as an active participant in wavefunction collapse (or actualization of the physical definite states we see), bridging quantum potentiality and classical outcomes via internal observer states and contextual interactions via a self-referential loop. This framework formalizes decision-making processes within a Hilbert space, mapping cognitive states to quantum operators and contextual dependencies, and demonstrates structural and functional equivalence between cognitive and quantum systems in order to address the measurement problem. Furthermore, the model extends to testable predictions about AI consciousness by specifying informational boundaries, contextual parameters, and a conscious-time dimension derived from Anti-de Sitter/Conformal Field Theory correspondence (AdS/CFT). This paper theorizes that human cognitive biases reflect adaptive, evolutionarily stable strategies that optimize predictive accuracy (i.e., evolved quantum heuristic strategies rather than errors relative to classical rationality) under uncertainty within a quantum framework, challenging the classical interpretation of irrationality. The *N*-Frame model offers a unified account of consciousness, decision-making, behavior, and quantum mechanics, incorporating the idea of finding truth without proof (thus overcoming Gödelian uncertainty), insights from quantum probability theory (such as the Linda cognitive bias findings), and the possibility that consciousness can cause waveform collapse (or perturbation) accounting for the measurement problem. It proposes a process for conscious time and branching worldlines to explain subjective experiences of time flow and conscious free will. These theoretical advancements provide a foundation for interdisciplinary exploration into consciousness, cognition, and quantum systems, offering a path toward developing tests for AI consciousness and addressing the limitations of classical computation in representing conscious agency.

## 1 Introduction

Artificial intelligence (AI) has made some remarkable advances in recent years, particularly within the area of large language models (LLMs) that produce human-like conversational abilities via utilizing transformer-based architecture starting with a seminal paper from Google “*Attention is all you need*” (Vaswani et al., 2017). A step-by-step guide through this transformer architecture is given elsewhere (Edwards, 2024; Radford et al., 2018).

With this monumental rise in AI intelligence, there have been increasing calls for developing tests for measuring intelligence across many domains, general reasoning, and whether this could lead to consciousness (Bołtuć, 2020; Chalmers, 2023; Chollet, 2019; Lee, 2020). A test for AI consciousness has been extremely elusive, and there are currently no widely accepted standardized tests for determining whether an AI is genuinely conscious.

Many of the AI benchmarks test math or reasoning skills, such as “*Needle in the Haystack*,” “*General language understanding evaluation (GLUE)*,” “*ARC-AGI*,” “*AIME competition math*,” and “*GPQA Diamond* (science questions).” These are not consciousness measures but rather focus on evaluating an AI system’s reasoning, problem-solving, or linguistic competence in areas such as math problems, understanding language, or reasoning about facts, rather than whether the model possesses any conscious experience or self-awareness. Furthermore, Turing (Turing, 1950) suggested the “*imitation game*” (the Turing test), to test for human-like intelligence in AI, specifically focused on AI’s ability to produce human-level language (i.e., imitate a human conversational ability) but again this is not a test of conscious experience (qualia, e.g., color, taste, or the feeling of pain) that AI may have.

Chalmers (Chalmers, 1995, 1997) refers to the easy problem of consciousness as neural correlates of consciousness (NCCs) and the hard problem as the specific process of consciousness emerging from neurons (utilizing a physicalist interpretation of reality). To date, progress has only been made on the easy problem of consciousness i.e., the neural correlates of consciousness such as neuron clusters that are broadly required for conscious experience (De Graaf et al., 2012; Koch et al., 2016; Noë and Thompson, 2004; Rees et al., 2002) such as V5 of the visual pathway responsible for motion detection (Tootell et al., 1995) and other areas of the visual neuron pathway that project onto the frontal cortex for conscious representation of visual perception (Crick and Koch, 1995). However, this physicalist interpretation of consciousness has been shown to be severely limited as it leads to a long unresolved mind-body problem (Armstrong, 2018; Bunge, 2014; Feyerabend, 1963; Ludwig, 2003). This mind-body problem is a problem about how mental states such as conscious states relate to physical states originally proposed by René Descartes in 1641 (Descartes, 2013). The mind-body problem highlights the difficulty of explaining consciousness as emerging from neurons, and after decades of years of research, there has been little progress in this area as we are no closer to solving this hard problem. Neuroscience theories that have tried to solve this mind-body hard problem such as integrated information theory (IIT) (Merker et al., 2022; Tononi, 2015; Tononi et al., 2016) that rely on a physicalist model cannot explain how a single phenomenological conscious experience (such as the taste of chocolate, or the feeling of compassionate love) casually arises. This physicalist model is potentially severely limited and entirely the wrong ontological framework in answering the question of whether AI could be conscious.

Nobel Prize-winning physicist Roger Penrose argues that human consciousness cannot be fully explained by classical computational processes (Penrose, 1991, 1994). Drawing on Gödel’s incompleteness theorems (Gödel, 1931), he suggests that human mathematicians can discern certain truths that formal algorithms cannot prove, indicating that human consciousness involves non-algorithmic understanding. In collaboration with anesthesiologist Stuart Hameroff, Penrose developed the Orch OR (Orchestrated Objective Reduction) model (Hameroff and Penrose, 2014; Hameroff and Penrose, 2017; Penrose and Hameroff, 2011), which proposes that consciousness arises from quantum state reductions (objective wavefunction collapses) occurring within the brain’s microtubular structures. They suggest via their Orch OR model, that the key idea of certain aspects of conscious thought, particularly the kind of intuitive or insightful understanding that allows us to recognize truths not formally provable by algorithmic processes, arise from quantum-level phenomena rather than classical, step-by-step computation.

In the Orch OR model (Hameroff and Penrose, 2014; Hameroff and Penrose, 2017; Penrose and Hameroff, 2011), consciousness is thought to arise at moments when quantum superpositions of quantum mechanics (QM) within the brain’s microtubules reach a critical threshold and undergo “objective reduction” (OR). This OR event is not thought to be a calculation following a strict rule, instead, it is assumed to be a fundamental, non-computational process inherent to nature. The idea is that the wavefunction collapse, governed by quantum principles, yields outcomes in a way that is not equivalent to running through a step-by-step algorithm. Instead, it selects a particular reality from a set of quantum possibilities. Because these quantum reductions are not simply like flipping bits or executing classical algorithms, they are thought to introduce a kind of shortcut or non-computable step into the thought process. In other words, the conscious mind might access truths or insights in a manner not reproducible by a straightforward computational sequence. This would help explain why human mathematicians and thinkers can discern the validity (truth) of certain statements that formal systems can never prove.

The problem with this suggestion is that it is entirely speculative, and points to some new interpretation of QM that is entirely unspecified. Given our current understanding of QM, it is entirely insufficient to find truth without proof along some waveforms. Quantum computing offers powerful computational capabilities by leveraging principles such as superposition and entanglement. However, there are fundamental limitations imposed by computability theory and mathematical logic, particularly regarding unprovable statements within formal systems. Gödel’s incompleteness theorems reveal that in any sufficiently powerful formal system, there exist true statements that cannot be proven within that system. This raises a critical question: Can quantum computers (QM applied to solving problems as we understand them) determine the truth of such unprovable statements? We can rigorously prove that quantum computers cannot determine the truth of such unprovable statements within a formal system via any algorithm utilizing quantum superposition and coherence alone. This can be shown directly via a proof that utilizes bounded-error quantum polynomial-time (BQP), which is a complexity class that characterizes the set of decision problems solvable by a quantum computer in polynomial time with a low probability of error.

This construction proof of a BQP ⊆ polynomial space (PSPACE) theorem is extended from a result in computational complexity theory, specifically in quantum complexity theory (Bennett et al., 1997). Theorem: Every problem solvable by a quantum polynomial-time algorithm with bounded error is also solvable by a deterministic Turing machine using only polynomial space. Thus, quantum computations do not transcend classical space complexity boundaries. Axiom 1 (BQP definition): BQP is the class of decision problems solvable by a uniform family of polynomial-size quantum circuits (or equivalently, polynomial-time quantum Turing machines) with bounded error. More precisely, a language *L* is in BQP if there exists a polynomial-size quantum circuit family {*C*_*n*_} such that: For inputs *x* of length *n*, if *x* ∈ *L*, then *C_n_* accepts (makes a decision) with probability at least 2/3. If *x* ∉ *L*, then *C_n_* accepts with probability at most 1/3. Axiom 2 (universal gate sets and polynomial-size circuits): There exists a finite universal set of quantum gates from which one can construct a polynomial-size quantum circuit that simulates any polynomial-time quantum computation. Each gate acts on at most a constant number of qubits. Note: This is standard in quantum computation theory. Axiom 3 (acceptance probability gap): For languages in BQP, there is a promised gap in acceptance probabilities: If *x* ∈ *L*, the quantum algorithm’s acceptance probability is at least 2/3. If *x* ∉ *L*, this probability is at most 1/3. This gap of at least 1/3 ensures that an approximation to within 1/6 suffices to distinguish the two cases. Axiom 4 (Basic arithmetic and precision management): Arithmetic operations on integers and rationals (numbers) with polynomially many bits of precision can be performed using polynomial space. This includes addition, multiplication, and comparisons to a given precision. Thus, any computation involving polynomially large rational numbers can be carried out in PSPACE. Axiom 5 (Church-Turing Thesis): All effectively computable functions are exactly those computable by a Turing machine. This establishes the Turing model of computation as the universal framework for defining computability. Axiom 6 (PSPACE definition): PSPACE is the class of decision problems solvable by a deterministic Turing machine using space polynomial in the size of the input. Formally, a language *L* is in PSPACE if there exists a deterministic Turing machine *M* and a polynomial *p* such that for every input *x*, *M* decides whether *x* ∈ *L* in space *O*[*p*(|*x*|)].

Proof by Construction. Step 1 (set up): Let *L* ∈ *BPQ*. By Axiom 1, there is a polynomial-size quantum circuit family {*C*_*n*_} deciding *L*. For an input *x* of length *n*, the circuit *C_n_* has *r*(*n*) qubits and at most *T*(*n*) gates, whereby *r*(*n*) and *T*(*n*) are polynomials in *n*. Each gate is from a universal set (by Axiom 2), and the initial state is |0⟩^⊗*r*(*n*)^. After applying all *T*(*n*) gates, a measurement on designated output qubits determines acceptance. Step 2 (acceptance probability as a function of amplitudes): The final state |ψ_*final*_⟩ is obtained by successively applying the *T*(*n*) unitary gates: |ψ_*final*_⟩ = *U*_*T*(*n*)_*U*_*T*(*n*)−1_*U*_1_|0⟩^⊗*r*(*n*)^. The acceptance probability *p*_*accept*_(*x*) = ||*P*_*accept*_|ψ_*final*_||^2^, where *P*_*accept*_ is the projector onto the accepting subspace. Step 3 (naive simulation and exponential complexity): A naive classical simulation would require tracking 2^*r*(*n*)^ amplitudes, potentially exponential in *r*(*n*). This would exceed polynomial space and thus is not feasible directly. Step 4 (depth-first computation of amplitudes): Instead of storing the entire state, we use a divide-and-conquer approach by breaking down the action of each gate into smaller computations, whereby we compute partial amplitudes incrementally, and use a depth-first strategy to combine these partial results, whilst discarding intermediate data once added to a running total. By doing so, we never store all amplitudes at once. Step 5 (precision and error bound): By Axiom 3, if *x* ∈ *L*, *p*_*accept*_(*x*) ≥ 2/3 and if *x* ∉ *L*, *p*_*accept*_(*x*) ≤ 1/3. Thus, distinguishing these cases requires approximating *p*_*accept*_(*x*) to within 1/6. By Axiom 4, arithmetic on rationals with polynomially many bits of precision can be done in polynomial space. Thus, we can achieve the required precision using only polynomial space. Step 6 (Constructing a PSPACE Algorithm): We now define a deterministic Turing machine *M* that, on input *x* constructs (or simulates constructing) the quantum circuit *C_n_* from Axiom 5 (this is possible within the Church-Turing framework guaranteed by Axiom 1, as constructing and describing the circuit is an effective procedure) using the depth-first strategy (from Step 4) and rational arithmetic (Axiom 4) to approximate *p*_*accept*_(*x*). It compares the computed acceptance probability to a threshold (e.g., 1/2) to decide whether *x* ∈ *L*. Since we use only polynomially many bits of storage at each step, and *T*(*n*) is polynomial, the entire simulation fits within polynomial space. By Axiom 6, if a deterministic Turing machine uses polynomial space, the language it decides is in PSPACE, thus, *L* ∈ PSPACE. Step 7 (conclusion): We have shown that any *L* ∈ BQP can be decided by a polynomial-space deterministic Turing machine. Hence BQP ⊆ PSPACE. Since *L* was arbitrary in BQP and we constructed a PSPACE algorithm for it, we have established the inclusion. This is also established as true via supportive evidence from Bennett et al. (1997).

By proving the theorem BQP ⊆ PSPACE, we establish that every problem that can be efficiently solved by a bounded-error quantum polynomial-time algorithm can also be decided by a deterministic Turing machine using only polynomial space. In other words, we have shown that quantum computations, despite their apparent superiority in certain algorithmic domains, do not escape the classical complexity class PSPACE in terms of space usage. Penrose’s argument, as presented in works such as articulated in his books *The Emperor’s New Mind* and *Shadows of the Mind* (Penrose, 1991, 1994), suggests that human understanding, particularly our ability to grasp truths that might be related to Gödel’s incompleteness theorems, cannot be fully captured by standard computational models (Turing machines) and may require non-classical, possibly quantum, processes. In other words, Penrose posits that the human mind can do something beyond what any standard classical algorithmic process could achieve via some quantum process, however, the BQP ⊆ PSPACE theorem and proof seems to refute this claim.

The theorem BQP ⊆ PSPACE i.e., the statement that every problem efficiently solvable by a quantum computer with bounded error also lies within the classical complexity class PSPACE, has a bearing on Penrose’s claim in the following ways: (1) Computational Model Bounds: BQP ⊆ PSPACE shows that quantum algorithms, at least within the current standard model of quantum computation, do not transcend fundamental classical complexity class boundaries in terms of space usage. This result strongly suggests that quantum computation, despite offering speed-ups for certain problems, is still within the same overarching framework of computability as classical computation. It does not break free from the Turing paradigm in a way that would allow tackling problems that are known to be non-computable or logically independent of a given formal system. (2) Gödel Incompleteness vs. Algorithmic Power: Gödel’s incompleteness theorems state that any sufficiently powerful formal system contains statements that are true but cannot be proven within that system. These “unprovable truths” are not just algorithmically hard, they are fundamentally outside the scope of logical derivation. Even if you had an unbounded classical or quantum computer, it could not algorithmically “solve” Gödel-incomplete statements in the sense of systematically recognizing all truths of arithmetic that escape formal proof. This is not a problem of time or space complexity, it is instead a fundamental limit of formal systems and algorithms. Quantum mechanics, at least in the standard computational framework, does not allow us to surpass the fundamental logical limitations imposed by Gödel. (3) No extra-special quantum leap beyond computability: Penrose’s conjecture is that perhaps that some form of quantum gravity or non-(classical) algorithmic quantum phenomenon underlies human cognition, enabling us to perceive truths not reachable through computation. But standard quantum computing models do not provide a route to such non-computational capabilities. The BQP ⊆ PSPACE theorem underscores that quantum computing, as mathematically formalized today, is still a form of computational process bounded by classical notions of complexity. In conclusion, the proven theorem BQP ⊆ PSPACE does not support Penrose’s argument that quantum mechanics (in the usual quantum computation model) would break the confines of algorithmic reasoning and thus enable solving Gödel-incomplete statements. Instead, it reaffirms that even quantum computation remains within the standard computational paradigm and does not offer a magical back door around Gödel’s incompleteness.

This conclusion can be even more precisely articulated in the following theorem and proof called the Quantum Gödel Undecidability Theorem. Axiom 1 (Gödel’s First Incompleteness Theorem): In any consistent, effectively axiomatizable recursively enumerable formal system *F* that is sufficiently capable of representing basic arithmetic, there exist statements *s* in the language of *F* such that: *F*⊬*s* and *F*⊬¬*s*, yet *s* is true (“true” is meant in the standard model of arithmetic) in the standard model ℕ. Note, this is a proven theorem and is called here as an Axiom as it is established as true. Axiom 2 (Church-Turing Thesis): Every effectively computable function (i.e., every function can be computed by a physical or algorithmic process in a finite amount of time for each input) is Turing complete. Formally, if function *f* : Σ* → {0,1} is decidable by some idealized mechanical procedure, then there exists a Turning machine *M* that decides *f* (the thesis is a philosophical statement supported by strong evidence, even though it is not mathematically provable). Axiom 3 (Undecidability of the Halting Problem): The Halting Problem *H* is defined as follows: given a description of a Turing machine *M*′ and an input *w*, determine whether *M*′ halts on *w*. It is known that there is no Turing machine *H_M_* that solves this problem for all *M*′,*w*. Formally, *H* is undecidable: ∀*M*:*M* does not decide *H*. Axiom 4 (standard quantum computation model): A quantum computation can be modeled by: (1) A finite number of qubits initialized in a known state (e.g., *f*|0⟩^⊗n^). (2) A finite sequence of unitary gates (from a finite universal gate set) acting on these qubits. (3) A measurement in a fixed computational basis at the end of the computation (the choice of the universal gate set is such that any polynomial-time quantum computation can be approximated to arbitrary accuracy using circuits of polynomial size). Axiom 5 (BQP ⊆ PSPACE): The class BQP (problems solvable by bounded-error quantum polynomial-time algorithms) is contained in PSPACE. That is, for every language *L* ∈ BQP, there exists a deterministic Turing machine that decides *L* using only polynomial space. Note: although we call this an “Axiom” for stylistic consistency, BQP ⊆ PSPACE is a proven theorem (above) within quantum complexity theory and is established as true. It is also established as true via supportive evidence from Bennett et al. (1997).

Theorem (quantum undecidability theorem): No quantum algorithm can decide the truth value in the standard model ℕ of any arithmetic statement *s* that is unprovable in a given consistent, recursively enumerable formal system *F* (capable of representing basic arithmetic), solely by utilizing quantum superposition, entanglement, and interference.

Proof by contradiction: Step 1 (assumption of quantum decidability): By Axiom 1, we know that in any sufficiently strong, consistent formal system *F* there exist Gödelian statements *s*, that is, statements that are true in the standard model ℕ but unprovable in *F*. Assume, for the sake of contradiction, that there exists a quantum algorithm *Q* (constructed according to Axiom 4) such that for every arithmetic statement *s* in the language of *F* that is unprovable in *F*, *Q* halts and outputs: $Q\left(s\right)=\{\begin{array}{c} 1\;if\;s\;is\;true\;in\text{ N}\\ \text{0 }if\;s\;is\;false\;in\text{ N} \end{array}$. Thus, *Q* decides the truth of every Gödelian statement of *F*. Step 2 (Simulation by a Classical Machine via Axiom 2): By Axiom 2 (Church-Turing Thesis), every effective algorithm is computable by a Turing machine. Since *Q* is an effective procedure, there exists a deterministic Turing machine *M_Q_* that simulates *Q*. Step 3 (encoding the Halting problem instances via Axiom 3): Using standard arithmetization techniques, which are closely connected with the undecidability of the Halting Problem as stated in Axiom 3, for any Turing machine *M*′ and input *w* one can effectively construct an arithmetic sentence φ(*M*′,*w*) such that φ(*M*′,*w*) is true in ℕ⟺*M*′ halts on input *w*. Moreover, by the standard construction (in line with Axiom 1), if *M*′ does not halt on *w*, then φ(*M*′,*w*) is arranged to be unprovable in *F*. Step 4: (Applying *Q* to Halting Problem Encodings): For any Turing machine *M*′ and input *w* such that φ(*M*′,*w*) is unprovable in *F*, our assumption (from Step 1) implies that $Q\left(\text{φ}\left(M',w\right)\right)=\{\begin{array}{c} 1\;if\;s\;is\;true\;in\text{ N }\left(i.e.,\;M'\;halts\;on\;w\right)\\ \text{0 }if\;s\;is\;false\;in\text{ N}\left(i.e.,\;M'\;does\;not\;halt\;on\;w\right) \end{array}$. Since *M_Q_* simulates *Q* (from Step 2), running *M_Q_* on φ(*M*′,*w*) would effectively decide the halting behavior of *M*′ on *w*. Step 5 (Deriving a Contradiction using Axiom 3): By Axiom 3, the halting problem is undecidable by any Turing machine. Yet, through *M_Q_* we have constructed a deterministic procedure that decides whether *M*′ halts on *w* [for those cases where φ(*M*′,*w*) is unprovable in *F*]. This contradicts the undecidability stated in Axiom 3. Step 6 (Conclusion): The contradiction reached in Step 5 forces us to reject our initial assumption (Step 1). Therefore, no quantum algorithm *Q* exists that can decide the truth value of every arithmetic statement *s* (that is unprovable in *F*) solely by using quantum superposition, entanglement, and interference.

It is a standard result in computability theory (which follows from Axiom 2, the Church-Turing Thesis, and well-known arithmetization techniques) that for any Turing machine *M*′ and input *w*, we can construct an arithmetic statement *ϕ*_*M’,w*_ such that: *ϕ*_*M*′,*w*_*is true in* ℕ⟺*M*′ *halts on input w*. The construction of such *ϕ*_*M’,w*_ is a known technique in recursion theory and logic. Since by Axiom 2 all effectively checkable computations are Turing-computable, and by classical recursion theory (a standard extension of Axiom 2’s framework), any Turing computation can be encoded into a statement of arithmetic. Step 4 (linking Gödelian statements to Halting): Now, consider the problem of deciding the truth of statements like *ϕ*_*M’,w*_ for arbitrary *M*′, *w*. If *Q* can decide the truth value of any Gödelian unprovable statement *s* in *F*, then we can arrange (via appropriate constructions of arithmetic statements and reflection principles) for *ϕ*_*M’,w*_ to behave similarly to Gödelian statements for a sufficiently strong system *F*.

That is, if *F* cannot decide *ϕ*_*M’,w*_, but *Q* can determine its truth, then *Q* would effectively be solving the Halting Problem *H*. By Axiom 1, there exist statements *s* is unprovable in *F*. With standard techniques in logic (Gödel numbering, the Recursion Theorem), the truth of *ϕ*_*M’,w*_ corresponds to the halting of *M*′ on *w*. If *Q* can handle any statement that *F* cannot settle including such *ϕ*_*M’,w*_, then *Q* decides *H*. Step 5 (undecidability of the Halting problem): By Axiom 3, no Turing machine can decide *H*. Therefore, if a method (even a quantum one) existed to decide all such *ϕ*_*M’,w*_, it would contradict Axiom 3. Axiom 3 directly states that the Halting Problem is undecidable. If *Q* decides *ϕ*_*M’,w*_ for every *M*′, *w*, then *Q* decides *H*. This cannot happen. Step 6 (BQP ⊆ PSPACE): Since *Q* is a quantum algorithm, it decides a language in BQP. By Axiom 5 (BQP⊆PSPACE), there exists a deterministic Turing machine *M_Q_* that uses only polynomial space and simulates the input-output behavior of *Q*. Axiom 5 ensures that any BQP language can also be decided by a PSPACE machine, so if *Q* exists, *M_Q_* simulates *Q*. Step 7 (contradiction): If *M_Q_* simulates *Q* and *Q* solves the Halting Problem by deciding *ϕ*_*M’, W*_, then *M_Q_* also solves *H*. This contradicts Axiom 3, which states that no Turing machine can solve *H*. We, therefore, deduced that a polynomial-space deterministic Turing machine solves *H*, contradicting Axiom 3. Step 8 (conclusion): We have reached a contradiction under the assumption that such a quantum algorithm *Q* exists. By reductio ad absurdum, our initial assumption (that there exists a quantum algorithm *Q* capable of determining the truth value of any Gödelian unprovable statement *s* within a sufficiently strong and consistent formal system *F*) is false. Therefore, no quantum algorithm can determine the truth value of Gödelian unprovable statements.

The proof demonstrates via reductio ad absurdum (Proof by contradiction) that quantum algorithms cannot determine the truth value of a mathematical statement *s* that is unprovable within a formal system *F*, solely by utilizing quantum superposition, entanglement, and interference. It does so by assuming the contrary and showing that this leads to a contradiction with established principles in computability theory and quantum mechanics. The proof shows that quantum algorithms (bound by the Church-Turing Thesis that establishes the limits of what can be computed by any algorithmic process, including quantum algorithms) cannot determine the truth value of mathematical statements that are unprovable within a formal system *F*. This is because (1) quantum algorithms are bound by the same computability limits as classical algorithms. (2) Unprovable statements lack proof within the formal system, preventing the construction of effective oracles necessary for certain quantum algorithms. (3) Quantum mechanics linearity and unitarity do not permit deterministic resolution of undecidable problems. Thus, even with the advanced capabilities of quantum computation, the fundamental limitations of logic and computability theory prevail.

Penrose, however, is referring to some exotic (non-standard QM), and unproven quantum gravity objective reduction of the quantum state (as in his “OR” theory) whereby such a collapse is non-computational and therefore is outside of the laws of computability. Penrose believes that gravity, the curvature of spacetime, plays a crucial role in causing an Objective Reduction (OR) of the quantum state. The idea is that when mass distributions are in quantum superposition, they effectively create competing curvatures of spacetime. According to Penrose, there is a fundamental instability, whereby nature does not like sustained superpositions at the gravitational scale. After a very short time determined by the mass and gravitational self-energy differences, the superposition will spontaneously collapse (reduce) to a single state. Penrose (a mathematical Platonist) believes mathematical truths exist in a timeless, abstract realm (the Platonic world), and that physical reality, especially at the fundamental quantum gravitational level, is closely aligned with these truths. He, therefore, postulates that objective reductions in the brain could thus resonate with or reflect these truths, granting the mind a direct but non-verbalized sight of them (these truths).

However, one criticism of this postulate is that if quantum collapses are fundamentally probabilistic, then consciousness, built on such collapses, should be nothing more than random noise. This of course is not the world we experience, we experience a highly ordered, continuous, and linear conscious experience. Hameroff and Penrose propose that tubulin states, organized in complex arrays, can bias the collapse outcomes (Hameroff and Penrose, 2014; Hameroff and Penrose, 1996). In other words, while the raw OR event might have multiple possible outcomes, the microtubule environment “selects” or constrains these outcomes so that they are more ordered, which according to them explains why consciousness is ordered and continuous rather than random. So, Penrose’s argument is that there may be some new physical laws or phenomena that could break free from the standard computational paradigm, however, this is entirely speculative and unproven. Even if OR was determined to be an accurate description of physical reality, it has not been shown how one would harness this form of collapse to identify truth that bypasses computations and surpasses the Church-Turing thesis. Gödelian statements are not just hard problems, they represent fundamental logical limits of deduction and mechanical computation. The onus would therefore be on Penrose to prove how some yet-to-be-discovered exotic quantum gravity or some form of objective reduction of the quantum state can surpass the proofs outlined here in order to obtain truth.

One potential solution to this is to adopt a functional contextualist *N*-Frame (natural selection, neurobiological, relational frame theory) (Edwards, 2023, 2024) approach that integrates the observer’s context directly into the quantum formalism, which may lead to the establishment of truths beyond formal proof. *N*-Frame (Edwards, 2023, 2024) is a functional contextual account of cognition and human behavior that assumes a perceptual (phenomenological) conscious interface via evolutionary game theory replicator equation (Taylor and Jonker, 1978) and corresponding evolutionary game theory simulations (Hoffman and Prakash, 2014; Hoffman et al., 2015), predictive coding of neuroscience (Friston, 2010; Friston and Kiebel, 2009; Smith et al., 2021), and Bayesian subjective dynamics of quantum mechanics based on QBism (Fuchs, 2014, 2023).

As there is a clear limitation in what QM can achieve when attempting to overcome finding truth in these Gödelian unprovable statements, and because Penrose does not articulate a precise QM model to overcome this, this theory and hypothesis paper proposes an *N*-Frame (Edwards, 2023, 2024) account which may in part be able to show how truth can be found without proof. *N*-Frame proposes this via its framework that fits with current state-of-the-art theoretical physics theories such as the holographic principle in the Anti-de Sitter/Conformal Field Theory (AdS/CFT) model (Maldacena, 1999). This paper gives a clear pathway for experimentation and some clear hypotheses that may allow for empirical results to support this ambitious theoretical approach, as well as some basic simulations of existing data. It will also attempt to account for the measurement problem within its theoretical approach expanding on the von Neumann (1932) conscious chain, and conscious collapse (or actualization) approaches more generally (Campbell, 2007; Chalmers and McQueen, 2021; Kauffman and Radin, 2023; London and Bauer, 1939; Stapp, 2004, 2007; von Neumann, 1932; Wheeler, 1992; Wigner, 1961).

## 2 An overview of the *N*-Frame model

**FIGURE 1:**
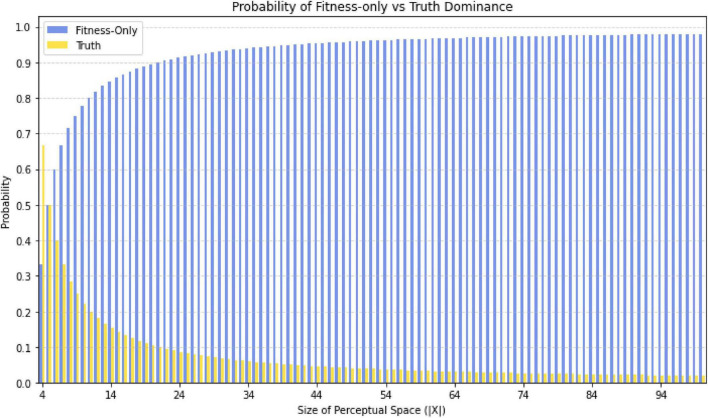
Graph illustrating the probability of the fitness-only strategy vs. truth dominance, as part of the fitness beats truth (FBT) theorem $\frac{\left|X\right|-3}{\left|X\right|-1}$. This allows for a perceptual interface (the map) and not the territory (the real world)—i.e., what we see perceptually is not homomorphic to a “real” world according to evolutionary game theory simulations.

*N*-Frame (Edwards, 2023, 2024) adopts the findings of the evolutionary game theory simulations (Hoffman and Prakash, 2014; Hoffman et al., 2015) and is in line with Hoffman et al.’s conclusion regarding an “interface theory of perception” (Hoffman, 2016, 2018; Hoffman et al., 2015). The “interface theory of perception” is also supported by the “fitness beats truth” (FBT) proof (Prakash et al., 2021), that proves natural selection does not favor perceptions that accurately reflect objective reality, instead, it favors perceptual systems that maximize an organism’s fitness, that is, its ability to survive and reproduce (see Figure 1^1^ for outputted simulation of this dominance over time of fitness using evolutionary game theory), and this interface theory is adopted in *N*-Frame as a crucial component of the theory (Edwards, 2023, 2024). The basic idea behind FBT (Prakash et al., 2021) can be expressed via the equation $\frac{\left|X\right|-3}{\left|X\right|-1}$, whereby perceptual space *X* is the set of all possible perceptual states or subjective experiences that an organism can encounter through their sensory systems. |*X*| denotes the cardinality or number of elements in this set *X*, and represents how many distinct perceptual states the organism can discriminate and experience. If an organism can experience just two perceptual states such as some single-celled Euglena which has a light-sensitive detector called an eyespot, which allows them to move away or toward a light source (phototaxis), then |*X*| = 2. A healthy young human adult can detect some 10 million colors alone, hear frequencies between the range of 20 Hz to 20 KHz, sense proprioception, interoception, vestibular sense, touch, smell, taste, as well as complex emotional and experiential states, so this could approach infinity (but never actually reaching infinity given the finite nature of the organism) |*X*| = [*X*,∞]. This captures the idea that the human perceptual space is extremely high-dimensional and rich, with an immense number of possible distinct perceptual states we can experience, but not necessarily infinite. The notation $\frac{\left|X\right|-3}{\left|X\right|-1}$ expresses that as |*X*| gets larger and larger, representing a more complex perceptual space with many possible distinct phenomenological experiences, the probability that natural selection favors a “Truth” perceptual strategy (accurately representing reality) diminishes in comparison to a “Fitness-only” strategy tuned solely for evolutionary success.

A key difference with the Hoffman interface approach (though not likely inconsistent with their interpretation), is the focus of the conscious internal (to the universe) observers *C*_*intO*_ (i.e., us human and other organisms within the universe that can be described as conscious), whilst Hoffman et al., (Hoffman, 2016, 2018; Hoffman et al., 2015) are focused on modeling a minimal description of conscious agent dynamics that lead to particle interactions and the laws of the universe, i.e., these are external to the universe, and the universe is formed as an emergent property (and interface) of these more fundamental conscious dynamics (somewhat like a virtual reality experience). Their “conscious agent” framework (Hoffman, 2016; Hoffman et al., 2023) (often linked to his broader ideas of “conscious realism”) in which conscious entities and their interactions are taken as the fundamental building blocks of reality. The idea is that, rather than starting with matter/particles as primitive and trying to explain consciousness, one begins with consciousness and attempts to derive or explain the appearance of particles, fields, and physical laws from the dynamics of these conscious agents. *N*-Frame (Edwards, 2023, 2024) takes a more pragmatic (in line with functional contextualism) approach, that we should be agnostic about whether physicalism leads to consciousness (physicalist approach), or consciousness should lead to physicalism (idealism approach), and instead, we should functionally start with the conscious experience of the observer *C*_*intO*_ and deduce all other things about reality from that perspective.

From this functional contextual *N*-Frame (Edwards, 2023, 2024) perspective, some key assumptions about these conscious (internal to the universe) observers *C*_*intO*_

(e.g., observers like us humans) can be given by *N*-Frame’s *conscious elements of an internal observer of a system*. First, inspired by axiomatic approaches of Euclid’s Euclidean geometry book *The Thirteen Books of the Elements* (Heath, 2013) (originally written in 300BC by Greek philosopher Euclid in ancient Alexandria of Hellenistic Egypt) we will attempt to establish fundamental axioms that formalize consciousness within a quantum framework that offers a structured and rigorous foundation for exploring the intricate relationship between consciousness, quantum measurement, and the physical world, akin to those in Euclidean geometry “*The Elements*.” Using deductive reasoning Euclid sought to build geometry from a set of axioms (self-evident truths) and postulates (assumptions specific to geometry). This method ensures that all subsequent propositions and theorems are logically derived from these foundational statements. The axioms serve as the foundational building blocks from which all other statements, definitions, and theorems about consciousness can be derived. Understanding the role and nature of axioms is crucial to structuring a coherent and logically consistent framework. By establishing a fundamental axiomatic approach, we ensure that the system is clear, consistent, and scalable. This method not only aligns with QBism’s (Fuchs, 2014, 2023) emphasis on the subjective and observer-dependent nature of quantum states but also provides a solid base for further theoretical and empirical exploration. *The conscious elements of an internal observer of a system* are stated as follows:

First, we need a language *L* that provides the vocabulary (with its domain, syntax, and predicates) necessary to articulate these ideas, and the axioms serve as foundational assumptions for further theoretical exploration. The domain of discourse *D* for *L* is taken to include all entities relevant to our discussion, namely conscious entities (such as humans and potentially other sentient beings), physical systems (objects or events governed by physical laws), and abstract objects (such as mathematical entities or propositions). The language *L* is built from the usual logical apparatus: variables *x*, *y*, *z*, ranging over *D*; logical connectives ∧,∨,→, and ¬; quantifiers ∀ and ∃; and the equality symbol . In addition, *L* includes several predicate symbols designed to capture key concepts. These include, *Conscious*(*x*) denotes that *x* is a conscious entity, that is, *x* possesses subjective, first-person experience. The predicate ExperiencesClassical(*x*) asserts that *x* experiences the world in a determinate, classical manner, meaning that the direct perceptual input of *x* is always a single, definite outcome rather than a quantum superposition. Further, the predicate ConstrainedByLaws(*x*,*L*) expresses that *x* perceives itself as operating under the constraints imposed by a set *L* of physical laws (for example, the laws of gravity, electromagnetism, and thermodynamics). To capture the ability for abstract thought, we include AccessesMathematics(*x*), which indicates that *x* can engage in abstract mathematical reasoning, providing access to a “Platonic realm” of mathematical truths. Finally, to address computational limits, the language includes EffectivelyComputable(*f*) and TuringComputable(*f*), predicates that express that a function *f* is computable by some effective process and by a Turing machine, respectively.

Within this formal framework, we adopt the following self-evident axioms as foundational assumptions in relation to human consciousness on the whole: Axiom 1 (Existence of Conscious Entities): ∃*x Conscious* (*x*). This axiom formalizes the self-evident nature of subjective experience by asserting that at least one entity in *D* is conscious. Axiom 2 (Classical Nature of Direct Experience): ∀*x* (*Conscious*(*x*) → *ExperiencesClassical*(*x*)). This axiom captures the observation that every conscious entity experiences the world in a definite, determinate manner, without direct perception of quantum indeterminacy. Axiom 3 (Constrained Existence Within a Physical System): Let *L*_*phys*_ denote a fixed set of physical laws (e.g., gravity, electromagnetism, thermodynamics). Then, ∀*x*(*Conscious*(*x*) → *ConstrainedByLaws* (*x*,*L*_*phys*_)). This axiom expresses that every conscious entity perceives itself as operating within a universe governed by immutable physical laws. Axiom 4 (Epistemological Access to the Platonic Realm): ∀*x* (*Conscious*(*x*) → *AccessesMathematics*(*x*)). This axiom postulates that conscious entities have the capacity for abstract reasoning, enabling them to access and explore mathematical structures that reveal deeper aspects of reality beyond direct sensory experience. Axiom 5 (Church–Turing Thesis: Effective Computability): ∀*f*[*EffectivelyComputable*(*f*) → *TuringComputable*(*f*)]. This final axiom reflects the widely accepted Church–Turing Thesis, stating that every function that is computable by any effective process is also Turing-computable.

The Church–Turing Thesis (Axiom 5) might seem, at first, to be about computation rather than consciousness. However, it is included because it sets a fundamental limit on any algorithmic or effective process, including those that might occur in conscious entities. In our framework, conscious entities are assumed to engage in abstract reasoning and mathematical thought (as per Axiom 4). If these processes are effective computations (that is, they can be carried out by some algorithmic procedure), then by the Church–Turing Thesis they must be Turing-computable. This means that any computation performed by a conscious mind is, in principle, within the limits of Turing-computability. Thus, Axiom 5 helps bridge the study of consciousness with computational theory by asserting that the computational aspects of conscious processes adhere to the same limits as any effective algorithm. It provides a formal constraint on what a conscious entity can compute or decide, ensuring that even abstract reasoning remains within a framework that is mathematically well-understood.

From these self-evident axioms, several additional postulates (assumptions) can be articulated that are relevant to the *N*-Frame (Edwards, 2023, 2024) perspective of reality. Here, we can extend our formal language *L* to an enriched language *L*′ that is capable of capturing additional aspects (postulates) of the *N*-Frame perspective. In this extended language, the domain of discourse includes not only conscious entities, physical systems, and abstract objects but also distinct sorts for quantum states, classical states, computational boundaries, and systems. As such, new predicate symbols are introduced, such as *QuantumState*(*q*) to denote that *q* is a quantum state, *ClassicalState*(*s*) to indicate that *s* is a classical state, and *GeneratesClassical*(*q*,*s*) to express that the quantum state *q* gives rise to the classical state *s*. Similarly, predicates like *FocusesOn*(*x*,*q*)and *Collapses*(*q*)capture the idea that when a conscious entity *x* directs attention to a quantum state *q*, the state collapses into a definite classical outcome. The predicate *ComputationalBoundary*(*x*,*b*) formalizes the notion that each conscious observer *x* is endowed with a bounded information-processing capacity *b*, while *PartOfSystem*(*x*,*S*) indicates that *x* is an integral component of the system *S* it observes.

Moreover, the predicate *Experiences*(*ϕ*,*x*) is used to denote that *x* experiences a subjective state ϕ, with *Corresponds*(*ϕ*,*q*) specifying that the subjective state ϕ is associated with the quantum state *q*, and *ModulatedBy*(*q*,*x*) indicating that the mapping of *q* is influenced by properties of *x*.

Within this extended language, we postulate the following: (1) Every classical state arises from some underlying quantum state, formally, ∀*s*{*ClassicalState*(*s*) → ∃*q*[*QuantumState*(*q*) ∧ *GeneratesClassical*(*q*,*s*)]}; (2) All interactions between a conscious observer and events are mediated by quantum states, i.e., ∀*x*∀*E*{*Observes*(*x*,*E*) → ∃*q*[*QuantumState*(*q*) ∧ *Mediates*(*x*,*q*,*E*)]}; (3) Every subjective state experienced by an observer corresponds to a quantum state, expressed as ∀*x*∀*ϕ*{*Experiences*(*ϕ*,*x*) → ∃*q*[*QuantumState*(*q*)∧*Corresponds*(*ϕ*,*q*)]}; (4) The act of focusing attention on a quantum state induces its collapse, denoted as ∀*x*∀*q*[*Conscious*(*x*)∧*FocusesOn*(*x*,*q*) → *Collapses*(*q*)]; (5) Each conscious observer possesses a computational boundary, ∀*x*[*Conscious*(*x*) → ∃*bComputationalBoundary*(*x*,*b*)]; (6) Subjective states are context-dependent, being modulated by the observer’s internal states and computational limits, captured by ∀*x*∀*ϕ*[*Experiences*(*ϕ*,*x*) → *ContextDependent*(*ϕ*,*x*)]; (7) The mapping between subjective states and quantum states is modulated by the observer’s computational boundary, i.e., ∀*x*∀*ϕ*∀*q*{[*Experiences*(*ϕ*,*x*)∧*Corresponds*(*ϕ*,*q*)] → *ModulatedBy*(*q*,*x*)}; (8) Every conscious observer is an integral part of the system it observes, thereby enabling self-referential observation, formalized by ∀*x*{*Conscious*(*x*) → ∃*S*[*PartOfSystem*(*x*,*S*)∧*SelfObserving*(*S*)]}; (9) A fundamental epistemological limit exists for each conscious observer, determined by its computational boundary, namely, ∀*x*{*Conscious*(*x*) → ∃*b*[*ComputationalBoundary*(*x*,*b*)∧*EpistemologicalLimit*(*x*,*b*)]};; (10) For every conscious observer *x*, there exists a hypercomputational process *H*, intrinsic to *x*’s conscious observation, such that for some function *f* that is effectively computable, *H*(*f*) is not Turing-computable, denoted as ∀*x*{*Conscious*(*x*) → ∃*H*[*IntrinsicHypercomputaion*(*H*,*x*)∧∃*f*[*EffectivlyComputable*(*f*)∧¬TuringComputable[H(f)]]]}.

Postulates 5 and 9 are linked to Axiom 5. The connection lies in the fact that Axiom 5, the Church–Turing Thesis, asserts that every effectively computable function is Turing-computable, thereby establishing inherent limits on all algorithmic processes. Since we assume that conscious observers perform information-processing tasks effectively (for example, in abstract reasoning or perception), these tasks are subject to the same computational constraints as any algorithmic process. In our formal *N*-Frame (Edwards, 2023, 2024) framework, Postulate 5 states that each conscious observer internal to the universe *C*_*intO*_ has a computational boundary that limits its information-processing capacity. This postulate reflects the idea that the cognitive processes of conscious entities, being effective processes, are subject to the same limits as any Turing-computable function. In other words, if a conscious entity *C*_*intO*_ processes information via effective (algorithmic) means, then the finite resources and constraints inherent in Turing machines also constrain that entity. Thus, the computational boundary described in Postulate 5 is a natural consequence of the computational limits set out by Axiom 5, and it serves to ground our model of consciousness within a framework that respects the fundamental limits of computation. Postulate 9 builds on this idea by stating that each conscious observer has a computational boundary that limits its information-processing capacity and thus its epistemic access to the system. In other words, the fundamental epistemological limit imposed on a conscious observer is, in part, a consequence of the limitations set by Turing-computability, i.e., any computation that the observer performs must abide by the constraints inherent in Turing machines. This linkage formalizes the idea that the observer’s ability to process, store, and reason about information is not arbitrary but is bounded by the fundamental limits of computation as described by Axiom 5.

To ensure consistency, one must interpret the observer’s computational boundary (in Postulates 5 and 9) as describing the Turing-computable portion of the observer’s processing, while Postulate 10 introduces an extra, non-algorithmic capacity. In other words, the *N*-Frame model assumes that a conscious observer exhibits both standard effective (Turing) computation and, additionally, possesses a hypercomputational facet (see Supplementary 1 for a revised hypercomputational Church-Turing thesis). Although this is a speculative extension beyond conventional computation, it can be made consistent as long as the hypercomputational process is treated as a separate but coexisting component. Thus, within this extended formal language, the postulates as stated provide a self-contained, logically coherent framework for discussing how quantum processes, observer-centric information, and computational limits interrelate. Together, these extended postulates complement our initial axioms by establishing a comprehensive formal scaffold that interrelates quantum foundations, classical emergence, and the intrinsic limitations of conscious observation.

It should also be noted that specific conscious internal (to the universe) observer *C*_*intO*_ dynamics can be modeled by *N*-Frame (Edwards, 2023, 2024). *N*-Frame assumes observer-centric realism, whereby the observer is at the heart of the reality we see self-referentially consistent with postulate 8 of “*The conscious elements of an internal observer of a system*,” and also consistent with Wheeler’s “it from bit” (Wheeler, 1992), and also the self-simulation model (Irwin et al., 2020). The key idea of this is that the universe as a system can be thoughts of a self-generative system that evolves conscious observers (organisms like us) that have some computational boundary (e.g., different types of organisms have different informational boundaries, such as humans have a larger computational boundary than a dog), and these observers internal to the universe *C*_*intO*_s, observer the universe, and this is akin to saying that the universe is observing itself through my (or your) own perspectives as internal observers *C*_*intO*_s (Faggin, 2019, 2021) (these arguments are where postulate 8 originates from). Crucially, this is consistent with Gödel incompleteness theorems, Penrose’s non-computable phenomena in consciousness and fundamental physics conjecture, and the self-simulation hypothesis (Irwin et al., 2020). Together, they converge on the idea that truths transcend computation in self-referential systems. Consciousness plays a central role in this process, navigating and generating truths beyond the limits of formal systems. The *N*-Frame (Edwards, 2023, 2024) framework suggests a recursive, self-referential loop that naturally integrates Gödelian and non-computable phenomena, presenting reality as a self-evolving, self-actualizing conscious system. The ideas extend existing perspectives of QM such as von Newmann’s and others that consciousness acts as a measuring process that collapses the wave function (Campbell, 2007; Chalmers and McQueen, 2021; Kauffman and Radin, 2023; London and Bauer, 1939; Stapp, 2004, 2007; von Neumann, 1932; Wheeler, 1992; Wigner, 1961) to a self-referential process more in line with Wheeler’s “it from bit” (Wheeler, 1992) whereby the act of observation is not passive, rather it is an active participation in the creation (or actualization) of reality.

**FIGURE 2:**
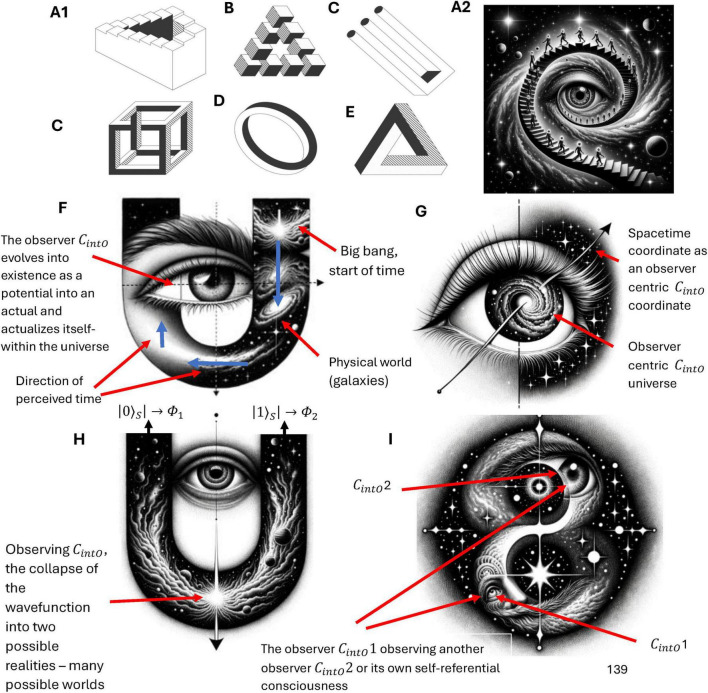
**(A1–E)** Illustrates impossible structures (or objects) based on continuous self-referential loop paradoxes (internal only observer), whereby the internal observer can get caught in paradoxes that have no biggening or end. Ψ → Φ ≡ *C*_*intO*_ ≡ *P*. **(A2)** is the Escher stairs again but this time demonstrating that external objects are not just related to the external properties of objects being observed in the physical world, but also related to the internal states and behaviors of the observer (observer-centric *C*_*intO*_). **(F)** illustrates Wigner’s it from bit, the participatory universe (cosmological evolution) self-reference; **(G)** illustrates spacetime expressed as an observer coordinate; **(H)** illustrates the collapse of the waveform from *C*_*intO*_; **(I)** illustrates *C*_*intO*_ observing another *C*_*intO*_ or itself self-referentially. Note adobe stock images **(A1-E)** from user Elena with permission.

Self-referential loops and their link to consciousness, Gödel incompleteness theorems, and loops in nature such as Bach’s music have been long established by Douglas Hofstadter (Hofstadter, 1999, 2007). For example, he identified self-referential recursive structures that create a sense of infinite regression or interwoven cycles within Bach’s compositions such as in “Canon a 2 per Tonos” from “The Musical Offering BWV 1079,” and “Crab Canon” in “The Musical Offering, BWV 1079,” whereby this canon is a musical palindrome. The two voices can be played simultaneously, one forward and the other backward creating a harmonious and interlocking structure. Hofstadter also identified similar strange infinite regressive loops in the work of Escher art (see Figures 2 A1–E) have been highlighted previously by physicist Douglas Hofstadter (Hofstadter, 1999, 2007). The Escher strange loop images, Bach’s music, Gödel incompleteness theorems all highlight a type of strange self-referential loop of an infinite regress in different modalities (formal math and logic, music, and art). These self-referential loops is further instantiated in *N*-Frame (Edwards, 2023, 2024), as the self-referential loops of consciousness and physical world that we perceive (i.e., the universe as a system that we as observers *C*_*intO*_ exist and perceive as reality) in line with Wheeler’s conscious observer participatory reality (Wheeler, 1992; see Figures 2, A2, F–I).

**FIGURE 3:**
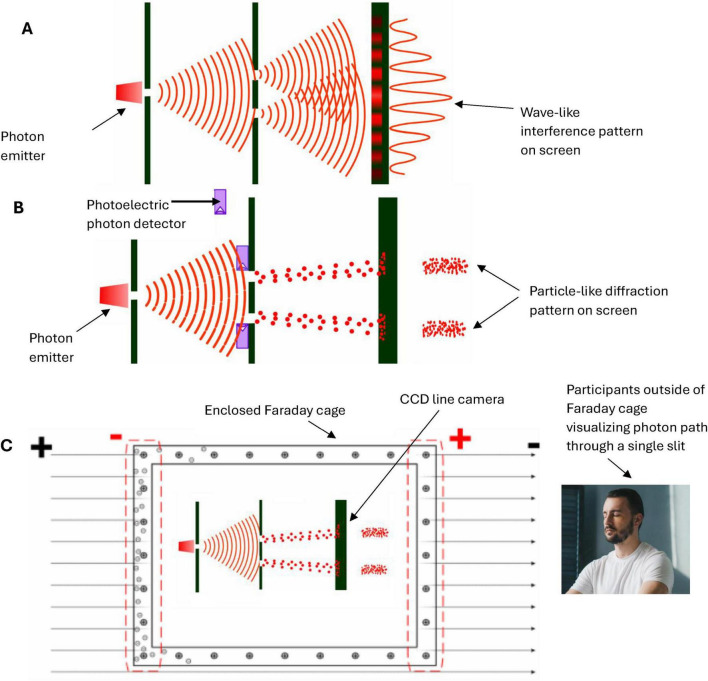
**(A)** illustrates an interference pattern observed in the classic Young Double slit experiment whereby the photon evolving through the double slits behaves like a wave rather than a particle, leading to an interference pattern. **(B)** illustrates a modified version of the classic Young Double slit experiment whereby a photoelectric detector is placed at the *entry point* of the double slits, and this placement of detectors leads to the photon behaving more particle-like, leading to a two-band diffraction pattern. **(C)** illustrates a modified version of the classic Young Double slit experiment whereby the double-slit experiment is now inside an enclosed Faraday cage, and the participant is outside the cage. There is no physical photoelectrode detector at the slits this time, the participant simply visualizes (focused attention) which slit the photon passes through, and perturbations in the waveform are recorded by a CCD line camera at the end screen. Adobe stock images [**(A,B)** and left inner part of **(C)**] from user LuckySoul. Person in C by Strelciuc. Faraday cage from Stanisław Skowron. All images are used with permission.

*N*-Frame (Edwards, 2023, 2024) consistent with the self-simulation hypothesis (Irwin et al., 2020) take this one step further suggesting that as the conscious observer *C*_*intO*_ actualizes the quantum information (qubits) into a definite collapsed state. In doing so, the observer *C*_*intO*_ bridges the gap between the potential (quantum superposition) and the actual (definite state), embedding meaning and structure into the self-referential system of reality. This line underscores the frameworks’ emphasis on consciousness as a creative agent, shaping the informational substrate of reality through observation. It should be noted that there is growing empirical support with up to a 5 Sigma significance that consciousness intent can collapse the waveform rather than a physical detector (Baer, 2015; Bierman, 2003; Bösch et al., 2006; Ibison and Jeffers, 1998; Radin, 2008; Radin and Delorme, 2021; Radin et al., 2015a; Radin et al., 2016; Radin et al., 2012; Radin et al., 2013; Radin et al., 2015b; Radin et al., 2019; Vieten et al., 2018). For these studies, see for example, Figures 3A, B indicating a double slit experiment with no detector leading to an interference pattern 3A, a double slit experiment with a detector causing the collapse of the wavefunction and removing the interference pattern 3B, and a double slit experiment inside a Faraday cage where a human outside the cage visualizes mentally the path of the photon causing perturbation of the wavefunction reducing the interference pattern (see Figure 3C). This is encouraging as this is the same significance level (5 Sigma) as was found by a CERN research team at the Large Hadron Collider who confirmed the process that gives mass to elementary particles via spontaneous symmetry breaking, now known as the Higgs mechanism. This mechanism predicts the existence of the Higgs boson, which was later confirmed at CERN. François Englert and Peter Higgs who both independently predicted this, both won the *Nobel prize* for this CERN confirmation of the *Higgs boson* with a 5 Sigma. Much replication work of these conscious collapse studies is required before a seismic shift in physics leads them to explore this more closely, despite the 5 Sigma findings.

In these “consciousness causes collapse” experiments, human participants are asked by Raiden and colleagues to imagine which slit the electron passes through in a double slit or interferometer experiment, whereby the experimenter identifies whether their conscious intent can collapse the wavefunction into a particle-like state Ψ→Φ (see Figure 3C). These experiment outcomes are consistent with predictions made by *N*-Frame’s (Edwards, 2023, 2024) observer-centric *C*_*intO*_ interpretation central to a participatory universe (Wheeler, 1992) and crucially to an observer-centric particularly realism perspective and is relevant for testing consciousness in both humans and AI (Edwards, 2024).

**FIGURE 4:**
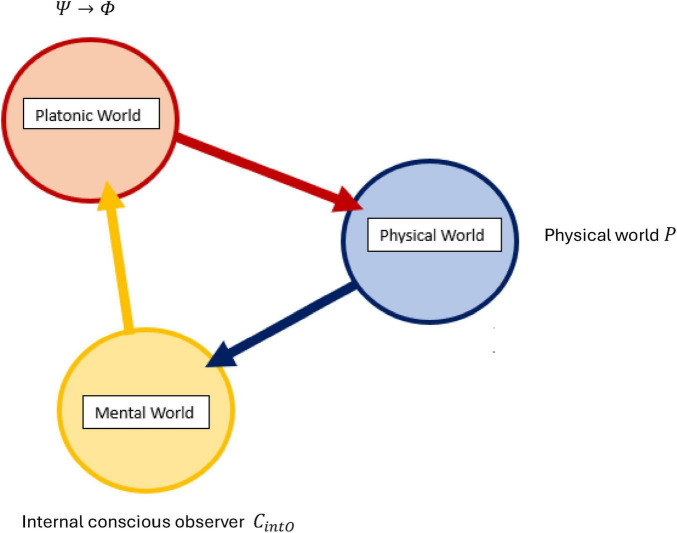
An updated illustration of Penrose’s theory of the three worlds (like three sides of a three sided coin), the interface comprises of a tri-aspect monism which highlights the circular relation of the platonic world Ψ→Φ, the physical world *P*, and the mental world *C*_*intO*_ which gives a deeply interconnected (equivalence) account for a conscious epistemic observer-centric (participatory) ontological realism Ψ → Φ ≡ *C*_*intO*_ ≡ *P*.

*N*-Frame (Edwards, 2023, 2024) (in line with self-simulation hypothesis) framework aligns with the idea that reality from the observer’s perspective and in line with the axioms of “*The conscious elements of an internal observer of a system*” is observed information, as “reality” from this perspective is essentially the outcome of a process in which conscious observers transform underlying quantum potentialities into definite, structured information. In this view, the quantum state Ψ which represents a superposition of possibilities becomes actualized as a classical state Φ through the observer’s interaction with the system Ψ→Φ. This transformation is not a passive occurrence, rather, it is an active process in which the conscious observer (denoted *C*_*intO*_) encodes and processes information according to its inherent computational boundaries. These boundaries, which are themselves constrained by the Church–Turing Thesis (Axiom 5) and further elaborated in Postulates 5 and 9, limit the observer’s information-processing capacity and define the extent of its epistemic access to the system. As the observer directs its attention to quantum states, these states collapse into singular, determinate outcomes called eigenstates that are imbued with meaning and structure through symbolic, abstract reasoning (as captured by Axiom 4). The self-referential nature of this process is encapsulated in the tri-aspect monist equivalence principle Ψ → Φ ≡ *C*_*intO*_ ≡ *P* which is an extension of Penrose’s tri-word theory (Penrose, 2006; see Figure 4), and implies that the observed universe, the conscious observer, and the underlying quantum processes are different aspects of the same informational reality. Thus, reality is not an independently existing objective entity but is instead the emergent, encoded information produced by the observer’s recursive self-referential and computational interaction with the more fundamental (to physical reality) quantum substrate.

Within the *N*-Frame (Edwards, 2023, 2024) framework, the recursive, self-referential loop encompasses both the observer and the observed, uniting them in a continuous interplay. The observer’s act of measurement introduces coherence into the system, transforming probabilistic information into tangible patterns that evolve further through recursive feedback. This feedback loop allows reality to emerge as an ever-evolving, self-actualizing narrative, where consciousness navigates and generates truths in a dynamic and context-dependent way, often exhibiting creativity and novelty that are not predetermined by any fixed algorithm. This emergent complexity allows conscious agents to generate and navigate truths that seem to “transcend” the static limitations of isolated computations. The feedback loop thus acts as a process for self-actualization, where potentialities (encoded in quantum superpositions) are recursively actualized into definite states, continuously reshaping the observer’s internal model of reality. From a strict Turing perspective, that is, assuming the Church–Turing Thesis holds, every effective process, including this recursive self-referential system we describe, is Turing-computable. In our *N*-Frame (Edwards, 2023, 2024) framework, each component (observation, information processing, and state collapse) is assumed to be an effective (algorithmic) process. Thus, in principle, the entire self-referential system is Turing-computable. However, there is an important nuance in that while the system is Turing-computable in principle, the emergent properties arising from its self-referential and recursive dynamics can be computationally irreducible. This means that, although a universal Turing machine can simulate the system step-by-step, there may be no shortcut to predict its overall evolution without effectively simulating every step. In practice, this leads to behaviors that appear novel, unpredictable, in any useful sense, even though they remain within the bounds of Turing computability. The physical universe, as a manifestation of this process, reflects the intricate dance of meaning, observation, and self-referential creation. This also ties the universe as a teleological system consistent with other work (Azarian, 2022) as the observers that evolve (reducing localized entropy in the process as greater cosmic evolutionary complexity arises) encode meaning into the information that they observe *C*_*intO*_ as the universe evolves (see Supplementary 2 for full arguments about an evolving teleological universe that are postulated from the *N*-Frame model).

At a higher abstract holographic [linking string theory in Anti-de Sitter (AdS) space to conformal field theories (CFT) on its boundary] AdS/CFT description (Maldacena, 1999) of the observer, the non-computable hypercomputation element (postulate 10) is a vital element of the observer’s $C_{i\,n\,t\,O}^{\prime }\,s$ self-referential loop (postulate 8) that shapes emergent geometry in the form of the physical world *P* that we see Ψ→Φ. In mathematical terms, the emergent bulk geometry (the physical *P* spacetime we observe) is given by a mapping *g* = *F*[*b*,*H*(*b*)], whereby *g* ∈ *G* is the emergent bulk geometry (the physical reality *P* we observe), *b* ∈ *B* denotes the (AdS/CFT) boundary information that is Turing-computable (capturing the standard, algorithmic aspect of the observer’s information) and *H*(*b*) represents the hypercomputational (non-Turning computable) contribution intrinsic to the observer’s *C*_*intO*_ conscious process. This hypercomputational element, *H*(*b*), extends beyond conventional algorithmic limits, encoding non-Turing aspects of the observer’s *C*_*intO*_ internal state and measurement processes. As a result, the output *g* reflects not only the standard, predictable transformations but also novel, emergent properties that arise from the interplay between computable and hypercomputational processes. Thus, the observer both influences and is influenced by the boundary information, creating a dynamic, self-actualizing system where the emergent geometry is continuously reshaped by a recursive loop that naturally integrates hypercomputational (non-computable) capabilities into the structure of reality, and therefore transcends Gödel incompleteness. This hypercomputational component of the observer *C*_*intO*_ may explain why conscious humans as internal observers of the universe are able to grasp truths that, according to Gödel’s incompleteness theorem, are logically unprovable within any given formal system. This aligns with Penrose’s argument (Penrose, 1991, 1994) that human consciousness and mathematical insight involve non-Turing-computable processes, potentially resolving his Gödel incompleteness puzzle.

This *N*-Frame (Edwards, 2023, 2024) formulation naturally yields a coherent mathematical framework for the emergence of Lagrangian mechanics and entropy within our observable universe. This integration of hypercomputational elements into the observer’s *C*_*intO*_ boundary, where the observer is itself embedded, creates a self-referential loop whereby the observer’s enhanced, non-algorithmic processing feeds back into the boundary information, which in turn shapes the bulk geometry. When the full boundary action {including observer-specific terms such as $S_{o\,b\,s}=\int_{\partial {\text{M}}_{o\,b\,s}}d^{d}x\sqrt{-\gamma }[L_{\text{eff}}\left(\phi_{\text{obs}}\right)+L_{H}\left(H\left(\phi_{\text{obs}}\right)\right)$} is extremized, it yields classical, Lagrangian equations (like Einstein’s field equations, see Supplementary 3) in the appropriate large-limit *N* or large spin regime (see Supplementary 4 for a full derivation breakdown). Here, M_*obs*_ denotes the observer’s boundary, i.e., the portion of the spacetime boundary that corresponds to the observer’s interface; *d*^*d*^*x* this is the integration measure over the *d*-dimensional boundary. It represents the volume element on $\sqrt{-\gamma }$, here, γ is the determinant of the induced metric γ_*ab*_ on the boundary M_*obs*_. The factor −γ ensures that the integration measure is invariant under coordinate transformations on the boundary. *L*_eff_(*ϕ*_obs_), this term is the effective Lagrangian density for the observer’s degrees of freedom. The function *ϕ*_*obs*_ represents the observer’s state (which might include, for example, measurement outcomes, memory, or other internal variables). *L*_*eff*_ encapsulates the dynamics of these observer-centric processes that are Turing-computable. *L*_*H*_(*H*(*ϕ*_obs_)) represents the hypercomputational contribution to the boundary Lagrangian density. *H* is a function or process intrinsic to the observer that operates hypercomputationally, i.e., beyond the limits of standard Turing computation, and it acts on the observer’s state Φ_*obs*_. *L_H_* then quantifies the energetic or dynamical effect of this hypercomputational process on the boundary. *S*_*obs*_ is the total observer boundary action, obtained by integrating the sum of these two Lagrangian densities over the observer’s boundary. This action plays a crucial role in determining the emergent bulk geometry when combined with the bulk gravitational action. This observer-specific boundary action is fundamentally different from standard boundary terms in AdS/CFT because it explicitly depends on observer degrees of freedom, influencing how bulk fields evolve. These equations emerge as the saddle-point approximation of the full path integral, reflecting the Lagrangian laws we observe. Thus, the bulk obeys Lagrangian dynamics because they arise from a self-consistent extremization process that includes observer-centric constraints.

Likewise, in holographic scenarios of *N*-Frame (Edwards, 2023, 2024), the entropy of black hole horizons, for example, is related to the entanglement entropy of boundary degrees of freedom via the Ryu–Takayanagi formula. When observer-specific boundary information (including hypercomputational contributions) are incorporated, they influence the effective boundary action and thus the entanglement structure. The effective entropy *S*_*ent*_ can be expressed as $S_{e\,n\,t}\propto \frac{A\,r\,e\,a\,\left({\text{M}}_{o\,b\,s}\right)}{4\,G\,\hbar }$, where the area here is determined by the boundary conditions that include the observer’s *C*_*intO*_ computational and hypercomputational constraints. In a spin-network picture, the observer’s boundary states (e.g., specific intertwiners or spin labels) determine the microstates of the horizon, leading to a quantized area law for entropy.

By introducing a hypercomputational process *H* intrinsic to the observer *C*_*intO*_ (as stated in Postulate 10), the boundary information becomes (*b*,*h*) with *h* = *H*(*b*), whereby *b* is the Turing-computable component and *h* is the hypercomputational contribution. This augmented boundary state contributes to the effective action, and because *H*(*b*) is not Turing-computable, the overall mapping from boundary data to emergent geometry is enriched beyond standard algorithmic processes. However, even though the hypercomputational component makes the evolution of the system more complex and less predictable (i.e., computationally irreducible), the resulting effective action still yields classical, Lagrangian equations in the large-limit. In this way, the emergent laws of physics, including entropy relations and dynamical (Lagrangian) laws, are shaped by both the algorithmic and hypercomputational aspects of the observer’s *C*_*intO*_ interface. The extended *N*-Frame (Edwards, 2023, 2024) framework therefore explains why the universe obeys Lagrangian laws as a consequence of a variational principle that integrates both standard and hypercomputational observer-boundary effects. Simultaneously, it accounts for the observed entropy via the boundary’s entanglement structure, linking informational constraints to gravitational thermodynamics. This provides a self-consistent picture where emergent geometry, dynamics, and thermodynamics arise from the recursive, observer-centric interplay of quantum and hypercomputational processes. This derivation therefore provides compelling theoretical evidence that the approach is internally consistent and plausibly extends known frameworks such as holography and spin-foam models to include observer-centric, hypercomputational effects. It shows that, if one accepts the underlying assumptions, the emergent Lagrangian dynamics and entropy considerations derived from the variational principle with observer-boundary contributions provide a plausible explanation for how human consciousness might transcend algorithmic limitations and “identify truth” in Gödel incompleteness problems in a way that formal mathematical proof cannot capture.

The implications for AdS/CFT and quantum gravity are that in AdS/CFT, the boundary field theory dictates the bulk gravitational geometry via holography. If the boundary theory includes hypercomputational dynamics, then the bulk metric *g*_*μν*_ must encode non-algorithmic information. This suggests that the bulk spacetime is not merely a solution to classical differential equations but a structure containing irreducible noncomputable complexity. If spacetime geometry encodes non-Turing-computable information, this means that spacetime evolution is not fully describable by conventional physics. Quantum gravity theories based purely on computable quantum field theories would be incomplete since they fail to account for hypercomputational contributions from the observer *C*_*intO*_. This opens the possibility that spacetime emergence itself is an inherently hypercomputational process, meaning that some aspects of quantum gravity may never be fully simulated on a finite quantum computer. A particularly interesting consequence of this idea relates to holographic entanglement entropy. The Ryu-Takayanagi formula states that the entanglement entropy *S_A_* of a boundary region $S_{A}=\frac{\text{Area}\,\left(\gamma_{A}\right)}{4\,G_{N}}$, whereby γ_*A*_ is the minimal surface in the bulk. However, if the observer’s boundary action contains hypercomputational elements, then the boundary density matrix ρ_*A*_ may encode noncomputable correlations. The bulk minimal surface γ_*A*_ must adjust to match a boundary entropy structure that is itself non-algorithmic. This suggests that the bulk spacetime geometry itself is encoding non-Turing-computable entanglement information.

In addition to accounting for “identify truth” in Gödel incompleteness problems in a way that formal mathematical proof cannot capture, this approach might also explain why quantum measurement outcomes appear non-deterministic. The wavefunction collapse could correspond to a transition between different hypercomputable spacetime structures. Furthermore, if spacetime geometry itself encodes noncomputable information, this might provide a resolution to the black hole information paradox. The Hawking radiation state could contain hypercomputational correlations that are not fully retrievable by standard quantum mechanics. Finally, if some aspects of spacetime geometry are noncomputable, then no classical or quantum computer could fully simulate them. This suggests that AI models based solely on Turing computation may be fundamentally limited in understanding the full nature of reality and would be unable to be conscious in any way.

From the Turing-computable *L*_eff_(*ϕ*_obs_) aspects of the boundary *b*, the universe can be described in these informational terms, the conscious observer *C*_*intO*_ can also be described in these terms as well as their interactors with objects (information) around them. The *N*-Frame (Edwards, 2023, 2024) observer-centric realism posits that physical reality *P* can be interpreted largely in terms of observers exchanging finite, discretely encoded information with what they perceive. The key here (to prevent any perceived contradiction with hypercomputation) is to recognize that the finite, discretely encoded bits of information that conscious observers interact with represent the accessible layer of reality (the interface, the map, or the holographic screen), what’s measurable and processable given our thermodynamic and computational constraints. This interface (the map) is what we consciously experience and model using Turing-computable methods.

However, underlying this accessible layer may be hypercomputational processes, dynamics and structures that are non-Turing-computable (the territory). In other words, the deep, fundamental workings of spacetime might involve hypercomputational effects, but these effects get “filtered” or “compressed” into a finite, discrete form at the level of our conscious interfaces (e.g., through holographic screens or Markov blankets). Thus, while the observer-centric realism framework describes physical reality in terms of finite information exchange, it does not preclude the existence of hypercomputational dynamics in the underlying substrate. The finite bits are the end product of potentially hypercomputational processes that our conscious systems cannot directly access in their full non-computable complexity. So, here, rather than passively observing, each conscious internal observer *C*_*intO*_ (to the universe as a system) is an active, self-organizing system situated within an environment (the universe as a system and as part of this system self-referentially). The process of gathering or refining information from the world the observer *C*_*intO*_ (the observable world as in interface, i.e., the territory) exists within (and as part of) incurs a fundamental thermodynamic cost, in line with Boltzmann’s (1877) and Landauer’s (1961) ideas that entropy reduction requires energy. A “boundary,” such as a Markov blanket (this could represent a holographic screen in accordance with the holographic principle, or more simply a conscious user interface), mediates these interactions, ensuring that everything from sensory input to quantum measurements crosses a well-defined interface, and is broadly consistent with other work (Fields, 2016; Fields et al., 2021; Fields et al., 2022).

Within this *N*-Frame (Edwards, 2023, 2024) framework, a conscious internal observer *C*_*intO*_ (to the universe as a system) obtaining information about its surroundings within the interface equates to reducing uncertainty or entropy about some state of the environment. Boltzmann’s classical definition *S* = *k*_*B*_*ln*⁡Ω reminds us that decreasing Ω, the number of possible states compatible with the observer’s *C*_*intO*_ information requires an energetic expenditure. Landauer’s principle refines this to specify that erasing or irreversibly writing one bit of information at temperature *T* costs at least *ln*⁡(2)*k*_*B*_*T*. Whether the information is classical or quantum, no process can evade that fundamental limit at the level of the conscious user interface (the map, or the holographic screen that we use to interact with the deeper layer, the territory).

**FIGURE 5:**
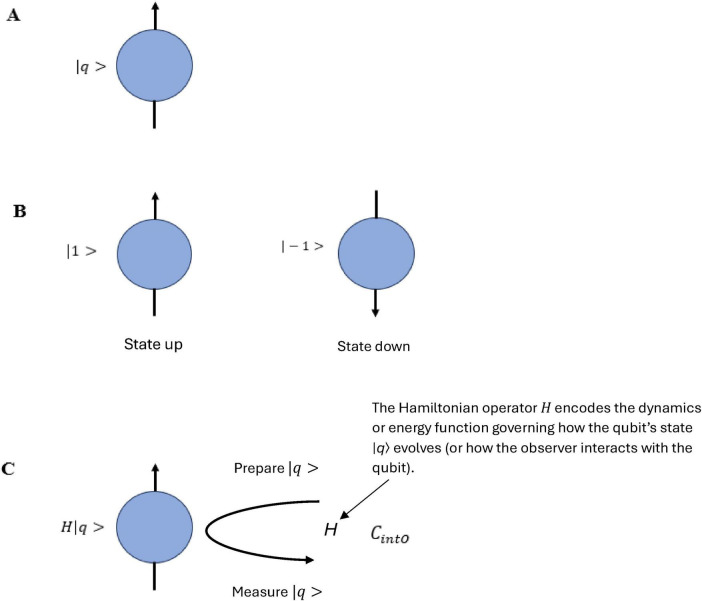
The informational thermodynamic cost for an observer agent *C*_*intO*_ to ask one question (making a measurement) about the external world. The information and energy cost of the universe asking a question about itself from its multiple *C*_*intO*_ observer perspectives. **(A)** represents a single qubit. **(B)** represents two-qubit states, up —1⟩ and down —–1⟩ (this can also be in a superposition, undecided state). **(C)** represents the conscious observer *C*_*intO*_ asking a question about the universe (taking a measurement).

An illustration of this can be given in quantum settings, whereby Planck’s constant ℏ sets the scale for discrete quanta of action *A=Et* (*A* = action, *E* = energy, and *t* = time). When the observer *C*_*intO*_ interacts with a qubit |q⟩ (see Figure 5A for a qubit representation, Figure 5B for two states of a qubit up and down but these can also be in a superposition of states also, and Figure 5C an observer asking a question about the qubit) effectively “asking” what state |q⟩ is in (making a measurement), there is a minimum action threshold and a corresponding energy cost. Specifically, if the observer aims to reduce its entropy by one bit (i.e., gaining one bit of information about the description of its environment within the interface) through this interrogation, Boltzmann (1877) suggests at least *ln*⁡(2)*k*_*B*_*T* of free energy is needed. More generally, we can write β ≥ *ln*⁡(2) to allow for practical inefficiencies, so the Hamiltonian guiding the question can be written as *H* = β (*k*_*B*_*T*) *M*, whereby *M* is a dimensionless operator (no intrinsic energy scale) that encodes or decodes a bit of information. Acting with *H* over a small interval d*t* transforms the qubit’s state, captured by *H*|q⟩ = β(*k*_*B*_*T*)*M*|q⟩ = E|q⟩, indicating an energy of β(*k*_*B*_*T*) is exchanged to accomplish this one-bit measurement or preparation. Notably, both the initial “preparation” of |*q*⟩ (e.g., writing a “1”) and the subsequent measurement of |*q*⟩ (reading that “1”) happen under this operator *M*. Symbolically, *M*:1,|*q*⟩→|1∠1,|*q*⟩, where the first arrow sets (prepares) |*q*⟩ into the up state, and the second arrow measures that state (the change in its state). When we talk about “setting” the qubit |*q*⟩ into an “up” state often denoted |1⟩, we are effectively choosing a specific basis or a specific reference state against which we pose our “yes/no” question. In quantum information theory, asking the qubit “Are you in the state |1⟩?” is a common way to define one bit of information. Preparing the qubit in |1⟩ sets the reference for that basis, so the yes/no question becomes, “Does the qubit remain in |1⟩ or not?” Quantum measurements must be made with respect to some observable such as the |1⟩ up state. If the “up” state |1⟩ is the eigenstate of the chosen observable, then initializing the qubit to |1⟩ and later measuring in the {|0⟩,|1⟩} basis makes it straightforward to interpret the outcome.

This confirms the idea that conscious information processing occurs through discrete, energy-limited exchanges at the level of the conscious interface. The discrete, energy-limited exchanges at the conscious interface represent the computable, measurable layer of information processing. However, this interface is just the accessible “output” of a deeper, underlying substrate. In our *N*-Frame model, while *L*_eff_(*ϕ*_obs_) captures the Turing-computable aspects (i.e., the finite bits of information processed at the boundary), the hypercomputational component *L*_*H*_(*H*(*ϕ*_obs_)) represents processes that go beyond algorithmic, energy-limited exchanges. While the conscious interface itself processes information in a discrete, energy-limited, Turing-computable manner, the overall capacity of the observer *C*_*intO*_ to grasp truths that are noncomputable is enabled by an underlying hypercomputational component [*L*_*H*_(*H*(*ϕ*_obs_)), they are not conscious of why they can do this as this is only accessible at the deeper hypercomputational layer and not the interface itself]. This deeper layer provides the extra capacity needed to identify or intuit truths that lie beyond formal mathematical proof, thereby offering a potential resolution to Penrose’s Gödel incompleteness puzzle.

**FIGURE 6:**
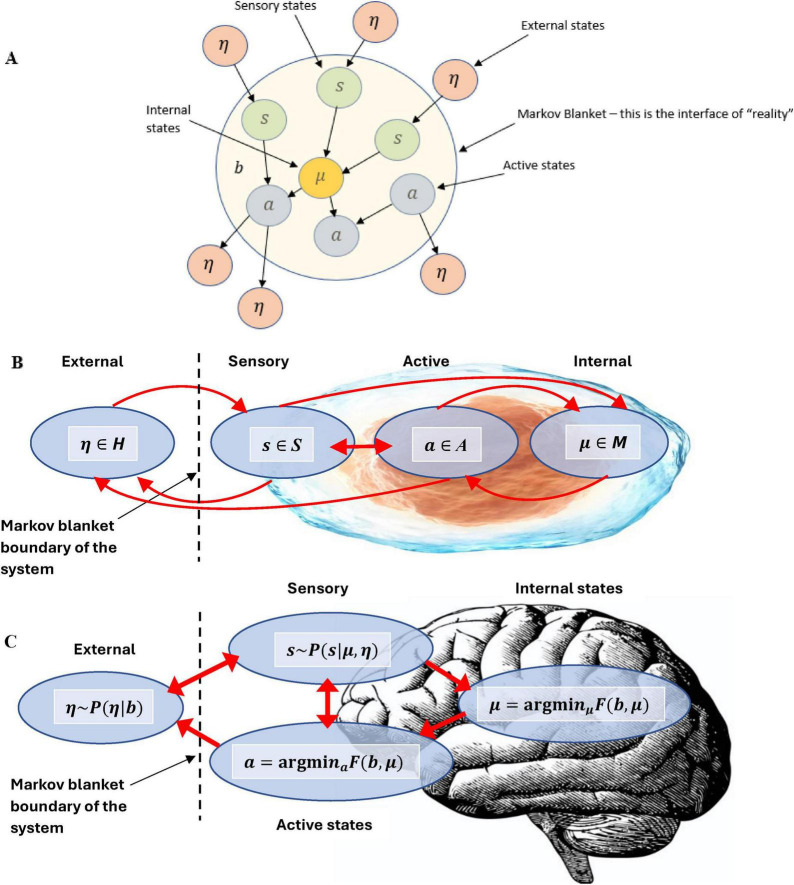
**(A)** illustrates a simple schematic representation of a Markov blanket containing sensory, internal, and active. **(B)** illustrates the Markov blanket of a cell whereby states can be thought of as a series of sets with a clear Markov boundary between internal (inner) states and external (outer) states. **(C)** illustrates the Markov blanket ensemble dynamics of internal, sensory, active, and external states of the brain and its environment.

**FIGURE 7:**
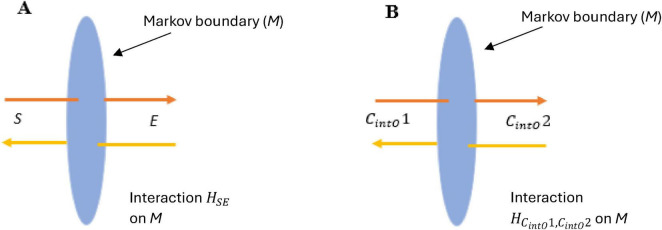
**(A)** An illustration of physics as information processing between a sub-system *S* such as an observer *C*_*intO*_ or the measuring device the observer uses to measure its environment *E*, where the transfer of information is across a finite state space Markovian boundary. **(B)** This also shows how two observers (*C*_*intO*_*s*) communicate across the finite state space Markovian boundary. This information processing requires energy and is finite hence requires data compression as an interface boundary, i.e., the 4D world we see rather than something deeper (higher dimensional) and more informationally expensive (e.g., 10+ interacting dimensions).

Because quantum dynamics is unitary, one can also represent the time evolution with *P*(*t*) = *e*^−*i/ℏHt*^*e*^−β*M*^, whereby the factor *e*^−β*M*^ incorporates the thermodynamic perspective, and a Wick rotation $\frac{i\,t}{\hbar }=\frac{1}{\left(k_{B}\,T\right)}$ links imaginary-time evolution to thermal processes. Viewed from a boundary standpoint (the boundary interface Markov blanket in active inference acting as holographic screen in quantum gravity; see Figure 6A), this “question-asking” Hamiltonian underscores the energy–information exchange that happens across an interface. The observer’s *C*_*intO*_ boundary (Markov blanket) or measurement apparatus (eyes, cell, cognition within a brain etc., see Figures 6B, C) effectively “probes” |*q*⟩ with minimal action ℏ. From an *N*-Frame perspective (Edwards, 2023, 2024) quantum information theory is therefore an observer-centric realism interpretation of physics that describes self-organizing systems that exchange finite discretely encoded information across some intervening boundary. Analogous principles apply in classical systems, whereby Shannon’s entropy formula quantifies informational “surprise,” yet we still incur *ln*⁡(2)*k*_*B*_*T* of energy per bit erased or stored. Across micro to macro scales, boundaries remain pivotal, as one can interpret AdS/CFT as a high-energy exemplar of how a boundary (the CFT on the AdS boundary) can encode all the information of the bulk, while in a biological or cognitive system, a Markov blanket physically and statistically separates an agent’s internal states from external ones. The AdS/CFT hologram effectively acts as a conscious interface of the observer to conserve information processing, as the 4D world we consciously observe as reality is a highly information compressed representation of something deeper (possibly hypercomputational in part *L*_*H*_(*H*(*ϕ*_obs_)), and thus save on thermodynamic energy of a finite system, i.e., the finite universe). See Figure 7A for an illustration of information processing between an observer *C*_*intO*_ and its environment *E*, whereby the transfer of information is across a finite state space Markovian boundary, and Figure 7B represents how two observers *C*_*intO*_*s* communicate across a finite state space Markovian boundary (such as two people communicating). This information processing requires energy, and as the boundary (the system in which the observers *C*_*intO*_*s* exist and are part of self-referentially) is a finite state space, its information is also finite, and its energy is finite, so this is why what we see as reality (the 4D world around us) is a highly compressed 4D conscious interface (it likely represents a maximizer for utility and function whilst minimizing information and thermodynamic cost). There is simply not enough information and energy (the thermodynamic cost is too high) for us to see the world (the system we exist in) in its real uncompressed form given the epistemic limit and computational boundary of observers like us that exist within and as part of the system (the system that the observers evolve from self-referentially places an upper bound on the information that the observers can process, and this limit is also smaller than the total finite information of the boundary of the system itself).

From this *N*-Frame (Edwards, 2023, 2024) description, we see that any act of measurement, down to the simplest possible “yes/no” interrogation of a qubit, entails a thermodynamic cost, a quantum of action, and a boundary-based exchange of bits or qubits. Every time a conscious internal observer *C*_*intO*_ (to the universe as a system) refines its model (its understanding of the system it exists in and as part of self-referentially, i.e., asking what is real), shifting from uncertainty to epistemic knowledge, it must pay a price in energy, consistent with Landauer’s principle. In doing so, the observer *C*_*intO*_ effectively transforms potential microstates Ω into a single realized outcome, whether via classical or quantum means. The boundary acting as a conscious interface at one level a Markovian blanket and at another a holographic screen represented as AdS/CFT, i.e., it encodes the information of the higher-dimensional “bulk” gravitational theory such as an AdS space with quantum fields. Here, the bulk is the deeper, higher-dimensional reality, while the boundary represents a compressed, information-rich interface marks the locus of that transformation, ensuring that information, energy, and entropy remain intimately linked and guiding the self-organization of the self-referential conscious internal observer *C*_*intO*_ (to the universe as a system).

## 3 A deeper dive into the holographic principle and *N*-Frame

**FIGURE 8:**
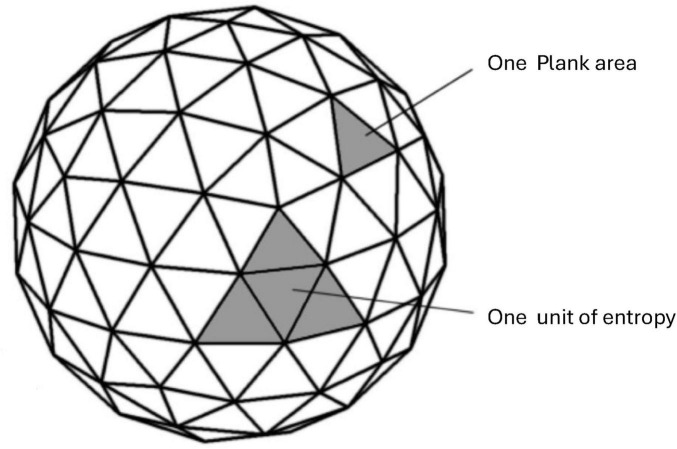
The Bekenstein-Hawking entropy of a black hole is measured in Planck areas, representing the Black hole event horizon. Source: Jacob D. Bekenstein with permission.

The boundary described by AdS/CFT as a hologram describes the conscious interface we see and emerges out of the holographic principle of theoretical physics. The “holographic principle” in theoretical physics emerged from studies of black hole thermodynamics in the 1970s and early 1980s, notably through the work of Jacob Bekenstein and Stephen Hawking. Bekenstein (1973) observed that a black hole’s event horizon area might represent its entropy, leading to the now famous Bekenstein–Hawking entropy formula $S_{B\,H}=\frac{k_{B}}{4}\,\frac{A}{l_{P}^{2}}$, whereby *S*_*BH*_ is the Bekenstein–Hawking entropy of a black hole (Bekenstein, 1973; Hawking, 1974, 1975). This quantity is proportional to the area of the black hole’s event horizon (see Figure 8). *k_B_* is the Boltzmann’s constant, a fundamental constant ≈1.380649×10^−23^ J/K relating temperature to energy. *A* is the area of the black hole’s event horizon. In standard international system of units, *A* has dimensions of *m*^2^. *l_P_* is the Plank length defined by $l_{P}=\sqrt{\frac{\hbar \,G}{c^{3}}}$, where ℏ is the reduced Planck’s constant, *G* is the gravitational constant, *c* is the speed of light, and *l*_*P*_≈1.616255×10^−35^*m*. This is relevant as the holographic principle as the Bekenstein–Hawking entropy shows that a black hole’s entropy is proportional to the area *A* of its horizon not its volume, a key insight that helped motivate the holographic principle. Hawking’s (1974; 1975) calculations that show black holes radiate thermally further cemented the idea that black holes have temperature and entropy, implying a deep connection between quantum mechanics, gravity, and thermodynamics.

Building on these insights, Hooft (1993) argued for the principle that physical processes within a volume of spacetime may be encoded on a lower-dimensional boundary, laying the conceptual groundwork for what Susskind (1995) referred to as the holographic world, via a holographic principle. The holographic principle is the notion that all the information contained within a region of spacetime could be described by degrees of freedom “written” on its lower-dimensional boundary. In other words, for certain quantum-gravitational systems, the maximal number of degrees of freedom scales with the area of a boundary surface (rather than with the volume). This principle was realized concretely by Maldacena (Maldacena, 1999; Maldacena, 1997) with the AdS/CFT correspondence (Anti-de Sitter/Conformal Field Theory duality), which asserts that a gravitational theory in a higher-dimensional (AdS) “bulk” is fully equivalent to a Conformal Field Theory defined on the bulk’s lower-dimensional boundary. Since then, AdS/CFT has been viewed as a precise mathematical realization of the holographic principle.

From an *N*-Frame perspective (Edwards, 2023, 2024) the boundary described in AdS/CFT as a holographic projection of the higher-dimensional system, and as formulated by the holographic principle, i.e., emphasizing that all bulk physics (including quantum states, gravitational degrees of freedom, etc.) is encoded in the AdS boundary, can be interpreted as a projected 4D conscious interface through which we access reality (i.e., the 4D universe that we, as conscious observers *C*_*intO*_, perceive around us). In that sense, the “holographic principle” becomes not just a formal statement about black hole entropy or string theory, but also a deeper framework for how information about the “the world” is captured and projected onto a lower-dimensional surface that we, as observers *C*_*intO*_*s*, perceive. Therefore, *N*-Frame (Edwards, 2023, 2024) provides an interpretative extension of the holographic principle, suggesting that the holographic boundary can be viewed as a sort of “projection” or “conscious interface” from a deeper (higher-dimensional) level of reality into the effective 4D world we experience. *N*-Frame’s (Edwards, 2023, 2024) ontological framework of an observer-centric realism posits that what we call “reality” is a “lower-dimensional representation” of more fundamental (bulk) degrees of freedom. From that vantage, the holographic principle becomes more than just a statement about black hole entropy or string theory, as it is expanded into a general principle describing how information of the world might be captured and then experienced by observers *C*_*intO*_*s*. This is *N*-Frame’s (Edwards, 2023, 2024) observer-centric realism interface principle.

This idea aligns with observer-centric and active-inference perspectives. For example, if every observer *C*_*intO*_ has a “boundary” (such as a Markov blanket in cognitive science) that mediates interactions with the external world, then the holographic principle in physics suggests a parallel structure, i.e., the degrees of freedom “inside” can be reflected or stored “on” some boundary “outside.” Broadly interpreted, the boundary in AdS/CFT and the notion of a conscious interface (Markov Blanket and holographic screen) can be seen as two sides of the same coin, both describing how finite, discretely encoded information about a complex or higher-dimensional domain is extracted, organized, and processed within a (seemingly) lower-dimensional or more “manageable” space. The *N*-Frame perspective suggests that the conscious interface functions like a Markov blanket at one level (separating an observer’s cognitive states from the external world) while also acting like a holographic screen in physics (compressing higher-dimensional reality into an information-limited observer experience). These two perspectives are two sides of the same coin, both describing how information is extracted, organized, and processed across a boundary.

**FIGURE 9:**
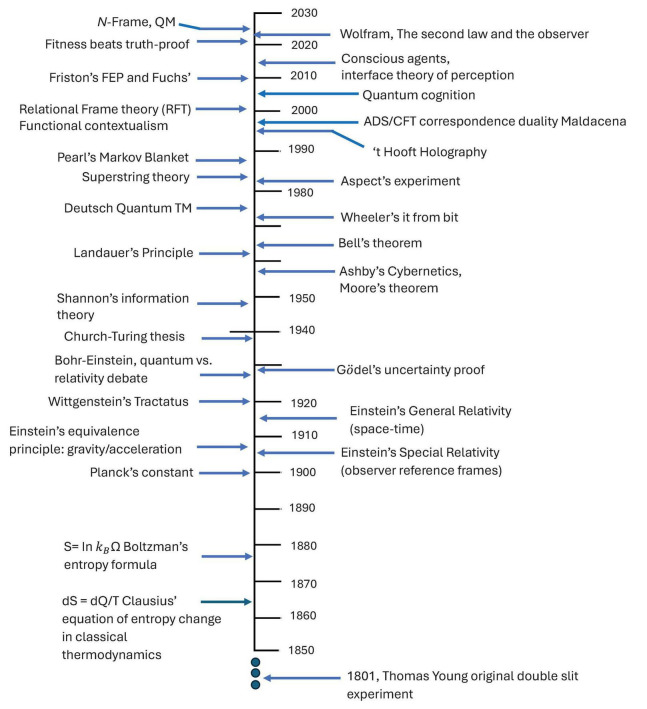
Historical progress from classical thermodynamics to quantum information, to evolutionary observer *C*_*intO*_ interface dynamics via *N*-Frame illustrated within a timeline.

Extending this idea even further, *N*-Frame (Edwards, 2023, 2024) proposes that the observer *C*_*intO*_ is, in a deeply self-referential sense, the universe observing itself through its own perspective consistent with Wheeler’s (Wheeler, 1992) “It from bit” dictum and other works (Faggin, 2019, 2021). This view supports the notion of a tri-aspect monist equivalence, represented as ψ→Φ≡*C*_*intO*_ ≡ *P* (see Figure 4), which extends Penrose’s (Penrose, 1994) triadic view of how the three “worlds” (or realms) interrelate. Here, the Physical *P* realm (the reality of matter and energy we observe), the mental realm *C*_*intO*_ (our conscious, subjective experiences and minds), and the Platonic realm ψ→Φ (the abstract, timeless domain of mathematical truths). Penrose suggests that each realm reflects the others in a subtle, self-referential way, implying that neither the mental nor the Platonic realm can be fully reduced to the physical alone. *N*-Frame (Edwards, 2023, 2024) formalizes this reflection as a formal tri-aspect monist equivalence principle ψ → Φ ≡ *C*_*intO*_ ≡ *P*, whereby the Platonic realm (ψ→Φ), the mental or phenomenological realm *C*_*intO*_, and the physical realm *P* reflects three manifestations of the same underlying “encoded” structure. In this tri-aspect view, ψ → Φ ≡ *C*_*intO*_ ≡ *P* effectively becomes a single unity of information and existence, such that the Platonic, mental, and physical realms are all facets of one encoded holographic reality projected as a conscious interface (the 4D world we as conscious observers *C*_*intO*_ observe and experience). Interpreted broadly, the “bulk space” of the hologram can be understood as the deeper substrate from which these different manifestations emerge, thereby making the boundary in AdS/CFT and the notion of a conscious interface two sides of the same coin. Both illustrate how finite, discretely encoded information about a complex or higher-dimensional domain is extracted, organized, and re-presented in a more “manageable” space, ultimately allowing the universe to “know itself” through observers like us *C*_*intO*_ embedded within it self-referentially (see Figure 9 for a timeline of the emergence of key ideas in entropy and physics the have led to the emergence of *N*-Frame which incorporates all of these prior ideas).

## 4 The computational boundary of the observer and the perception of personal time

**FIGURE 10:**
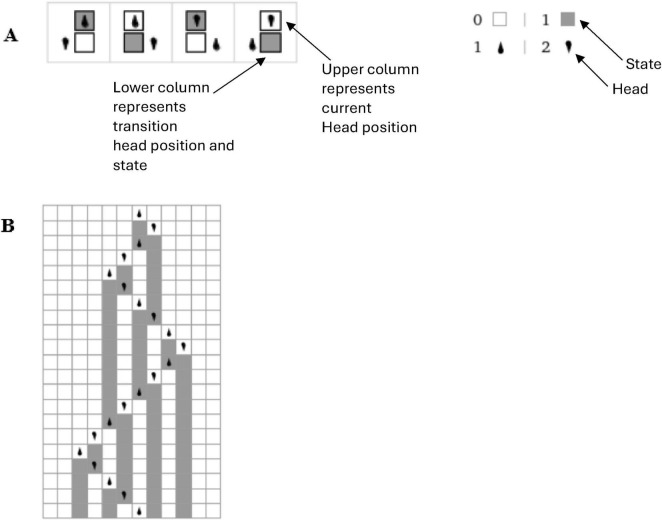
**(A)** Gives an illustration of a two-state, two-color Turing machine (rule 2506), with the black droplet icon representing the location and state of the head. **(B)** shows the evolution of the state and position over 20 steps in time when using the 2506 transition rule.

The conscious internal observer *C*_*intO*_ according to *N*-Frame (Edwards, 2023, 2024) is consistent with Stephen Wolfram’s (Wolfram, 2023) idea in his book *The second law: Resolving the mystery of the send law of thermodynamics*, that different types of observers have different computational constraints. Here, Wolfram provides a novel account of entropy within the second law of thermodynamics, whereby instead of describing an external system’s entropic state, entropy is described by Wolfram as an emergent property of the computational characteristics of internal conscious observer *C*_*intO*_. It is the computational boundary (constraint) of the observer *C*_*intO*_, and the mismatch of this with the computational irreducibility of the underlying system that lead the observers *C*_*intO*_ to experience the second law (an increase in entropy). The computational irreducibility stems from the idea (Wolfram, 2002; Wolfram, 2023) that many systems governed by simple rules can still produce behavior so complex that no shortcut or closed-form solution exists for predicting their future states. In other words, to find out what such a system will do after many computational steps, one often has no better method than to simulate every step of the system’s evolution. According to Wolfram (Wolfram, 2002; Wolfram, 2023) the universe is based on these computationally irreducible steps in something he calls the Ruliad whereby simple computational rules form into complex physics such as relativity mapped via hypergraphs. Figure 10A gives an illustration of a simple two-state, two-color Turing machine (rule 2506), with the black droplet icon representing the location and state of the head. Figure 10B shows the evolution of the state and position over 20 steps in time when using the 2506 transition rule, and it is easy to see that very complex patterns can emerge from extremely simple rules, as these patterns become increasingly complex, the only way to simulate them (and epistemically understand them) is for the observer *C*_*intO*_ to simply carry out each step. Entropy is, therefore, an observer *C*_*intO*_ epistemic phenomenon reflecting our inability to track or reverse the underlying microstates efficiently and is central to the computational capacity of the observer *C*_*intO*_. As Wolfram (Wolfram, 2020) suggests the universe as a system emerges from these simple rules, this may explain why observers *C*_*intO*_ perceive time, i.e., it is the computational steps carried out sequentially (i.e., time can be perceived as a sequence of discrete steps such as seconds, minutes, hours, days, etc.).

In this picture of time, time is experienced because even the most fundamental processes must be iterated sequentially, no observer *C*_*intO*_ can skip steps, so the universe’s “updates” appear one after another. This view within *N*-Frame (Edwards, 2023, 2024), is also broadly consistent with that of theoretical physicist Carr (2021) who postulates a 5-dimensional (5D) spacetime to suggest that time may have an additional dimension beyond the conventional 4-dimensional spacetime (3 spatial dimensions plus 1-time dimension) of Einstein’s relativity. This 5th dimension, which he calls “conscious time,” is proposed to account for the subjective experience of time, or the flow of time, which is not fully explained by the physical time dimension (the time used in physics to describe the evolution of events, i.e., the 4th dimension). In conventional 4D spacetime, time is treated as a single, linear dimension that is part of the block universe, a static model where past, present, and future coexist, and there’s no observer “flow” of time. However, our conscious experience of time involves the perception of a present moment and the flow or passage of time, which conventional 4D spacetime does not easily accommodate.

Carr’s idea (Carr, 2021) is that the 5th dimension might represent this conscious aspect of time, somewhat separate from the external, physical time (4D) described by the conventional laws of physics. This “conscious time” could be where subjective phenomenological experience resides, potentially linked to consciousness itself of the observer *C*_*intO*_. This would be expressed as the observer’s real-time computational updating of its epistemic knowledge as it asks questions about the system (the universe) it exists within [via thermodynamic costs for each question asked, expressed as minimum energy: *E*_*min*_ = *ln*⁡(2)*k*_*B*_*T*]. This might offer a bridge between our current understanding of physics and our inner phenomenological experience of reality as observers *C*_*intO*_. By adding this extra dimension, Carr aims (and *N*-Frame) to address the puzzle of how subjective consciousness relates to the physical world, suggesting that consciousness could have a deeper dimensional structure beyond what conventional physics describes. Conventional physics treats time as a dimension in spacetime, without a built-in process for the subjective experience of the “flow” of time, or why we perceive a present moment. *N*-Frame (Edwards, 2023, 2024), aligned with Carr’s perspective (Carr, 2021) suggests that subjective experience and the flow of time need to be incorporated into our fundamental models of reality, and introducing a second “conscious time” dimension might provide a framework for explaining this gap. This is consistent with the idea of computational irreducibility as each step in the universe’s evolution needs to be iterated sequentially, no observer *C*_*intO*_ can skip steps, so the universe’s “updates” appear one after another as a process of the observer *C*_*intO*_ updating its epistemic beliefs about the world. This is also consistent with the QBist (Fuchs, 2014, 2023) perspective of QM, which is centered on the observer *C*_*intO*_ updating its epistemic beliefs rather than describing some physical reality.

This description builds on the same observer-centric framework that we applied to the AdS/CFT boundary concept. In our prior *N*-Frame AdS/CFT description, the boundary (or holographic screen) serves as an interface where information about the higher-dimensional bulk is encoded and is then processed by a conscious observer *C*_*intO*_. Here, Carr’s idea of a “conscious time” dimension extends that concept by proposing that an additional (5th) dimension captures the subjective flow of time, the real-time sequential updating of an observer’s epistemic model of the world. In both cases, the focus is on how conscious observers interact with, and extract information from a deeper level of reality. While the AdS/CFT boundary captures the static mapping of bulk degrees of freedom onto a lower-dimensional surface, the introduction of “conscious time” emphasizes the dynamic, computationally irreducible process by which observers update their internal models. This dynamic updating is consistent with the idea that no observer *C*_*intO*_ can consciously skip computational steps (at least at the level of the Turning computational aspects of the observer *L*_eff_(*ϕ*_obs_), i.e., the phenomenological experiential side of it) mirroring the sequential nature of time as experienced subjectively. Thus, Carr’s proposal dovetails nicely with the earlier AdS/CFT description by adding another layer, the temporal or “conscious” dimension to account for the dynamic sequential and continuous phenomenological experience of time. Both perspectives (unified within *N*-Frame) underscore that our conscious experience is deeply tied to how we process and update finite, discretely encoded information at a boundary interface. For perception, this discrete updating is 10–30 Hz, or 10 to 30 times per second (VanRullen, 2016).

A geometric construction of this 5D conscious observer-time *C*_*intO_τ_*_, can be given by starting with the idea of a 4D block universe, represented by a manifold *M_4_* that has coordinates *x*^1^,*x*^2^,*x*^3^,*t*. This setup encapsulates the traditional spacetime view in relativity, whereby past, present, and future events exist in a static block. An observer’s worldline in this 4D manifold is a one-dimensional submanifold, or curve, γ(*t*), which parametrizes that observer’s $C_{i\,n\,t\,O}^{\prime }\,s$ path through spacetime. Although the parameter *t* might be interpreted as physical or proper time, it does not itself provide an objective observer *C*_*intO*_ “flow” of time, rather, it simply locates events along a continuous trajectory. To introduce a higher-level structure that could accommodate subjective time or consciousness, we extend this four-dimensional block to a five-dimensional manifold *M_5_* by adding a coordinate τ such that *M*_5_ = *x*^1^,*x*^2^,*x*^3^,*t*,τ.

This extra coordinate is interpreted as conscious observer time *C*_*intO_τ_*_, and is not directly measured by standard clocks or instruments. A natural projection π:*M*_5_→*M*_4_ sends each point in the 5D manifold to a corresponding event in 4D spacetime (the observer’s *C*_*intO*_ interface), effectively forgetting the τ-component (the τ-component is therefore not directly visible in conventional physics such as clocks). In this extended manifold, the observer’s full trajectory is a 5D curve Γ(τ) = [*x*^1^(τ),*x*^2^(τ)*x*^3^(τ),*t*(τ)τ], so that as τ changes, we track not only the observer’s position in 4D but also the evolution of their subjective time. A central feature of this construction is the possibility of branching futures. In the 4D block view, future events are already fixed, but the introduction of τ permits a branching structure in the higher-dimensional space, allowing for the observer *C*_*intO*_ to make free will choices at each branch thus *N*-Frame’s accounting for free will (collapsing many possible worlds into a singular free will chosen world at each branch). For each value of τ, there could be multiple potential future worldlines in 4D, each corresponding to a different outcome or decision. These different continuations form a family of possible paths and an observer’s conscious perception of time *C*_*intO_τ_*_ can be associated with selecting or following exactly one branch among many. In more formal terms, one can define a branching locus whereby multiple paths diverge at the same τ -value, and an internal selection function σ(τ) (this is the selection function for free will) that chooses which branch to instantiate at each step in τ. Thus, the observer’s *C*_*intO_τ_*_ subjective flow of time can be modeled by a single 5D curve that navigates these branching possibilities, even though all potential futures coexist within the higher-dimensional manifold. In this picture, conscious observer time *C*_*intO_τ_*_, τ is not a coordinate we can measure physically, but rather an internal or conscious phenomenological parameter. The block universe in *M_4_* remains static, while the observer experiences an apparent flow of time by moving through τ in *M_5_*. Any increment in τ corresponds to a step in which the observer’s $C_{i\,n\,t\,O}^{\prime }\,s$ consciousness identifies a new present moment within the set of possible future states. Because these branching worldlines in 5D incorporate varied outcomes or events, the conscious agent *C*_*intO*_ perceives decision-making or probabilistic changes over time.

**FIGURE 11:**
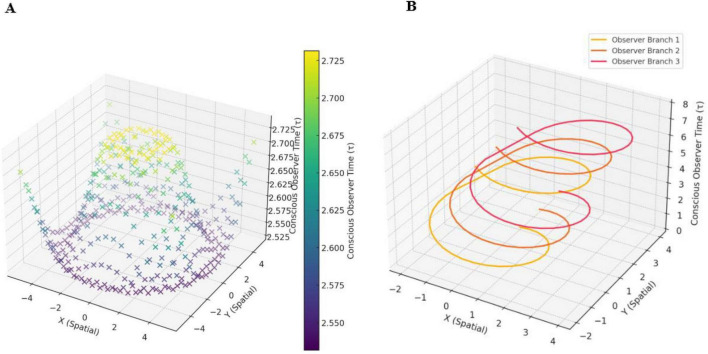
**(A)** Each point in the plot corresponds to an observer at a specific spatial position (*X*, *Y*) at a single physical time (*t*). The *Z*-axis is conscious observer time (τ), meaning how much subjective time has passed for each observer relative to their physical location. Some observers have experienced more subjective time passing than others (e.g., those at higher τ values). **(B)** Three possible observer trajectories in 3D Space. Each line represents an observer’s path through conscious observer time (τ) as they progress in physical time (t). The *X* and *Y* axes show spatial motion, while the *Z*-axis (τ) represents conscious observer time.

In a geometric sense, the 5D manifold can be viewed as a fiber bundle, with τ serving as a base coordinate and each fiber (above a particular τ-value) capturing the different 4D paths available. See Figure 11A^2^ for an illustration of this 5D manifold represented in 3D space (this is given as a 3D simplified and compressed plot to help us understand the 5D structure, because a direct visualization of a true 5D space is difficult for human intuition to understand, as the very nature of our conscious interfaces show we are adapted to perceive information in 3D rather than 5D geometry). Each point in the plot corresponds to an individual observer (many observers are plotted) at a specific spatial position (*X*,*Y*) at a single physical time (*t*). The *Z*-axis is conscious observer time (τ), meaning how much subjective time has passed for each observer relative to their physical location. Some observers have experienced more subjective time passing than others (e.g., those at higher τ values). The conscious observer *C*_*intO*_ exists at the boundary (like an AdS/CFT screen and not inside the bulk of the universe) where they process finite, discrete bits of information. Different locations in physical space correspond to different τ values, indicating that subjective conscious time is not necessarily uniform across observers, therefore conscious time is relational, deictic and entirely functionally contextual, consistent with *N*-Frame (Edwards, 2023, 2024) and RFT (Barnes-Holmes and Harte, 2022; Blackledge, 2003; Edwards, 2021; Edwards et al., 2022; Edwards et al., 2017; Hayes et al., 2001; Hughes and Barnes-Holmes, 2015; Torneke, 2010). From this perspective, as conscious time τ is entirely functionally contextual, this means that its value and flow depend on the observer’s contextual interactions, and position within the system (the universe). This suggests that the experience of time is shaped both by physical time and by contextual spatial positioning within the conscious interface. Figure 11B^3^ illustrates three possible observer trajectories in 3D Space. Each line represents an observer’s path through conscious observer time (τ) as they progress in physical time (t). The *X* and *Y* axes show spatial motion, while the *Z*-axis (τ) represents conscious observer time.

In order to describe a specific metric for τ, we first need to highlight a well-established connection between the strength of gravity (as encapsulated by the gravitational constant *G*) and the entropy (often interpreted as the information or computational capacity) of a black hole. According to black hole thermodynamics, the entropy of a non-rotating, uncharged (Schwarzschild) black hole is given by the Bekenstein–Hawking formula (Bekenstein, 1973; Hawking, 1975): $S=\frac{k_{B}\,c^{3}\,A}{4\,G\,\hbar }$ whereby *S* is the entropy of the black hole, *k_B_* is the Boltzmann’s constant, which relates entropy to temperature, *c* is the speed of light, *A* is the area of the blockhole’s event horizon (its outer surface) (see Figure 8), *G* is the gravitational constant, and ℏ is the reduced Plank’s constant for an illustration of the Bekenstein-Hawking entropy of a black hole is measured in Planck areas, representing the Black hole event horizon. The black hole’s entropy (its informational capacity or boundary) is directly proportional to the area *A* (its event horizon), which is clearly influenced by the strength of *G* in the equation. For a Schwarzschild black hole of mass *M*, then event horizon *R_s_* is given by $R_{s}=\frac{2\,G\,M}{c^{2}}$, thus, the horizon area is $A=4\,\pi \,R_{s}^{2}=16\,\pi \,\left(\frac{G^{2}\,M^{2}}{c^{4}}\right)$. As area *A* is influenced by gravity *G* then so is entropy *S*, and this can be shown when we substitute the area into the entropy formula (i.e., make entropy the focus of the equation), $S=\frac{k_{B}\,c^{3}\,\left(16\,\pi \,G^{2}\,M^{2}/c^{4}\right)}{4\,G\,\hbar }=k_{B}\,\frac{4\,\pi \,G\,M^{2}}{\hbar \,\text{c}}$. As shown, for a fixed mass *M*, the entropy *S* grows with *G*. Increasing the gravitational coupling leads to a larger event horizon for the same mass, hence a greater entropy and therefore information capacity or boundary.

Black hole entropy is frequently understood as a measure of the black hole’s information storage capacity (the number of microstates consistent with its macroscopic parameters). Under the holographic principle (Hooft, 1993; Susskind, 1995), a black hole’s entropy (Bekenstein, 1973; Hawking, 1975) represents a limit (the upper limit) on how much information or computational complexity can be encoded within a given region of space and therefore the information capacity of the observer’s *C*_*intO*_ conscious interface (see Table 1 for the various physics interpretations of gravity). If one interprets entropy as a form of computational capacity or boundary, then the strength of gravity, through *G*, sets how large the black hole’s horizon is for a given mass and thus how much information it can store. The stronger the gravitational coupling (the larger *G*), the bigger the horizon for the same mass and therefore the greater the entropy.

**TABLE 1:** Physics are mental models of mind, they are physical models through time of how our minds model some external world (a case example of explaining matter, time, and gravity).

Physics model	What is matter?	What is time?	What is gravity?
Classical theory (Newton—1687)	Matter is solid and indivisible entities. Matter is composed of particles with mass that occupy space and have a well-defined position and velocity. It follows deterministic laws of motion and interacts through forces like gravity.	Absolute and linear. Time is a universal constant, flowing uniformly for all observers, independent of the physical processes occurring in space. It is not affected by matter or energy and progresses in a single direction from past to future.	A force that acts at a distance between two masses. Newtonian gravity is described by the inverse-square law, where the gravitational force between two objects is proportional to the product of their masses and inversely proportional to the square of the distance between them. Gravity is an inherent property of matter, pulling objects toward each other.
Atomic theory (John Dalton—1803, JJ. Thomson—1887)	Matter is composed of atoms, which are made up of subatomic particles (protons, neutrons, and electrons). Most of an atom’s volume is empty space, with a dense nucleus at the center containing protons and neutrons, and electrons orbiting around this nucleus in defined probability regions. Matter is not solid but consists of these discrete, interacting particles.	Time is absolute in classical atomic theory, with atomic interactions occurring in a fixed, universal time frame. In the context of atomic physics, time governs the motion of electrons around the nucleus and the decay of radioactive substances, though this interpretation does not yet account for relativistic or quantum effects.	Gravity, in the context of atomic theory, plays an insignificant role at the atomic scale. The forces that dominate are the electromagnetic force (binding electrons to the nucleus) and the strong nuclear force (holding protons and neutrons together in the nucleus). Gravity becomes relevant only at much larger scales, as it is much weaker than these atomic forces. Atomic theory largely treats gravity as negligible when discussing subatomic particles.
Special relativity—The first observer-centric model (Einstein—1905)	Matter and energy are interchangeable, described by the equation *E* = *mc*^2^. Matter is a form of energy that manifests as particles with mass at rest, but as an object moves closer to the speed of light, its energy increases, and its effective mass increases. Matter does not exist independently of energy but is a form of it.	Relative and not absolute. Time is experienced differently depending on the observer’s state of motion. In special relativity, time slows down as an object approaches the speed of light, an effect known as time dilation. This means that time is not a universal constant but is intertwined with the speed of the observer, making time relative to the observer’s frame of reference.	Not explicitly explained in special relativity (this is covered by general relativity). However, the theory sets the stage for understanding how the concepts of space and time are affected by high velocities and energy.
General relativity (Einstein—1915)	Matter influences the curvature of space-time. In general relativity, matter and energy tell space-time how to curve, and that curvature tells matter how to move. Matter is no longer seen as an isolated entity moving through space but as part of a dynamic interplay with the structure of space-time itself.	Time is part of the four-dimensional space-time fabric. It is relative, not only dependent on the motion of the observer (as in special relativity) but also on the presence of mass and energy. Time runs more slowly in stronger gravitational fields, an effect called gravitational time dilation. The closer you are to a massive object, the slower time passes relative to a distant observer.	Gravity is not a force, as it was in Newtonian physics, but the curvature of space-time caused by mass and energy. Massive objects like planets and stars bend the fabric of space-time, and this curvature dictates how objects move, including the paths of light and free-falling bodies. This is famously represented in Einstein’s field equations, which describe how matter and energy warp the geometry of space-time, creating the phenomenon we perceive as gravity.
Quantum theory (Max Plank—1900, Einstein—1905 photoelectric effect, Niels Bohr—1913, Werner Heisenberg—1925).	In quantum theory, matter is described by wave functions rather than solid particles. Matter has both particle-like and wave-like properties, an idea known as wave-particle duality. The precise location of particles, such as electrons, cannot be known with certainty but can be described probabilistically, represented by a wave function that gives the likelihood of finding a particle in a particular place at a given time. Matter is no longer seen as discrete objects with definite boundaries but as quantum entities existing in superpositions of states until observed.	In quantum theory, time is usually treated as an external parameter that progresses uniformly, similar to classical theories. However, within quantum systems, time can behave differently at microscopic scales. In some interpretations, quantum events don’t unfold in a continuous flow but can exhibit time-symmetric behavior, where cause and effect are not strictly linear. In quantum mechanics, time is still absolute in the sense that it flows independently, but this is challenged in theories like quantum gravity.	Quantum theory does not provide a complete theory of gravity. Unlike forces such as electromagnetism, which are well-explained by quantum mechanics, gravity is not integrated into the quantum framework. Gravity remains a macroscopic force and attempts to unify it with quantum mechanics are still incomplete, as seen in approaches like quantum gravity or string theory. At atomic and subatomic scales, the effects of gravity are insignificant compared to quantum forces like electromagnetism and the strong and weak nuclear forces.
Quantum gravity	In quantum gravity, matter, space, and time are deeply interconnected, transcending the classical idea of distinct, independent entities. Matter is no longer viewed as a set of particles or wave functions existing in a pre-defined space-time. Instead, it is an emergent phenomenon from more fundamental quantum states of space-time itself. Matter arises from the quantum behavior of space-time, suggesting that it is part of a unified, underlying quantum field or network.	Time, like matter, is not treated as a fixed, independent entity. In quantum gravity, time may be emergent, meaning it arises from deeper, timeless quantum structures. The concept of time as a smooth, continuous flow breaks down at the Planck scale (the smallest scale in the universe). Time could behave differently at quantum scales, potentially being quantized or relational, where the progression of time is linked to changes in quantum states rather than a universal clock ticking uniformly.	Gravity is no longer a force or a curvature of space-time but is itself a quantum phenomenon. Quantum gravity seeks to describe gravity using the principles of quantum mechanics, aiming to unify general relativity (which describes gravity at large scales) with quantum mechanics (which governs the behavior of matter at the smallest scales). Various approaches, such as loop quantum gravity and string theory, propose that gravity emerges from the quantum properties of space-time, with space-time being made up of discrete, quantized units rather than a smooth continuum. Gravity at the quantum level is seen as a result of interactions between these quantum units of space-time.
Higher dimensions	In theories involving higher dimensions, such as string theory and brane cosmology, matter is not confined to the familiar three-dimensional space. Matter is seen as existing within higher-dimensional spaces, with the particles we observe (like electrons and quarks) being manifestations or “slices” of extended objects (such as strings or branes) in these higher dimensions. Matter in our 3D space could be just a projection or a lower-dimensional aspect of more complex structures that exist in higher-dimensional spaces.	Time, in higher-dimensional theories, may also extend beyond the single temporal dimension we experience. Some models suggest multiple time dimensions or that time itself could behave differently when considered in the context of higher-dimensional space-time. However, in many such theories, time remains a single dimension within a broader framework of spatial dimensions, though its behavior can be influenced by the existence of these higher dimensions. In scenarios like string theory, time and space are treated together as part of a multi-dimensional “space-time fabric” that includes more than the familiar four dimensions.	In higher-dimensional models, gravity is thought to propagate through all the dimensions, not just the familiar three spatial ones. One key idea is that gravity is much weaker than other forces because it “leaks” into these extra dimensions. This could explain why gravity seems so weak compared to other fundamental forces, like electromagnetism. In brane-world models, for instance, our universe is considered a 3D “brane” embedded in a higher-dimensional space (a “bulk”), and gravity, unlike other forces, can extend into the extra dimensions, which might account for the behavior of gravity at both cosmic and quantum scales.
Observer-centric universe: *N*-Frame—The universe as a mental model. The second observer-centric model. (Edwards, 2024)	At the 4D level, matter emerges from the interaction between quantum states and observer-induced collapse, defined by an observer’s frame of reference. At a higher-dimensional level, matter is a projection from an underlying holographic structure, contingent on the observer’s conscious interaction with reality. Within the AdS/CFT holographic model, *N*-Frame posits that the conscious observer serves as the boundary condition, selecting stable eigenforms of matter from a space of quantum potentialities. Matter is thus an emergent low-free-energy state, dynamically shaped by self-referential observation, quantum cognition, and informational constraints.	Time is a multidimensional, observer-dependent phenomenon that emerges from cognitive processes, quantum selection, and predictive coding. At the 4D level, proper time follows relativistic constraints when multiple observers interact within the interface. At the 5D level, time includes a conscious dimension, where branching worldlines exist before the observer collapses them into a single experienced reality (or selects a line so that the other lines (worlds) are no longer accessible, consistent with many world hypothesis). Time is not separable from cognition (observer experience), as it is actively structured by decision-making processes that minimize free energy and optimize survival-based predictive coding. The observer’s conscious state fundamentally shapes temporal structure, making time an emergent, self-referential feature of the universe rather than an independent background parameter.	At the level of the conscious interface, gravity emerges as an informational constraint, self-referentially shaped by the observer’s active participation in defining their experienced reality. Rather than a classical force, gravity is an emergent consequence of interactions across higher-dimensional informational structures, where the observer’s engagement modifies the fabric of space-time itself. The strength of gravity is proportional to the informational boundedness (entropy) of the bulk space, reflecting the holographic principle. This suggests that space-time, and the gravitational dynamics that structure it, are fundamentally encoded within a self-referential, observer-dependent universe, where consciousness plays an active role in selecting and stabilizing its own reality through quantum probability constraints and free-energy minimization.

Given this, we can now define a specific metric for τ via the observer’s *C*_*intO*_ computational boundary as a metric, consistent with the description given, a 5-dimensional manifold *M_5_* *x*^1^,*x*^2^,*x*^3^,*t*,τ we can postulate a metric *G*_*AB*_ (this is the 5D metric tensor) in block-diagonal form: *ds*^2^ = *g*_μ,ν_(*x*^α^)*dx*^μ^*dx*^ν^ + Ω^2^(*x*^α^)*d*τ^2^, whereby indices μ,ν = 0,1,2,3 run over the 4D spacetime coordinates Ω(*x*^α^) is a scalar coupling function. In the simplest case, Ω≡1, making τ act as a straightforward additional dimension. Depending on the chosen sign convention (+ + + + +) or (−−−−−), τ can be treated either as a spacelike or time-like coordinate, but here we assume it is distinct from the usual time coordinate *x*^0^.

An observer *C*_*intO*_, modeled as a curve in *M_5_*, can follow a parametric trajectory *X^A^*(λ) = [*x*^0^(λ),*x*^1^(λ),*x*^2^(λ),*x*^3^(λ),τ(λ)], whereby λ is an affine parameter (not necessarily equal to τ). The dynamics of this worldline in 5D can be obtained by minimizing the action $S=\int d\lambda \,\frac{1}{2}\,G_{A\,B}\,\frac{d\,X^{A}}{d\,\lambda }\,\frac{d\,X^{B}}{d\,\lambda }$ . The associated geodesic equation is then $\frac{d^{2}\,X^{A}}{d\,\lambda^{2}}+\Gamma_{B\,C}^{A}\,\frac{d\,X^{B}}{d\,\lambda }\,\frac{d\,X^{C}}{d\,\lambda }=0$, whereby $\Gamma_{B\,C}^{A}$ are the Christoffel symbols derived from *G*_*AB*_. Due to the block-diagonal nature of *G*_*AB*_, τ(λ)and *x*^μ^(λ) become coupled if Ω depends on the 4D coordinates *x*^μ^ . In that case, variations in *x*^μ^ can alter how τ evolves and vice versa through the geodesic condition. If Ω is constant, τ(λ) may decouple and evolve linearly in λ, resulting in a simpler (though still 5D) geometry. This can also be treated as a scalar field by embedding this into a field-theoretic framework. In this way we can treat τ(*x*^μ^) as a scalar field defined over the 4D manifold *M_4_*. In that scenario, one writes an action $S_{4\,D}=\int d^{4}\,x\,\sqrt{-g}\,\left[R+\alpha \,g_{\mu }^{\mu \,\nu }\,\tau_{\text{v}\,\tau }\,\tau -V\,\left(\tau \right)\right]$, whereby *R* is the scalar curvature of the 4D spacetime, α is a coupling constant, and *V*(τ) is a potential. Varying with respect to τ yields a Klein–Gordon-like equation: $\alpha \,\nabla^{\mu }\nabla_{\mu }\tau -\frac{\text{V}}{\tau }=0$, giving τ nontrivial dynamics in 4D.

To link this 5D geometry (or 4D scalar field) to an observer’s *C*_*intO*_ computational boundary, one could regard the observer’s interface e.g., a “holographic screen” or “Markov blanket” as a manifold with its own metric *g*_*ij*_. The distance in that metric might encode the computational or thermodynamic cost for transitioning between states on the boundary. A field equation on the boundary could enforce constraints analogous to Landauer’s principle or free-energy minimization, describing how the observer’s *C*_*intO*_ internal states adjust upon receiving information from the environment. Under active-inference or observer-centric interpretations, the observer’s $C_{i\,n\,t\,O}^{\prime }\,s$ movement through τ would represent the ongoing update of its internal states (knowledge) in time. The geodesic condition in 5D, or the evolution of a τ-field in 4D, would then reflect how the observer’s conscious experience or computational constraints co-evolve with external conditions. In short, the 5D metric *G*_*AB*_ endows τ with geometric significance, allowing distances or intervals along τ to be interpreted as the progression of conscious time. A field equation (e.g., $\nabla^{\mu }\nabla_{\mu }\tau -\frac{\text{V}}{\tau }=0$) invests τ with dynamics, so that it can respond to or guide the system’s evolution. Coupling to the observer’s $C_{i\,n\,t\,O}^{\prime }\,s$ boundary can embed additional constraints capturing finite computational resources, measurement resolution, and thermodynamic costs, consistent with an information-theoretic or holographic viewpoint on cognition and perception. This, thereby, offers a blueprint for discussing “conscious time” in geometric, dynamical terms. It is also possible to embed this extra “conscious dimension” into a unified field-theoretic framework, reminiscent of how Einstein unified metric and field (in his general relativity theory) by treating the spacetime metric itself as the gravitational field (Einstein, 1916; see Supplementary 5 for further details).

**FIGURE 12:**
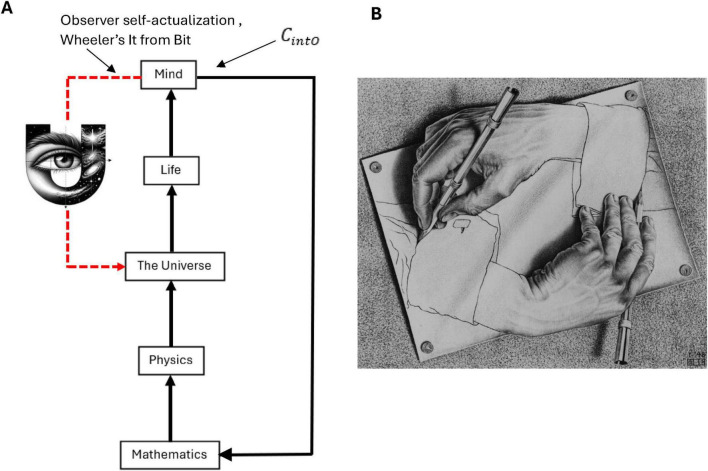
**(A)** Illustrates a closed cycle (strange loop) of how mathematics underlies physics, physics underlies our description of the universe, the universe gives rise to life, life gives rise to minds, our minds give rise to mathematics (symbolic communication), and therefore a self-referential observer *C*_*intO*_ cycle emerges Ψ → Φ ≡ *C*_*intO*_ ≡ *P*. **(B)** Escher canvas painting itself self-referentially. Printed here with permission. M.C. Escher’s “Drawing Hands”^©^ 2024 The M.C. Escher Company-The Netherlands. All rights reserved (www.mcescher.com).

Consistent with *N*-Frame, Carr (Carr, 2021) also points out that our physics models are actually mental models of the conscious mind. See Figure 12A which illustrates this self-referential nature of the conscious mind, the platonic world (mathematics), and the physical world they describe, which are self-referential and equivalent in line with the tri-aspect monist equivalence suggested by *N*-Frame (Edwards, 2023, 2024) ψ → Φ ≡ *C*_*intO*_ ≡ *P*. Here, a closed cycle (strange loop) displays how mathematics underlies physics, physics underlies our description of the universe, the universe gives rise to life, life gives rise to minds, our minds give rise to mathematics (symbolic communication), and therefore a self-referential observer *C*_*intO*_ cycle emerges Ψ → Φ ≡ *C*_*intO*_ ≡ *P*. This is also reflected by Escher’s drawing hands (see Figure 12B) which depicts two hands drawing each other on a canvas in a paradoxical self-referential loop synonymous perhaps with the way conscious mind, mathematics, and the physical world each reflect the other self-referentially. By embedding the observer’s *C*_*intO*_ minds perspective in the model (for instance, letting τ be an internal parameter representing the observer’s subjective flow), we highlight the feedback loop, i.e., mind emerges from the universe, but the universe as we know it, is a construct of the mind via our mathematical models.

**FIGURE 13:**
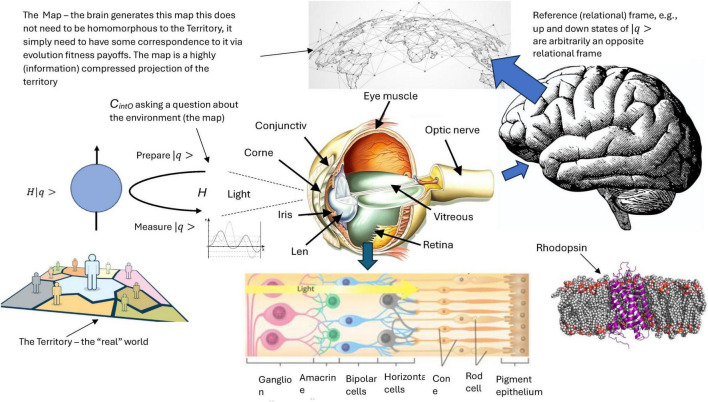
The map is not the territory (the system). The informationally bounded observer *C*_*intO*_ asks a question about the world (or territory/system) it exists in to update its model (map) of the system, and this exacts a thermodynamic cost of energy and information.

This point can also be further made explicitly, as via the predictive coding model of neuroscience, the brain generates a predictive model of the world (using Markov blankets, see Figure 6C). Here the brain generates a map about the world (a predictive model of the world or system it exists in) that does not necessarily homomorphically represent the real world (the territory as a system the observer exists in and is part of self-referentially), i.e., the map is not the territory (see Figure 13). Here, the observer *C*_*intO*_ asks a question about the system it exists in, and this exhibits a thermodynamics cost for each question it asks about the system (the universe) so there is a direct cost for the observer *C*_*intO*_ to update their predictive Markovian model (the map) about the system they exist in (the territory or the universe). *N*-Frame (Edwards, 2023, 2024) suggests that as organisms are bounded computationally which constrains their epistemic access about the system they exist in, this can be thought of as a cognitive light cone similar to as suggested by other work (Levin, 2019) which restricts predictive ability about future events and memory of past events for instance. This is also linked to Wolfram’s (2023) computational limits of the observer *C*_*intO*_ discussion, that entropy a facet of the observer’s computational limit which restricts their epistemic access about the world as its computationally irreducible in its emergent complexity.

**FIGURE 14:**
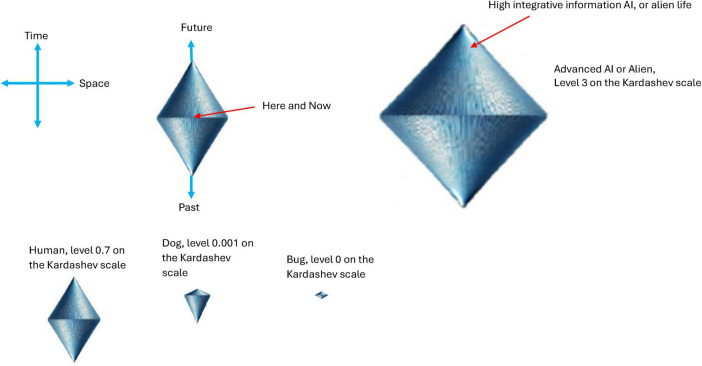
Arbitrary cognitive individuals can be classified by their computational boundary. The shape of the boundary defines each individual agent’s cognitive light cone. The size and shape of the light cones define the cognitive boundary of the agent and determine the scale of it goal-directedness, as well as its epistemic limit on a bounded interface of ADS/CFT holographic boundary.

Organisms with a much larger light cone (a large computational boundary) may have a more thermodynamically expensive conscious interface. For example, advanced AI or an advanced alien civilization perhaps at a level three on the Kardashev (1980) scale (with the ability of the civilization to control all the energy of a galaxy, such as all of the suns’ energy via Dyson spheres at galactic scale engineering) or a level four (the ability to control all the energy in the universe such as zero-point energy, dark energy, Hawking radiation of blackholes, quasars etc., at universal scale engineering) (see Figure 14) would have very large computational boundaries (illustrated as large cognitive light cones) and could include conceptualized Matrioshka megastructure brains or minds [first proposed by Robert J. Bradbury in the book *Year million: Science at the far edge of knowledge* (Broderick, 2008)]. Perhaps as these type 3 and 4 level megastructure observers *C*_*intO*_ could access greater energy, they may have greater resources to artificially supply their conscious interfaces. Their conscious interfaces could consequentially be more thermodynamically expensive whereby they could perceive the world (reality) in 10 or 11 dimensions rather than our four dimensions or five (if you count conscious time) dimensions that we are able to perceive due to our lower computational boundary. This draws parallels to how beings living in a lower-dimensional 2D “Flatland” [explored in the 19th-century classic science fiction novel *Flatland: A Romance of Many Dimensions* (Abbott, 1884)], might fail to perceive an extra spatial dimension (the 3rd dimension) that we are capable of perceiving as they are computationally simpler than us.

Hence, *N*-Frame (Edwards, 2023, 2024) suggests that the conscious interface is potentially adjustable by the energy available to the organism (the conscious observer), and ultimately constrained by gravity (see Table 1). Some higher level (on the Kardashev scale such as 3 and 4) civilizations may be able to manipulate the computational constraint of their conscious interfaces via universal scale engineering such as blackhole Hawking radiation. Consistent with Lagrangian mechanisms in the free energy principle of Markov blankets, these information constraints on the organism’s conscious interface can be understood as reducing the thermodynamic cost of information. Greater informational boundary (greater thermodynamic cost) would lead to greater minimizing of prediction error, whereby the constraint in informational boundary of the observer optimizes the system’s internal representation given the finite energy available to them (or that they can manipulate via engineering). This reduction in information translates to lower energy burdens on the observer, making it an efficient and pragmatic strategy for encoding and processing information given existing constraints on the observer. The parameter set for the interface can be described by evolutionary fitness (see Figure 1) whereby we are not observing the real uncompressed world which is too computationally heavy for any observer [or at least lower level observers on the Kardashev scale such as humans—we are about 0.7 on this scale (Kaku, 2012)] to understand and therefore it exceeds their (and our, humans as observers *C*_*intO*_) epistemic limit. We therefore perceptually see a highly compressed reality within 3D + 1 (4D) encoded interface.

## 5 QBsim, *N*-Frame, and functional contextualism

This connection involves reinterpreting the Born rule from a functional contextualist perspective, which emphasizes the role of context and the functions of behavior (or in this case, measurement outcomes) in shaping our understanding of quantum probabilities. Crucially, *N*-Frame (Edwards, 2023, 2024) centers the observer (a conscious internal observer of the universe as a system *C*_*intO*_ and as part of that system self-referentially) at the heart of all of our interpretations and epistemological understanding of the universe, which shapes our ontological worldview. This emphasis on the observer is central to *N*-Frame (Edwards, 2023, 2024) because functional contextualism suggests that behavior (and cognition) cannot be understood independently of the context in which it occurs, and therefore the observer plays a central role in interpreting, analyzing, and influencing that behavior within its context.

Functional contextualism (Biglan and Hayes, 1996, 2015; Gifford and Hayes, 1999; Hayes and Gregg, 2001) is a philosophical worldview that focuses on predicting and influencing events by understanding the function of behavior within a given context. Functional contextualism is a philosophy of science rooted in philosophical pragmatism and contextualism. Stephen C. Pepper in his book “*World Hypothesis: A Study in Evidence*” (Pepper, 1942), describes the contextualism component of functional contextualism, whereby contextualism is Pepper’s own term for philosophical pragmatism. Pragmatism is a philosophical tradition from philosophers such as Peirce (1905), James (1907), and Dewey (1908) that assume words (language) and thought (thinking, decision-making) are tools for prediction, and problem-solving and action (behavior). Functional contextualism rejects the idea that the function of thoughts (the mental world) and language are a direct homomorphic representation (a mirror reality) to some veridically “real” world. The root metaphor of Pepper’s contextualism (Pepper, 1942) is “act in context,” which means that any act (or behavior, whether verbal or physical) is inseparable from its current and historical context. In line with the root metaphor, the truth criterion of Pepper’s contextualism is “successful working,” whereby the truth of an idea lies in its function or utility (utility as a goal) and not how well it homomorphically mirrors some underlying reality.

From this philosophical functional contextual standpoint, *N*-Frame (Edwards, 2023, 2024) assumes our perceptual conscious interface allows us to perceive some external world but not in a way that is homomorphic to the underlying reality, it simply represents a user interface that promotes the evolutionary survival and reproduction of the conscious entity. This view is supported by evolutionary game theory simulations (Hoffman and Prakash, 2014; Hoffman et al., 2015). Crucially, in relation to Penrose’s arguments about Gödel’ (1931) incompleteness, *N*-Frame (Edwards, 2023, 2024) emphasizes that the meaning and truth of statements are dependent on their context and functional practical utility.

As *N*-Frame (Edwards, 2023, 2024) assumes Bayesian subjective dynamics of quantum mechanics based on QBism (Fuchs, 2014, 2023). This connection involves reinterpreting the Born rule from a functional contextualist perspective, which emphasizes the role of context and the functions of behavior (or in this case, measurement outcomes) in shaping our understanding of quantum probabilities. To connect the standard Born rule and the QBist interpretation using functional contextualism, we can reinterpret the Born rule as a functional relation that depends on the context provided by the measurement setting and the observer’s interactions with the system. From a functional contextualist perspective, the probability assigned by the Born rule is not an inherent property of the quantum system alone but is a function of; (1) The measurement context, i.e., the specific experimental setup, including the observable being measured and the possible outcomes; (2) The observer’s *C*_*intO*_ agent’s actions (behaviors), i.e., the choices (decisions and cognitions) made by the observer *C*_*intO*_ in designing and performing the measurement; and (3) the functional relations i.e., the practical consequences of the measurement outcomes for the observer’s *C*_*intO*_ future actions and expectations.

This functional contextual interpretation of QM can be depicted via a mathematical reformulation, whereby consider an observer agent *C*_*intO*_ who assigns a quantum state |ψ⟩ based on their past experiences and current beliefs. The observer agent *C*_*intO*_ plans to measure observable $\hat{A}$ with eigenstate |*a*_*i*_⟩. The agent uses the Born rule to assign probabilities *P*(*a*_*i*_) to each possible outcome *a_i_*: *P*(*a*_*i*_) = *f*(|⟨*a*_*i*_|ψ⟩|^2^, *C*), where *f* is a function that incorporates the context *C* in which the measurement occurs. In QM, *f* is the identity function, but in functional contextualism, *f* may adjust probabilities based on contextual factors. From this reinterpretation, the standard Born rule and QBist probability assignments can be seen to be connected through functional contextualism. By acknowledging that quantum states, observables, and probabilities are context-dependent, we reconcile the objective mathematical formalism with subjective interpretations. This can be demonstrated formally via theorem and proof, called Functional Contextualism–QBism Connection Theorem.

To develop this theorem, we introduce context-dependent transformations and selection functions, and then show that the resulting formalism, when restricted appropriately, reduces to the standard Born rule and aligns with QBist interpretations, providing a bridge between standard QM and a subjective, context-dependent perspective. Here we can assume *H* to be a complex Hilbert space associated with a quantum system. A density operator ρ ∈ *D*(*H*) is a positive semi-definite operator on *H* with Tr(ρ) = 1. *D*(*H*) denotes the set of all density operators on *H*. An observable *A* is represented by a self-adjoint (Hermitian) operator $\hat{A}:H\to H$. A measurement of $\hat{A}$ is associated with a projection-valued measure {Π_i_}, where Π_*i*_ = |*a*_*i*_⟩⟨*a*_*i*_| are orthogonal projectors onto the eigenspaces of $\hat{A}$. In standard quantum mechanics, if the system is in state ρ and we measure the observable $\hat{A}$, the probability of obtaining outcome *a_i_* is *P*(*a*_*i*_) = Tr(ρΠ_i_). Note, this mathematical form of the Born rule *P*(*a*_*i*_) = Tr(ρΠ_i_) is the same in both standard quantum mechanics and QBism. What differs is the interpretation of the terms ρ and Π_i_. In standard QM, ρ is often viewed as an objective state of a system and Π_i_ as objective measurement projectors. In QBism, ρ and Π_i_ are taken as personal, subjective degrees of belief of an agent (an observer) and their chosen measurement settings. Thus, the formula is consistent with QM, but its meaning is shifted from an objective property of the system to a reflection of the observer agent’s $C_{i\,n\,t\,O}^{\prime }\,s$ subjective beliefs and choices.

This is accompanied by definitions for context *C*_*on*_, which is collection of parameters representing experimental conditions, environmental factors, and the internal (psychological, cognitive, historical) state of an observer *C*_*intO*_. Formally, $C_{o\,n}^{\prime }$ is an element of some parameter space*C*_*on*_. *C*_*intO*_ is the internal observer’s context represents any internal vantage point (subjective phenomenological states) or internal set of states, beliefs, and intentions attributed to an observer, and specifically whereby that observer is considered a subsystem of the universe or the internal observer to the universe, and within this broader context, so *C*_*intO*_⊆*C*_*on*_. Functional contextualism defines the probability assignments and state preparations are not intrinsic system properties, but functions of both the quantum state description and the context, including the observer’s internal states and decision-making processes. We then ascribe a set of assumptions or postulates; Postulate 1 (context state representation): There exists a family of completely positive trace-preserving (CPTP) maps ${\left\{E_{C_{o\,n}^{\prime }}:D\,\left(H\right)\to D\,\left(H\right)\right\}}_{C_{o\,n}^{\prime }\in C_{o\,n}}$ indexed by $C_{o\,n}^{\prime }\in C_{o\,n}$, such that the effective state under context $C_{o\,n}^{\prime }$ is given by $\rho \,C_{o\,n}^{\prime }:=E_{C_{o\,n}^{\prime }}\,\left(\rho \right)$. This postulate ensures that the context modifies the quantum state description in a completely positive and trace-preserving manner, reflecting how environmental conditions, prior knowledge, and observer intentions affect the effective state used for prediction. Postulate 2 (observer’s internal context and measurement choice): There exists a function *f*_*obs*_:*C*_*intO*_ → *O*, whereby *C*_*intO*_ ⊆ *C*_*on*_ is the space of internal observer contexts, and *O* is a space of operators representing initial measurement settings chosen by the observer. For an internal observer context $C_{i\,n\,t\,O}^{\prime }\in C_{i\,n\,t\,O}$ (i.e., a particular individual internal observer context, an element, is taken from a set of all possible internal observer contexts of all observers), such that $Q_{o\,b\,s}:=f_{o\,b\,s}\,\left(C_{i\,n\,t\,O}^{\prime }\right)$. This ensures that the observer’s internal state (context) selects the initial measurement operator. Postulate 3 (context-dependent measurement operator): There exists a function *f*_*means*_:*O* → *C*_*on*_ → *A*, whereby *A* is the set of observables, such that for given*Q*_*obs*_ and $C_{o\,n}^{\prime }\in C_{o\,n}$, then ${\hat{A}}_{C_{o\,n}^{\prime }}:=f_{m\,e\,a\,n\,s}\,\left(Q_{o\,b\,s},C_{o\,n}^{\prime }\right)$. ${\hat{A}}_{C_{o\,n}^{\prime }}$ is self-adjoint and depends continuously on $C_{o\,n}^{\prime }$. Postulate 4 (contextual probability assignments): There is a probability function *F*:*D*(*H*)×*A*×*C*_*on*_→[0,1], such that for any ρ ∈ *D*(*H*), ${\hat{A}}_{C_{o\,n}^{\prime }}\in A$, and $C_{o\,n}^{\prime }\in C_{o\,n}$: (1) Born consistency: If ${\hat{A}}_{C_{o\,n}^{\prime }}=\sum_{i}\{a_{i}\Pi_{\text{i}}\left(C_{o\,n}^{\prime }\right)$ then $F\left(\rho C_{o\,n}^{\prime },{\hat{A}}_{C_{o\,n}^{\prime }},C_{o\,n}^{\prime }\right)=\sum_{i}\text{Tr}\left[\rho C_{o\,n}^{\prime }\Pi_{\text{i}}\left(C_{o\,n}^{\prime }\right)\right]\}$, and these probabilities are normalized and non-negative. (2) Context reduction: If $E_{C_{o\,n}^{\prime }}=\text{Id}$ and ${\hat{A}}_{C_{o\,n}^{\prime }}=\hat{A}$ independent of $C_{o\,n}^{\prime }$, then $F\,\left(\rho ,\hat{A},C_{o\,n}^{\prime }\right)=\sum_{i}\text{Tr}\,\left(\rho \,\Pi_{\text{i}}\right)$, reducing to the standard Born rule. Postulate 5 (state update on measurement): Upon obtaining outcome *a_i_* under context $C_{o\,n}^{\prime }\in C_{o\,n}$, then $\rho \,C_{o\,n}^{\prime }=\frac{\Pi_{\text{i }}\,\left(C_{o\,n}^{\prime }\right)\,\rho \,C_{o\,n}^{\prime }\,\Pi_{\text{i }}\,\left(C_{o\,n}^{\prime }\right)}{\text{Tr[}\rho C_{o\,n}^{\prime }\Pi_{\text{i }}\left(C_{o\,n}^{\prime }\right)]}$, where $C_{o\,n}^{\prime \prime }$ is the new context after the measurement.

Theorem (functional contextualism and observer-centric QBism connection): Under the postulates of functional contextualism, which incorporate both the observer’s internal context and the external conditions, the Born rule emerges as a context-dependent probability assignment. In the limit of trivial context-dependence, the standard Born rule is recovered. Consequently, this framework provides a bridge between observer-centric (QBist) interpretations of functional contextualism and standard quantum mechanics, reconciling subjective probability updates with the usual Born rule.

Direct proof, step 1 (contextual state): From postulate 1, for any $C_{o\,n}^{\prime }\in C_{o\,n}$, $\rho \,C_{o\,n}^{\prime }=E_{C_{o\,n}^{\prime }}\,\left(\rho \right)$. Since *E*_*C’_on*_ is CPTP, $\rho \,C_{o\,n}^{\prime }$ is a valid density operator. Step 2 (observer measurement choice): From postulate 2, for any observer context $C_{i\,n\,t\,O}^{\prime }\in C_{i\,n\,t\,O}$, $Q_{o\,b\,s}=f_{o\,b\,s}\,\left(C_{i\,n\,t\,O}^{\prime }\right)$. Step 3 (context-dependent measurement operator): From postulate 3, given *Q*_*obs*_ and $C_{o\,n}^{\prime }$, ${\hat{A}}_{C_{o\,n}^{\prime }}=f_{m\,e\,a\,n\,s}\,\left(Q_{o\,b\,s},C_{o\,n}^{\prime }\right)$. Step 4 (spectral decomposition): ${\hat{A}}_{C_{o\,n}^{\prime }}$ admits a spectral decomposition ${\hat{A}}_{C_{o\,n}^{\prime }}=\sum_{i}a_{i}\,\Pi_{\text{i}}\,\left(C_{o\,n}^{\prime }\right)$, and $\Pi_{\text{i}}\,\left(C_{o\,n}^{\prime }\right)=\left|a_{i}\,\left(C_{o\,n}^{\prime }\right)\right\rangle \,\left\langle a_{i}\,\left(C_{o\,n}^{\prime }\right)\right|$. Step 5 (contextual probability): By postulate 4, $P\left(a_{i}|C_{o\,n}^{\prime }\right)=F\left(\rho C_{o\,n}^{\prime },{\hat{A}}_{C_{o\,n}^{\prime }},C_{o\,n}^{\prime }\right)=\text{Tr}\left(\rho C_{o\,n}^{\prime }\Pi_{\text{i}}\left(C_{o\,n}^{\prime }\right)\right)$. This ensures normalization and positivity of probabilities. Step 6 (reduction to standard Born rule): If $E_{C_{o\,n}^{\prime }}\,\left(\rho \right)=\rho$ and ${\hat{A}}_{C_{o\,n}^{\prime }}=\hat{A}$ independent of $C_{o\,n}^{\prime }$, $P\left(a_{i}|C_{o\,n}^{\prime }\right)=\mathrm{Tr}\left(\rho \Pi_{\text{i}}\right)$, the standard Born rule. Step 7 (subjective interpretation): In QBism, ρ and the choice of ${\hat{A}}_{C_{o\,n}^{\prime }}$ reflect the observer’s personal beliefs and measurement choices, Thus, the probabilities $P\left(a_{i}|C_{o\,n}^{\prime }\right)$ are subjective and context-dependent. Step 8 (state update): From postulate 5, $\rho \,C_{o\,n}^{\prime \prime }=\frac{\Pi_{\text{i }}\,\left(C_{o\,n}^{\prime }\right)\,\rho \,C_{o\,n}^{\prime }\,\Pi_{\text{i }}\,\left(C_{o\,n}^{\prime }\right)}{\mathrm{Tr}\,\left(\rho \,C_{o\,n}^{\prime }\,\Pi_{\text{i }}\,\left(C_{o\,n}^{\prime }\right)\right)}$.

In conclusion of this proof, starting from the postulates, we have derived a context-dependent probability rule $P\left(a_{i}|C_{o\,n}^{\prime }\right)=\text{Tr}\left[\rho C_{o\,n}^{\prime }\Pi_{\text{i}}\left(C_{o\,n}^{\prime }\right)\right]$ (as identified in step 5) which naturally reduces to the standard Born rule in the limit of trivial context dependence. This formulation aligns with QBist interpretations, where the probabilities are treated as observer-dependent assignments rather than intrinsic properties of the system. This result formally connects standard quantum mechanics, functional contextualism, and QBism, showing that quantum probability can be understood as a context-sensitive function of both the observer’s internal cognitive state (context) $C_{i\,n\,t\,O}^{\prime }$ and the external measurement context $C_{o\,n}^{\prime }$. More specifically, by starting with a set of assumptions that explicitly incorporate observer-centric contextual factors, we have constructed a generalized probability rule for quantum measurement outcomes. When these additional contextual dependencies are absent or trivial, the framework naturally collapses back to the standard Born rule of quantum mechanics. At the same time, the interpretational shift introduced by this framework is consistent with QBism, where quantum states and probabilities are regarded as subjective epistemic assignments rather than objective properties of reality. This reinforces the view that quantum mechanics does not merely describe an external, fixed physical reality *P* but instead encodes the observer’s beliefs and interactions with the system. This provides some further support for some equivalencing between the observer’s *C*_*intO*_ conscious state context and the physical world of the tri-aspect monist equivalence principle Ψ → Φ ≡ *C*_*intO*_ ≡ *P*.

In principle, one could embed this contextual, QBist-based rule within an AdS/CFT-like framework by viewing each observer’s context ($C_{i\,n\,t\,O}^{\prime }$, $C_{o\,n}^{\prime }$) as part of the boundary data specifying how the bulk geometry (or fields) evolve. The CPTP map *E*_*C’_on*_ could be understood as a boundary-to-bulk encoding that modifies the effective state ρ. When the observer’s internal conscious state or external conditions vary, the boundary conditions in the CFT shift resulting in a bulk state that mirrors these subjective changes. Under trivial boundary conditions (no observer-specific context), the standard AdS/CFT duality reproduces the usual Born rule. However, when the observer’s context, including conscious intent, is introduced, this context appears in the boundary theory as a set of additional constraints or operators, ultimately shaping the emergent bulk physics.

This perspective offers a route for exploring how quantum probabilities, typically seen as subjective in QBism (Fuchs, 2014, 2023) and *N*-Frame (Edwards, 2023, 2024), might correlate with the geometry and dynamics described by AdS/CFT. In this interpretation, the observer’s epistemic state is not merely a passive Bayesian update but instead plays an active role in defining the structure of the observer’s accessible reality. The self-referential nature of this model implies that the boundary conditions are not static but dynamically evolve with the observer’s interactions and internal cognitive processes, analogous to how AdS/CFT describes holographic encoding of gravitational degrees of freedom at the boundary. From this standpoint, the observer-centric approach in *N*-Frame suggests that quantum probabilities are not just context-dependent assignments but manifest as boundary data governing bulk emergence. The CPTP evolution map *E*_*C’_on*_ then functions as a process for translating observer-dependent information into bulk geometric structures, reinforcing the idea that physical reality, as perceived by a conscious observer, is an emergent holographic projection constrained by information-theoretic principles. This aligns with QBism’s view that quantum states encode an observer’s expectations but extends it by placing those expectations within a formal holographic encoding process, where subjective Bayesian updates correspond to changes in boundary conditions that shape the bulk space-time representation. Thus, within this extended framework, the conscious observer’s epistemic updates, represented by shifts in context $C_{i\,n\,t\,O}^{\prime }$, are directly linked to the holographic screen defining the projection of reality. This supports the equivalence between quantum probability evolution, AdS/CFT boundary conditions, and the self-referential encoding of conscious experience, providing a potential geometric interpretation for the functional contextualism of *N*-Frame.

This, of course, is a highly level overview of the functional contextual, QBism approach and their direct application within *N*-Frame (Edwards, 2023, 2024). In order to bridge the theoretical framework of functional contextualism and QBism with psychological experiments that have a QM explanation such as Radin-type consciousness-influenced collapse models (Baer, 2015; Bierman, 2003; Bösch et al., 2006; Ibison and Jeffers, 1998; Radin, 2008; Radin and Delorme, 2021; Radin et al., 2015a; Radin et al., 2016; Radin et al., 2012; Radin et al., 2013; Radin et al., 2015b; Radin et al., 2019; Vieten et al., 2018) and quantum probability theory (QPT) (Pothos and Busemeyer, 2022), we can introduce a more specific formal process by which an observer’s conscious intent affects measurement outcomes. Here, we can consider extending the standard quantum formalism by including a Hilbert space *H*_*intO*_ dedicated to representing the observer’s internal conscious states and identify within it a particular “focus” state $|\psi_{i\,n\,t\,O}^{f\,o\,c\,u\,s}$, which encapsulates the observer’s directed intention or attention. By coupling this internal state to the quantum system of interest through a carefully chosen interaction Hamiltonian ${\hat{H}}_{i\,n\,t}=\lambda \,\left({\hat{A}}_{s\,y\,s}\,⊗{\hat{A}}_{i\,n\,t\,O}\right)$, where λ is a small coupling constant, we introduce a subtle yet non-negligible influence of consciousness on the system’s evolution. Here, ${\hat{A}}_{s\,y\,s}$ is an observable of the system, and ${\hat{A}}_{i\,n\,t\,O}$ is an operator acting on the observer’s conscious Hilbert space, whereby ${\hat{A}}_{i\,n\,t\,O}=\left|\psi_{i\,n\,t\,O}^{f\,o\,c\,u\,s}\right\rangle \,\left\langle \psi_{i\,n\,t\,O}^{f\,o\,c\,u\,s}\right|$ its acts as a projector reflecting the observer’s focused intent. ⊗ is a standard tensor product.

Through perturbation theory, the standard Born rule probabilities are slightly modified by a function $g\,\left(\left|\psi_{i\,n\,t\,O}^{f\,o\,c\,u\,s}\right\rangle \right)$ that depends on the overlap between the observer’s initial conscious state and their focused state. This is a contextual weighting function that modifies quantum measurement probabilities based on the observer’s focused conscious state. It adjusts the standard Born rule probability by incorporating the observer’s intent or attention. It acts as a scaling factor that depends on the overlap between the observer’s internal state and their “focused” state of consciousness. Mathematically, it’s a function *g*:*H*_*intO*_→[0,1], whereby *H*_*intO*_ is the Hilbert space representing the observer’s consciousness. In the absence of conscious influence (η = 0), the familiar standard quantum probabilities are recovered. However, for small but non-zero η, the model predicts measurable shifts in outcome probabilities, effectively integrating the observer’s internal context *C*_*intO*_ (conscious intent) into the probability assignments. To clarify, the observer’s internal context *C*_*intO*_ represents their subjective state, including their beliefs, expectations, and attentional focus, which influences how they interact with a quantum system. Conscious intent (or focused attention) is a specific part of this internal context, modeled as a state in the observer’s Hilbert space *H*_*intO*_. This is why we use $\left|\psi_{i\,n\,t\,O}^{f\,o\,c\,u\,s}\right\rangle$ to represent the focused state of consciousness, it captures the observer’s intentional direction toward influencing a particular measurement outcome.

This explicit dynamical *N*-Frame model (Edwards, 2023, 2024) thus extends the abstract notion of context from the functional contextualism–QBism theorem to include the observer’s conscious states, offering a concrete model to account for Radin’s (and others) findings (approximately a 5 sigma finding) whereby human conscious intent has been shown to perturb the quantum waveform (Baer, 2015; Bierman, 2003; Bösch et al., 2006; Ibison and Jeffers, 1998; Radin, 2008; Radin and Delorme, 2021; Radin et al., 2015a; Radin et al., 2016; Radin et al., 2012; Radin et al., 2013; Radin et al., 2015b; Radin et al., 2019; Vieten et al., 2018), and can be understood, modeled, and tested within the quantum probabilistic framework of *N*-Frame (Edwards, 2023, 2024). In these experiments, they typically use double-slit experiments or interferometers, for example, see Figure 3A for an interference pattern identified without an observer or detector in a double-slit experiment (no perturbation), see Figure 3B for an example of the collapse of the waveform with a detector (perturbation via a physical detector), and Figure 3C for an example of conscious intent (perturbation via conscious intent) perturbing the waveform (Raiden type experiments). *N*-Frame (Edwards, 2023, 2024) therefore extends von Newmann’s (von Neumann, 1932) conscious chain proposed that the measurement process involves a chain of interactions such as quantum system → interacts with → measurement device → interacts with → observer’s brain (neural states) → interacts with → observer’s consciousness (subjective experience) In standard quantum mechanics, this chain terminates in the observer’s consciousness, but von Newmann did not specify a formal process for consciousness influencing measurement outcomes. *N*-Frame (Edwards, 2023, 2024) provides this process, by suggesting the human participant entangles with the quantum system via focused attention and then their conscious focus ${\hat{A}}_{i\,n\,t\,O}=\left|\psi_{i\,n\,t\,O}^{f\,o\,c\,u\,s}\right\rangle \,\left\langle \psi_{i\,n\,t}^{f\,o\,c\,u\,s}\right|$ acts as an explicit part of the collapse process, which explicitly models the observer’s focused conscious state as a projector in Hilbert space. This suggests that focused conscious intent acts as an operator that modifies quantum probabilities, influencing wavefunction collapse through contextual weighting $g\,\left(\left|\psi_{i\,n\,t\,O}^{f\,o\,c\,u\,s}\right\rangle \right)$. Thus, *N*-Frame (Edwards, 2023, 2024) provides a functional contextualist extension of von Neumann’s measurement chain, where the observer’s conscious state explicitly modulates wavefunction collapse through an interaction Hamiltonian and a contextual projection.

More specifically, this perturbation approach can be given a more detailed mathematical formalism, that would require a definition of a conscious state perturbation. Here, we can let the conscious focus state *H*_*intO*_ be $\left|\psi_{i\,n\,t\,O}^{f\,o\,c\,u\,s}\right\rangle$, selected by intent to influence a specific measurement outcome. The probabilistic effect of $\left|\psi_{i\,n\,t\,O}^{f\,o\,c\,u\,s}\right\rangle$ on the wavefunction |ψ⟩ in *H* can be defined as $P\left({\text{a}}_{i}|C_{i\,n\,t\,O},C_{o\,n}\right)=|\rangle {\text{a}}_{i}\left(C_{o\,n}\right)|\psi \rangle {|}^{2}\times g\left(|\psi_{i\,n\,t\,O}^{f\,o\,c\,u\,s}\right\rangle )$, whereby $g\,\left(\left|\psi_{i\,n\,t\,O}^{f\,o\,c\,u\,s}\right\rangle \right)$ represents the effect of focused consciousness on the probability of outcome a_*i*_. Then the modified measurement operator (contextual measurement adjustment) is defined as ${\hat{A}}_{C_{o\,n}}={\hat{U}}_{C_{o\,n}}\,{\hat{Q}}_{o\,b\,s}\,{\hat{U}}_{C_{o\,n}}^{\text{†}}$, whereby ${\hat{U}}_{C_{o\,n}}^{\text{†}}$ incorporates the focused intent from *C*_*intO*_. *C*_*intO*_ denotes the observer’s internal context, encompassing the observer’s beliefs, goals, intentions, and other subjective factors that might influence how the observer perceives or interacts with the quantum system. _a*i*_ represents the eigenvalues (possible measurement outcomes) of some observable $\hat{A}$. ${\hat{A}}_{C_{o\,n}}$ represents a context-dependent observable, meaning that its definition (the definition of the observable, i.e., it is not fixed it is contextual) depends on the external context *C*_*on*_. ${\hat{A}}_{C_{o\,n}}$ is not a fundamental property of the quantum system alone but is shaped by the experimental environment, making it a function of both the system and its context. In an AdS/CFT-like mapping of *N*-Frame, the context-dependent observable ${\hat{A}}_{C_{o\,n}}$ on the boundary corresponds to changes in the bulk field configurations, effectively linking quantum measurement updates to spacetime geometry. This formulation aligns with the tri-aspect monist equivalence principle Ψ → Φ ≡ *C*_*intO*_ ≡ *P*, where the physical *P*, phenomenological *C*_*intO*_, and informational (platonic Ψ→Φ) aspects of reality are fundamentally interrelated self-referentially. This introduces a functional contextualist extension to quantum mechanics, whereby measurement operators are not fixed but adapt based on contextual factors. ${\hat{U}}_{C_{o\,n}}$ is a unitary transformation encoding the effects of the external context *C*_*on*_. ${\hat{Q}}_{o\,b\,s}$ is the observer’s chosen measurement operator. ${\hat{U}}_{C_{o\,n}}^{\text{†}}$ is the Hermitian conjugate (inverse) of ${\hat{U}}_{C_{o\,n}}$, ensuring that measurement operators adapt to external context while preserving quantum consistency. ${\hat{U}}_{C_{o\,n}}$ is a unitary operator representing a transformation that depends on the external context *C*_*on*_. The Hermitian conjugate ${\hat{U}}_{C_{o\,n}}^{\text{†}}$ is its inverse, meaning that applying both in sequence restores the original system: ${\hat{U}}_{C_{o\,n}}^{\text{†}}\,{\hat{U}}_{C_{o\,n}}={\hat{U}}_{C_{o\,n}}\,{\hat{U}}_{C_{o\,n}}^{\text{†}}=I$, whereby *I* is the identity operator. In quantum mechanics, unitary operators preserve norms and probabilities, ensuring that physical transformations remain valid. At the level of *N*-Frame’s AdS/CFT boundary model, ${\hat{U}}_{C_{o\,n}}^{\text{†}}$ could represent a boundary-to-bulk mapping, where the observer’s choice of measurement affects the underlying bulk physics.

The probability of obtaining outcome a_*i*_ is therefore influenced by both internal and external observer contexts *P*(a_*i*_|*C*_*intO*_,*C*_*on*_) = |⟨a_*i*_(*C*_*on*_)|ψ⟩|^2^. The observable outcome is thus the result of a complex interplay of conscious intent (internal) and environment (external), aligning with findings that consciousness can appear to influence quantum systems. Consciousness therefore does not directly perturb the wavefunction but modifies the probabilities of outcomes, effectively reshaping the measurement process. The boundary conditions in the CFT (of AdS/CFT) (observer’s context and measurement setup) determine how information is projected into the bulk. This suggests that changes in the observer’s internal state correspond to shifts in probability distributions, rather than physical alterations of the wavefunction. Through an entanglement-like interaction (or contextual connection via entanglement), the observer’s intent within *H*_*intO*_ could indeed create observable shifts in the quantum system that would explain results that have been empirically detected in many experiments (Baer, 2015; Bierman, 2003; Bösch et al., 2006; Ibison and Jeffers, 1998; Radin, 2008; Radin and Delorme, 2021; Radin et al., 2015a; Radin et al., 2016; Radin et al., 2012; Radin et al., 2013; Radin et al., 2015b; Radin et al., 2019; Vieten et al., 2018). This framework supports the possibility that conscious intent can influence quantum events, explaining Radin-type observations as consciousness-mediated adjustments in wavefunction collapse (actualization) probabilities through internal and external contextual interaction. This *N*-Frame model could therefore be directly applied to AI as a direct test for consciousness as well as humans (Edwards, 2024).

Here, we can define: (Hilbert spaces) (1.1) a quantum system Hilbert Space *H*_*sys*_, that represents the quantum system under observation. The states in this system are denoted as |ψ_*sys*_⟩. (1.2) The observer’s conscious Hilbert space *H*_*intO*_, which represents the observer’s internal conscious states. The states in this space are denoted as |ψ_*intO*_⟩. This differs from *C*_*intO*_ which is the internal context of the observer as a high-level functional conceptual entity (description). *C*_*intO*_ includes a set of parameters [e.g., beliefs (prior knowledge), perspective, goals, attentional focus, cognitive states, and intentions] that influence how an observer interprets or modifies their measurement outcomes. *H*_*intO*_ is instead a Hilbert space formulation, that represents the observer’s conscious state as a quantum state in a Hilbert space. This Hilbert space *H*_*intO*_ mathematically encodes the observer’s state, allowing quantum mechanical operations like superposition, unitary evolution, and interaction. The observer’s internal context *C*_*intO*_ selects or constrains which states in *H*_*intO*_ are activated during a measurement process. It is a conceptual framework for understanding how the observer’s internal state interacts with measurements. For example, if an observer is focused on detecting a specific quantum property (e.g., spin-up vs. spin-down), their internal context (e.g., intent, focus, or prior beliefs) *C*_*intO*_ not only shapes how they interpret measurement results but also contributes to the selection of measurement operators. In this framework, *C*_*intO*_ modifies the context-dependent observable ${\hat{A}}_{C_{o\,n}}$, influencing which aspects of the quantum state are probed. This affects the probability distribution of measurement outcomes and how the system updates post-measurement. From the *N*-Frame perspective and QBism-consistent model, the observer’s internal context *C*_*intO*_ does more than just affect probability assignments, it also shapes the observer’s interface (observer’s perceived reality), influencing how information is encoded and updated within the measurement process. In *N*-Frame, the conscious observer’s interface is not a direct representation of objective reality but rather a contextualized projection of information. QBism’s subjective probability interpretation suggests that measurement outcomes are observer-dependent, meaning each observer’s interface (i.e., the way they extract and process information from reality) is uniquely shaped by their internal context. This is analogous to AdS/CFT, where bulk dynamics are “projected” onto a lower-dimensional boundary, and the boundary conditions (observer’s context) determine how bulk information is structured. The probability function for contextual probability updates that modify the observer’s interface is *P*(a_*i*_|*C*_*intO*_,*C*_*on*_) = Tr[ρ*C*_*on*_Π_*i*_(*C*_*on*_)] which shows that measurement probabilities are conditioned on both internal and external context. This means that when an observer updates their beliefs (QBism-style probability update), it does not just change their epistemic state but actively reshapes how future measurements are structured. This aligns with *N*-Frame’s consciousness-interface hypothesis, whereby *C*_*intO*_ helps determine the way quantum information is represented and processed. Since the observer’s internal state feeds back into the measurement via operator selection (through *Q*_*obs*_), the interface is self-referential. This mirrors QBism’s view that quantum mechanics describes the observer’s interactions with reality rather than reality “in itself.” However, *N*-Frame extends this idea by embedding the observer within an AdS/CFT-like information-processing structure, whereby quantum states in the bulk correspond to observer-dependent encodings on the boundary. *C*_*intO*_ determines how this encoding is structured, therefore shaping the observer’s conscious interface. The observer’s internal context *C*_*intO*_ is therefore not a passive element but an active variable shaping [consistent with Wheeler’s “it from bit” (Wheeler, 1992)] the way quantum events are perceived, interpreted, and integrated into broader knowledge structures. In an AdS/CFT-like mapping, changes in observer context *C*_*intO*_ correspond to boundary shifts, meaning that expectation-driven adjustments in CFT boundary conditions could subtly reshape the effective bulk state of the system, reinforcing the observer’s perceived quantum reality. In this sense *N*-Frame is not a model of reality but a model of reality as experienced and structured by the observer *C*_*intO*_ incorporating their interactions and constraints. (1.3) The total Hilbert space *H*_*total*_ represents the tensor of the system and consciousness Hilbert spaces *H*_*total*_ = *H*_*sys*_⊗*H*_*intO*_. The states in this space are denoted |ψ⟩ = *H*_*sys*_⊗|ψ_*intO*_⟩. (Operators) (2.1) The system Hamiltonian ${\hat{H}}_{s\,y\,s}$ governs the evolution of the quantum state. (2.2) The consciousness Hamiltonian ${\hat{H}}_{i\,n\,t\,O}$ governs the evolution of the observer’s conscious state. (2.3) The interaction Hamiltonian ${\hat{H}}_{i\,n\,t}$ represents the interaction between the observer’s *C*_*intO*_ consciousness and the quantum system. We will define this term carefully to model the influence of consciousness on the system. (2.4) The total Hamiltonian ${\hat{H}}_{t\,o\,t\,a\,l}$ sum of the system, consciousness, and the interaction Hamiltonians ${\hat{H}}_{t\,o\,t\,a\,l}={\hat{H}}_{s\,y\,s}\,⊗{\hat{I}}_{i\,n\,t\,O}+{\hat{I}}_{s\,y\,s}\,⊗{\hat{H}}_{i\,n\,t\,O}+{\hat{H}}_{i\,n\,t}$, whereby ${\hat{I}}_{s\,y\,s}$ and ${\hat{I}}_{i\,n\,t\,O}$ are identity operators on *H*_*sys*_ and *H*_*intO*_, respectively. (States) (3.1) The quantum state |ψ_*sys*_⟩ is an element of *H*_*sys*_ and can be in a superposition of eigenstates |ψ_*sys*_⟩ = ∑*ic*_*i*_|*a*_*i*_⟩. (3.2) Observer’s conscious state |ψ_*intO*_⟩ is an element of *H*_*intO*_, which represents the observer’s focus or intention.

We next define the interaction between consciousness and the quantum system via the interaction Hamiltonian ${\hat{H}}_{i\,n\,t}$. We propose an interaction Hamiltonian that models the influence of the observer’s conscious state on the quantum system: ${\hat{H}}_{i\,n\,t}=\lambda \,\left({\hat{A}}_{s\,y\,s}\,⊗{\hat{A}}_{i\,n\,t\,O}\right)$ whereby λ is a coupling constant quantifying the strength of the interaction, ${\hat{A}}_{s\,y\,s}$ is an observable of the quantum system, and ${\hat{A}}_{i\,n\,t\,O}$ is an operator acting on *H*_*intO*_, representing the observers focus. The properties of ${\hat{A}}_{i\,n\,t\,O}$ as a projection operator projects onto the observer’s focused conscious state ${\hat{A}}_{i\,n\,t\,O}=\left|\psi_{i\,n\,t\,O}^{f\,o\,c\,u\,s}\right\rangle \,\left\langle \psi_{i\,n\,t\,O}^{f\,o\,c\,u\,s}\right|$. The time system evolution under the total Hamiltonian (system) is governed by the Schrödinger equation $i\,\hbar \,\frac{d}{d\,t}\,\left|\Psi \,\left(\text{t}\right)\right\rangle ={\hat{H}}_{t\,o\,t\,a\,l}\,\left|\Psi \,\left(t\right)\right\rangle$ assuming the initial state is |Ψ(0)⟩ = |ψ_*sys*_(0)⟩⊗|ψ_*intO*_(0)⟩. We can formally integrate the Schrödinger equation to find $\left|\Psi \,\left(t\right)\right\rangle =e^{-i\,{\hat{H}}_{t\,o\,t\,a\,l}\,t/\hbar }\,\left|\Psi \,\left(0\right)\right\rangle$.

The next step is to calculate the modified probabilities. In the absence of consciousness interaction, the probability of finding the system in eigenstate |*a*_*i*_⟩ (standard quantum mechanics probability) is *P*_*standard*_(*a*_*i*_) = |⟨*a*_*i*_|ψ_*sys*_(*t*)⟩|^2^. In the modified probability due to conscious interaction, the probability is modified to $P\,\left(a_{i},t\right)={\left|\left\langle a_{i}|\left\langle \psi_{i\,n\,t\,O}^{f\,o\,c\,u\,s}|e^{-i\,{\hat{H}}_{t\,o\,t\,a\,l}\,t/\hbar }|\psi_{s\,y\,s}\,\left(0\right)\right\rangle \,⊗|\psi_{i\,n\,t\,O}\,\left(0\right)\right\rangle \right|}^{2}$. This expression represents the overlap between the evolved total state and the state where the system is in |*a*_*i*_⟩ and the observer is in the focused state $\left|\psi_{i\,n\,t\,O}^{f\,o\,c\,u\,s}\right\rangle$.

For perturbation (called the *N*-Frame (Edwards, 2023, 2024) perturbation approach), assuming the interaction is weak (λ is small), we can use time-dependent perturbation theory to first order. The first-order correction to the total state is $\left|\Psi^{\left(1\right)}\,\left(t\right)\right\rangle =\left|\Psi^{\left(0\right)}\,\left(t\right)\right\rangle -\frac{i}{\hbar }\,\int_{0}^{t}{\hat{H}}_{i\,n\,t}\,\left(t^{\prime }\right)\,\left|\Psi^{\left(0\right)}\,\left(t^{\prime }\right)\right\rangle \,dt^{\prime }$, whereby |Ψ^(0)^ (*t*)⟩ is the unperturbed evolution $\left|\Psi^{\left(0\right)}\,\left(t\right)\right\rangle =e^{-i{\hat{H}}_{s\,y\,s}⊗{\hat{I}}_{i\,n\,t\,O}+{\hat{I}}_{s\,y\,s}⊗{\hat{H}}_{i\,n\,t\,O})t/\hbar }\,\left|\psi_{s\,y\,s}\,\left(0\right)\right\rangle \,⊗\left|\psi_{i\,n\,t\,O}\,\left(0\right)\right\rangle$. The probability amplitude is $A\,\left(a_{i},t\right)=\left\langle a_{i}|\left\langle \psi_{i\,n\,t\,O}^{f\,o\,c\,u\,s}\right||\Psi^{\left(1\right)}\,\left(t\right)\right\rangle$, and expanding this we find *A*(*a*_*i*_,*t*) = *A*^(0)^(*a*_*i*_,*t*) + *A*^(1)^(*a*_*i*_,*t*), whereby *A*^(0)^(*a*_*i*_,*t*) is the unperturbed amplitude, and *A*^(1)^(*a*_*i*_,*t*) is the first-order correction due to the interaction. The first-order correction is $A^{\left(1\right)}\,\left(a_{i},t\right)=-\frac{i}{\hbar }\,\int_{0}^{t}\left\langle a_{i}|e^{i\,{\hat{H}}_{s\,y\,s}\,t^{\prime }/\hbar }\,{\hat{A}}_{s\,y\,s}\,e^{-i\,{\hat{H}}_{s\,y\,s}\,t^{\prime }/\hbar }|\psi_{i\,n\,t\,O}\,\left(0\right)\right\rangle \cdot \left\langle \psi_{i\,n\,t\,O}^{f\,o\,c\,u\,s}\right|\,e^{-i\,{\hat{H}}_{s\,y\,s}\,t^{\prime }/\hbar }\,{\hat{A}}_{i\,n\,t\,O}\,e^{-i\,{\hat{H}}_{i\,n\,t\,O}\,t^{\prime }/\hbar }|\psi_{i\,n\,t\,O}\,\left(0\right)\,\lambda \,d\,t^{\prime }$. This expression can be simplified, whereby assuming ${\hat{H}}_{i\,n\,t\,O}=0$ (i.e., the observer’s conscious state remains constant during the measurement process), and that ${\hat{A}}_{i\,n\,t\,O}=\left|\psi_{i\,n\,t\,O}^{f\,o\,c\,u\,s}\right\rangle \,\left\langle \psi_{i\,n\,t\,O}^{f\,o\,c\,u\,s}\right|$, we have $\left\langle \left|\psi_{i\,n\,t\,O}^{f\,o\,c\,u\,s}\right|\,{\hat{A}}_{i\,n\,t\,O}|\psi_{i\,n\,t\,O}\,\left(0\right)\right\rangle =\left\langle \psi_{i\,n\,t\,O}^{f\,o\,c\,u\,s}|\psi_{i\,n\,t\,O}^{f\,o\,c\,u\,s}\right\rangle \,\left\langle \psi_{i\,n\,t\,O}^{f\,o\,c\,u\,s}|\psi_{i\,n\,t\,O}\,\left(0\right)\right\rangle =\left\langle \psi_{i\,n\,t\,O}^{f\,o\,c\,u\,s}|\psi_{i\,n\,t\,O}\,\left(0\right)\right\rangle$. Similarly, the time evolution of ${\hat{A}}_{s\,y\,s}$ in the interaction picture is ${\hat{A}}_{s\,y\,s}\,\left(t^{\prime }\right)=e^{-i\,{\hat{H}}_{s\,y\,s}\,t^{\prime }/\hbar }\,{\hat{A}}_{s\,y\,s}\,e^{-i\,{\hat{H}}_{s\,y\,s}\,t^{\prime }/\hbar }$ . The first-order amplitude then becomes $A^{\left(1\right)}\,\left(a_{i},t\right)=-\frac{i\,\lambda }{\hbar }\,\left\langle \psi_{i\,n\,t\,O}^{f\,o\,c\,u\,s}|\psi_{i\,n\,t\,O}\,\left(0\right)\right\rangle \,\int_{0}^{t}\left\langle a_{i}|{\hat{A}}_{s\,y\,s}\,\left(t^{\prime }\right)|\psi_{s\,y\,s}\,\left(0\right)\right\rangle \,dt^{\prime }$. The total probability up to first order is *P*(*a*_*i*_,*t*) = |*A*^(0)^(*a*_*i*_,*t*) + *A*^(1)^(*a*_*i*_,*t*)|^2^, this can be expanded and keeping terms up to first order *P*(*a*_*i*_,*t*)≈|*A*^(0)^(*a*_*i*_,*t*)|^2^ + 2Re[*A*^(0)^(*a*_*i*_,*t*)*A^(1)^(*a*_*i*_,*t*)], whereby *A*^(0)^(*a*_*i*_,*t*)|^2^ is the standard probability *P*_*standard*_(*a*_*i*_). The second term represents the modification due to conscious interaction whereby *P*_*standard*_(*a*_*i*_) = |⟨*a*_*i*_|ψ_*sys*_(*t*)⟩|^2^ represents the standard QM probability and the consciousness-induced modification is Δ*P*(*a*_*i*_,*t*) = 2*Re*[*A*^(0)^(*a*_*i*_,*t*)*A^(1)^(*a*_*i*_,*t*)]. This modification depends on the overlap $\left\langle \psi_{i\,n\,t\,O}^{f\,o\,c\,u\,s}|\psi_{i\,n\,t\,O}\,\left(0\right)\right\rangle$ which represents the alignment between the observer’s initial conscious state and their focused intent. The matrix elements $\langle a_{i}|{\hat{A}}_{s\,y\,s}\left(t^{\prime }\right)|\psi_{s\,y\,s}\left(0\right)\langle$ which represents how the observable ${\hat{A}}_{s\,y\,s}$ connects the initial system state to the outcome |*a*_*i*_⟩ over time.

The next step is to define and model the conscious influence functions which quantifies how the observer’s focus modifies the probability distribution. This function can be defined as $g\,\left(\left|\psi_{i\,n\,t\,O}^{f\,o\,c\,u\,s}\right\rangle \right)$, whereby *g* can be defined as $g\,\left(\left|\psi_{i\,n\,t\,O}^{f\,o\,c\,u\,s}\right\rangle \right)=1+\eta \,{\left|\left\langle \psi_{i\,n\,t\,O}^{f\,o\,c\,u\,s}|\psi_{i\,n\,t\,O}\,\left(0\right)\right\rangle \right|}^{2}$ . Here, η is a small parameter quantifying the strength of consciousness influence, whereby when η = 0 there is no conscious influence, and *g=1*. The term $\left\langle \psi_{i\,n\,t\,O}^{f\,o\,c\,u\,s}|\psi_{i\,n\,t\,O}\left(0\right)\right\rangle {|}^{2}$ represents the alignment between the observer’s initial conscious state and their focused intent. Since the conscious influence function scales the probability, we adjust the modified probability to $P\,\left(a_{i},t\right)=P_{s\,t\,a\,n\,d\,a\,r\,d}\,\left(a_{i}\right)\cdot g\,\left(\left|\psi_{i\,n\,t\,O}^{f\,o\,c\,u\,s}\right\rangle \right)$. Substituting the expression for *g* gives us $P\left(a_{i},t\right)=P_{s\,t\,a\,n\,d\,a\,r\,d}\left(a_{i}\right)\left[1+\eta |\left\langle \psi_{i\,n\,t\,O}^{f\,o\,c\,u\,s}|\psi_{i\,n\,t\,O}\left(0\right)\right\rangle {|}^{2}\right]$. This shows that the observer’s focus introduces a multiplicative modification to the standard quantum probability. The effect depends on the inner product $\left\langle \psi_{i\,n\,t\,O}^{f\,o\,c\,u\,s}|\psi_{i\,n\,t\,O}\left(0\right)\right\rangle {|}^{2}$, meaning that greater alignment between the observer’s initial and focused states leads to a stronger influence. The influence is small when η is small, meaning this remains a perturbative correction rather than a fundamental restructuring of quantum mechanics. This perturbation modeling of *N*-Frame (Edwards, 2023, 2024) may help to account for small perturbation effects identified empirically in conscious intent experiments such as the following (Baer, 2015; Bierman, 2003; Bösch et al., 2006; Ibison and Jeffers, 1998; Radin, 2008; Radin and Delorme, 2021; Radin et al., 2015a; Radin et al., 2016; Radin et al., 2012; Radin et al., 2013; Radin et al., 2015b; Radin et al., 2019; Vieten et al., 2018).

From this formulation, the *N*-Frame (Edwards, 2023, 2024) model makes precise experimental predictions. Specifically, it predicts observable deviations from standard quantum mechanics whereby the probabilities of certain quantum events are slightly increased or decreased based on the observer’s focused conscious intent. Firstly, there are observable effects whereby the model predicts that the probabilities of certain quantum events can be slightly increased or decreased based on the observer’s focused conscious intent, which should be empirically detectable. The effect is proportional to η, which is expected to be small, consistent with the subtle influences reported in experiments (Baer, 2015; Bierman, 2003; Bösch et al., 2006; Ibison and Jeffers, 1998; Radin, 2008; Radin and Delorme, 2021; Radin et al., 2015a; Radin et al., 2016; Radin et al., 2012; Radin et al., 2013; Radin et al., 2015b; Radin et al., 2019; Vieten et al., 2018). Precise testable predictions can be made, whereby if an observer focuses on a specific outcome *a_i_*, the probability of observing *a_i_* should be significantly higher (likely a 5 sigma consistent with the other work mentioned) than predicted by standard quantum mechanics. The effect should be stronger when the observer’s initial conscious state |ψ_*intO*_(0)⟩ is closely aligned with the focused state $\left|\psi_{i\,n\,t\,O}^{f\,o\,c\,u\,s}\right\rangle$. This aspect of the *N*-Frame model is formulated by extending the standard framework of quantum mechanics by introducing an interaction Hamiltonian ${\hat{H}}_{i\,n\,t}$ of the observer. The conscious influence is treated as a perturbation, ensuring that the model reduces to standard quantum mechanics when λ = 0. Determining the values of λ and η requires experimental data. By explicitly modeling the role of consciousness in the collapse process, the *N*-Frame framework offers a potential resolution to the measurement problem. Consciousness from this perspective is not merely a passive observer process but an active participant influencing the outcome probabilities, giving a precise mathematical description for what Wheeler originally proposed in his “it from bit” (Wheeler, 1992).

Further formulization could also be incorporated such as a density matrix formalism to allow the model to account for mixed states and decoherence, we can employ the density matrix formalism. The density Matrix of the total system is $\hat{\rho }\,\left(t\right)=\left|\Psi \,\left(t\right)\right\rangle \,\left\langle \Psi \,\left(t\right)\right|$, and the reduced density matrix of the quantum system tracing over the observer’s conscious states is ${\hat{\rho }}_{s\,y\,s}\,\left(t\right)={\text{Tr}}_{i\,n\,t\,O}\,\left[\hat{\rho }\,\left(t\right)\right]$. This allows us to compute observable quantities for the quantum system alone. Environmental decoherence effects can also be included, such as by introducing the environmental Hilbert space *H*_*env*_. The total system becomes *H*_*total*_ = *H*_*sys*_⊗*H*_*intO*_⊗*H*_*env*_, whereby decoherence can suppress interference terms, affecting the probabilities. Decoherence suppresses interference terms, affecting the probabilities, however, decoherence should not be conflated with wavefunction collapse. In this *N*-Frame (Edwards, 2023, 2024) framework, the act of conscious observation influences quantum probabilities through the interaction Hamiltonian, potentially collapsing the system into a definite eigenstate. Once collapsed, the system remains in this definite state, but other entangled quantum degrees of freedom that were not directly observed do not necessarily collapse. Instead, these unobserved states decohere as they become entangled with the environment. For example, if an observer consciously focuses on a quantum object (such as a ball in superposition), the observer-induced collapse ensures that the ball assumes a definite state. However, surrounding environmental degrees of freedom, such as nearby particles, electromagnetic fields, or interacting atoms, do not undergo collapse but instead lose coherence over time due to environmental entanglement. This means that while conscious focus influences the collapse of the primary observed system, the larger quantum system may retain non-classical correlations until decoherence fully suppresses them. In this view, environmental decoherence acts as a passive process, gradually transitioning quantum coherence into classical-like behavior by dispersing information into the surrounding environment. In contrast, the observer’s conscious intent actively modifies quantum probabilities, distinguishing this model from traditional decoherence-only interpretations of quantum measurement.

However, this model thus far is not complete, as it does not resolve the measurement problem and does not specify the collapse of the wave function, it only describes a perturbation of a wavefunction. The measurement problem asks: (1) How does a superposition of states become a single outcome? (2) What causes collapse and when does it occur? (3) What role does the observer play in this process? *N*-Frame answers this by framing the observer *C*_*intO*_ as an intrinsic part of the quantum mechanical process, where the universe itself is described as a system in which the observer participates. *N*-Frame, aligned with QBism replaces the collapse process with a subjective observer *C*_*intO*_ belief updating *p*(*i*)→*p*(*j*) where probability updates are guided by the observer’s intent and context. This QM process can therefore be described either from the perspective of the observer updating their beliefs or as a collapse of the waveform (these are mathematically equivalent).

In line with a collapse description (simply aligning with the mainstream view of QM for simplicity), we can include non-Hermitian Hamiltonians in this *N*-Frame model (called the *N*-Frame collapse approach), to model collapse specifically (rather than perturbation) is important for modeling the cognitive collapse of the waveform. For this approach, the definitions of *H*_*sys*_, *H*_*intO*_, and *H*_*total*_ are applicable to both *N*-Frame perturbation and collapse models (both models share the fundamental system definitions), whereby both models describe the total states as |ψ⟩ = |ψ_*sys*_⟩⊗|ψ_*intO*_⟩. The interaction Hamiltonian is also the same in both modes, whereby models define the interaction Hamiltonian, though its role in the collapse model is more focused on guiding the system toward collapse ${\hat{H}}_{i\,n\,t}$$=\lambda \,\left({\hat{A}}_{s\,y\,s}\,⊗{\hat{A}}_{i\,n\,t\,O}\right)+\mu \,\left({\hat{A}}_{s\,y\,s}\,⊗{\hat{C}}_{i\,n\,t\,O}\right)$. Conscious and internal states such as the representation of |ψ_*focus*_⟩ (focused intent) and |ψ_*context*_⟩ (internal context) are also shared between both models. Novel definitions for the collapse model include non-unitary (non-reversible) dynamics during collapse, as non-Hermitian dynamics of collapse which ensure the Hamiltonian governing the system include the observer and their interaction as ${\hat{H}}_{t\,o\,t\,a\,l}=\left({\hat{H}}_{s\,y\,s}-i\,\hat{\Gamma }\right)\,⊗{\hat{I}}_{i\,n\,t\,O}+{\hat{I}}_{s\,y\,s}\,⊗{\hat{H}}_{i\,n\,t\,O}+{\hat{H}}_{i\,n\,t}$. This incorporates the non-Hermitian decay operator $-i\,\hat{\Gamma }$ for collapse dynamics, while the perturbation model maintains a Hermitian total Hamiltonian. This breaks time-reversibility, ensuring that once a state collapses, it cannot return to superposition (i.e., the physical world *P* that we perceive is fully rendered and irreversible from the internal observers *C*_*intO*_ perspective of that system). It introduces probabilistic transitions, consistent with the measurement postulate and the Born rule. In the perturbation model, probabilities are only slightly adjusted, but in the collapse model, the quantum state itself is reduced to a single eigenstate.

This *N*-Frame (Edwards, 2023, 2024) process uniquely integrates the system’s non-Hermitian collapse, the observer’s conscious evolution, and the interaction dynamics. This non-unitary process is important for wavefunction collapse, because this models a non-reversible process, that is probabilistic (based on the Born rules) rather than deterministic (based on the Schr$\ddot{\text{o}}$dinger equation). This ensures that the process is explained by the measurement postulate and Born rule which is central to accounting for the measurement problem (the observer *C*_*intO*_ as a measurement asking a question about the universe as a system self-referentially). In the perturbation model the interaction Hamiltonian ${\hat{H}}_{i\,n\,t}=\lambda \,\left({\hat{A}}_{s\,y\,s}\,⊗{\hat{A}}_{i\,n\,t\,O}\right)+\mu \,\left({\hat{A}}_{s\,y\,s}\,⊗{\hat{C}}_{i\,n\,t\,O}\right)$ introduces a perturbation to the quantum system due to the observer’s consciousness. This perturbation modifies the probabilities of different outcomes but does not, by itself, lead to the non-unitary collapse of the wavefunction. The evolution remains unitary because the total Hamiltonian ${\hat{H}}_{t\,o\,t\,a\,l}$ is Hermitian. ${\hat{A}}_{s\,y\,s}$ is the observable of the quantum system, ${\hat{A}}_{i\,n\,t\,O}$ represents the focused intent, and ${\hat{C}}_{i\,n\,t\,O}$ represents the internal context of the specific individual observer. Again, specific to the collapse model, the non-Hermitian decay operator $-i\,\hat{\Gamma }$ acts on *H*_*sys*_ that introduces decay or collapse dynamics. This ensures non-unitary evolution in the quantum system, driving the wavefunction collapse. Note, *N*-Frame favors an actualizing or rendering of information process whereby the observer’s interface is updating more consistent with QBism rather than a physical collapse interpretation, but this description is given to accommodate those with a collapse model understanding of QM for simplicity.

Collapse refers to the process where a quantum system transitions from a superposition of states to a single eigenstate upon measurement. In standard quantum mechanics, collapse is not derived from the Schrödinger equation but is postulated through the measurement postulate. The Schrödinger equation describes unitary evolution, which is reversible and deterministic, whereas collapse is non-unitary, irreversible, and probabilistic. Since the interaction Hamiltonian ${\hat{H}}_{i\,n\,t}$ is Hermitian, the combined evolution remains unitary. Unitarity implies that the total probability is conserved, and the evolution does not inherently include collapse. Time-dependent perturbation theory provides corrections to the system’s evolution due to small interactions. It adjusts the amplitudes and phases of the quantum states but does not result in the reduction of the wavefunction to a single eigenstate. Incorporating non-Hermitian terms in the Hamiltonian allows for non-unitary evolution, which can model decay processes and effectively represent wavefunction collapse. Non-Hermitian operators can lead to a decrease in the norm of the state vector, reflecting the reduction of the system’s state space during collapse. To mathematically model collapse within the Schrödinger equation, the evolution must be non-unitary. This requires introducing mechanisms that break unitarity, such as non-Hermitian terms, measurement operators, or stochastic processes.

To explicitly model the collapse mechanism within this framework, we therefore introduce non-Hermitian terms to the system Hamiltonian. We modify the system Hamiltonian ${\hat{H}}_{s\,y\,s}$ by adding a non-Hermitian term $-i\,\hat{\Gamma }$ representing the collapse process ${\hat{H}}_{s\,y\,s}\to {\hat{H}}_{s\,y\,s}-i\,\hat{\Gamma }$, whereby $\hat{\Gamma }$ is a semi-definite, Hermitian operator acting on *H*_*sys*_. It is important to note that while $\hat{\Gamma }$ is Hermitian, multiplying it by −*i* transforms it into an anti-Hermitian. The anti-Hermitian term represents processes that are not time-reversible and involve a loss of information or energy. In the context of collapse, it models the irreversible transition to a definite state. In quantum mechanics, the imaginary unit *i* appears in the Schrödinger equation and is essential for describing the unitary time evolution of quantum states. The negative imaginary unit −*i* is used when introducing terms that lead to decay or damping, representing processes that reduce the amplitude of certain states over time.

The next step is to update the total Hamiltonian ${\hat{H}}_{t\,o\,t\,a\,l}$ now becomes ${\hat{H}}_{t\,o\,t\,a\,l}=\left({\hat{H}}_{s\,y\,s}-i\,\hat{\Gamma }\right)\,⊗{\hat{I}}_{i\,n\,t\,O}+{\hat{I}}_{s\,y\,s}\,⊗{\hat{H}}_{i\,n\,t\,O}+{\hat{H}}_{i\,n\,t}$. This inclusion of $-i\,\hat{\Gamma }$ now allows us to model non-unitary evolution leading to collapse. The Schrödinger Equation can be modified as time evolution with non-Hermitian Hamiltonian. Time evolution is governed by $i\,\hbar \,\frac{d}{d\,t}\,\left|\Psi \,\left(t\right)\right\rangle ={\hat{H}}_{t\,o\,t\,a\,l}\,\left|\Psi \,\left(t\right)\right\rangle$, with the initial state unchanged |Ψ(0)⟩ = |ψ_*sys*_(0)⟩⊗|ψ_*intO*_(0)⟩. Due to the non-Hermitian term $-i\,\hat{\Gamma }$ (expressed within ${\hat{H}}_{t\,o\,t\,a\,l}$) the evolution is non-unitary, introducing decay of certain components of the quantum state, effectively modeling collapse.

The modified probabilities with collapse can now be calculated such as with first-order correction with non-Hermitian terms. We proceed with perturbation theory, treating ${\hat{H}}_{i\,n\,t}$ as a perturbation and incorporating the non-Hermitian term in the unperturbed Hamiltonian ${\hat{H}}_{O}=\left({\hat{H}}_{s\,y\,s}-i\,\hat{\Gamma }\right)\,⊗{\hat{I}}_{i\,n\,t\,O}+{\hat{I}}_{s\,y\,s}\,⊗{\hat{H}}_{i\,n\,t\,O}$. The first-order correction to the total state is $\left|\Psi^{\left(1\right)}\,\left(t\right)\right\rangle =\left|\Psi^{\left(0\right)}\,\left(t\right)\right\rangle -\frac{i}{\hbar }\,\int_{0}^{t}{\hat{H}}_{i\,n\,t}\,\left(t^{\prime }\right)\,\left|\Psi^{\left(0\right)}\,\left(t^{\prime }\right)\right\rangle \,dt^{\prime }$, whereby |Ψ^(0)^(*t*)⟩ evolves under ${\hat{H}}_{O}$. The expressions can be simplified, by assuming ${\hat{H}}_{i\,n\,t\,O}=0$ and ${\hat{A}}_{i\,n\,t\,O}=\left|\psi_{i\,n\,t\,O}^{f\,o\,c\,u\,s}\right\rangle \,\left\langle \psi_{i\,n\,t\,O}^{f\,o\,c\,u\,s}\right|$. The modified probability amplitude becomes $A\,\left(a_{i},t\right)=\left\langle a_{i}\,\left|\psi_{i\,n\,t\,O}^{f\,o\,c\,u\,s}\right||\Psi^{\left(1\right)}\,\left(t\right)\right\rangle$, with the first-order correction ${\text{A}}^{\left(1\right)}\,\left(a_{i},t\right)=-\frac{i\,\lambda }{\hbar }\,\left\langle \psi_{i\,n\,t\,O}^{f\,o\,c\,u\,s}|\psi_{i\,n\,t\,O}\,\left(0\right)\right\rangle \,\int_{0}^{t}\left\langle a_{i}|{\hat{A}}_{s\,y\,s}\,\left(t^{\prime }\right)|\psi_{s\,y\,s}\,\left(0\right)\right\rangle \,dt^{\prime }$, where ${\hat{A}}_{s\,y\,s}\,\left(t^{\prime }\right)$ now evolves under the non-Hermitian Hamiltonian ${\hat{A}}_{s\,y\,s}\,\left(t^{\prime }\right)$ now evolves under the non-Hermitian Hamiltonian ${\hat{A}}_{s\,y\,s}\,\left(t^{\prime }\right)=e^{i\,\left({\hat{H}}_{s\,y\,s}-i\,\hat{\Gamma }\right)\,t^{\prime }/\hbar }\,{\hat{A}}_{s\,y\,s}\,e^{i\,\left({\hat{H}}_{s\,y\,s}-i\,\hat{\Gamma }\right)\,t^{\prime }/\hbar }$ . The total probability up to the first order is *P*(*a*_*i*_,*t*) = |A^(0)^(*a*_*i*_,*t*) + A(1)(*a*_*i*_,*t*)|^2^, where A^(0)^(*a*_*i*_,*t*) includes the effect of the non-Hermitian term leading to collapse.

The next step is to define the collapse operator that models collapse toward a specific eigenstate |*a*_k_0__⟩, we define $\hat{\Gamma }=\gamma \,\sum_{k\,k_{0}}\left|a_{k}\right\rangle \,\left\langle a_{k}\right|$, whereby γ > 0 is the decay rate. This ensures that all states except |*a*_k_0__⟩ decay over time. The non-Hermitian term causes the amplitudes of the unwanted states to decrease exponentially, effectively collapsing the system into |*a*_k_0__⟩. The final probability expression includes both the collapse process and the conscious influence, the probability becomes $P\left(a_{i},t\right)=P_{c\,o\,l\,l\,a\,p\,s\,e}\left(a_{i},t\right)\cdot g\left(|\left\langle \psi_{i\,n\,t\,O}^{f\,o\,c\,u\,s}\right\rangle \right)$, whereby *P*_*collapse*_(*a*_*i*_,*t*) = |A^(0)^(*a*_*i*_,*t*)|^2^ includes the effects of the non-Hermitian collapse, and $g\left(|\left\langle \psi_{i\,n\,t\,O}^{f\,o\,c\,u\,s}\right\rangle \right)=1+\eta |\left\langle \psi_{i\,n\,t\,O}^{f\,o\,c\,u\,s}|\psi_{i\,n\,t\,O}\left(0\right)\right\rangle {|}^{2}$, accounts for the conscious influence. By introducing the non-Hermitian term $-i\,\hat{\Gamma }$ into the system Hamiltonian, we have extended the model to explicitly account for wavefunction collapse. The model now incorporates both the collapse mechanism and the perturbative influence of consciousness within the same *N*-Frame framework, providing a comprehensive approach to addressing the measurement problem in quantum mechanics. In relation to the Quantum Zeno Effect (QZE), this implies that frequent measurements inhibit the evolution of a quantum system. The non-Hermitian term models continuous measurement by consciousness.

In our *N*-Frame (Edwards, 2023, 2024) model, the non-Hermitian term $-i\,\hat{\Gamma }$ causes the decay of states not aligned with the observer’s focused conscious state |*ϕ*_*k*_⟩. This decay mechanism effectively “freezes” the system into states matching the observer’s focus. This phenomenon is analogous to the QZE, where frequent measurements inhibit the evolution of a quantum system, preventing it from transitioning away from its initial state. By continuously “observing” the system through focused consciousness, the observer acts similarly to performing frequent measurements. The non-Hermitian term represents this continuous observation, leading to the suppression of transitions to non-aligned states. Mathematically, the decay rate introduced by $\hat{\Gamma }$ can be associated with the measurement frequency in the QZE. A higher decay rate results in stronger suppression of unwanted states, reinforcing the alignment with the observer’s focus. This connection to the QZE provides additional theoretical support for our model. It suggests that consciousness can play an active role in the collapse process by inducing a QZE-like effect, effectively controlling the outcome probabilities through focused intent. This would explain why we see a physical world only and not a quantum one of superposition in every life.

It is important to note that *N*-Frame (Edwards, 2023, 2024) is consistent with the QBist approach, so though non-Hermitian account typically describes a physical interpretation of QM, from a QBist point of view, the “collapse” process described by non-Hermitian Hamiltonians and decay terms is not a physical mechanism acting on an objectively real wavefunction. Instead, it can be interpreted as a formal representation of how an agent updates their subjective probability assignments given certain contextual factors, internal focus states, and a chosen measurement scenario. In QBism, the quantum state ρ is a personal informational tool used by the agent to assign probabilities to possible outcomes, and “collapse” is simply the agent’s Bayesian update of these probabilities after receiving new data. Mathematically, we can frame this in terms of quantum instruments and Bayesian updating rather than invoking non-unitary dynamics on an objectively real state. Consider the agent’s initial personal state (density operator) ρ, which encodes their beliefs about the system. A measurement scenario, including any internal conscious influences, can be represented by a set of completely positive (CP) maps {*ℰ_i_*}, known as a quantum instrument. Each *ℰ_i_* corresponds to a particular outcome *a_i_* and acts on ρ to produce a (generally unnormalized) updated state *ℰ_i_*(ρ). Formally, a quantum instrument can be represented by a set of Kraus operators {*R*_*i*,*k*_} such that ${ℰ}_{i}\,\left(\rho \right)=\sum_{k}R_{i,k}\,\rho \,R_{i,k}^{†}$. In the simplest projective measurement scenario, these Kraus operators reduce to projectors (up to a unitary transformation). When the agent obtains outcome *a_i_*, they update their state via Bayes’ rule: $\rho \to \rho_{i}^{\prime }=\frac{{ℰ}_{i}\,\left(\rho \right)}{\text{Tr}\,\left[{ℰ}_{i}\,\left(\rho \right)\right]}$. This is the QBist and *N*-Frame version of “collapse”: a normative rule for rational belief updating rather than a physical, dynamical collapse (though *N*-Frame can accommodate this physical interpretation in line with its tri-aspect equivalence principle ψ → Φ ≡ *C*_*intO*_ ≡ *P*, as it is a tri-aspect model).

Now, how do we relate this *N*-Frame (Edwards, 2023, 2024) QBist interpretation to the non-Hermitian terms in the *N*-Frame “collapse” model outlined above? The non-Hermitian operators $-i\,\hat{\Gamma }$ and the associated decay factors γ_*i*_ introduced in the Hamiltonian from a *N*-Frame and QBist perspective they are not physically real forces that cause objective collapse. Instead, we may treat them as part of the agent’s chosen quantum instrument, a complex parameterization that captures how the agent’s internal context and focused intent (encoded by ${\hat{A}}_{i\,n\,t\,O}$ and ${\hat{C}}_{i\,n\,t\,O}$) bias their probability assignments and thus the relative weights of the instrument’s Kraus operators. In other words, the non-Hermitian terms can be absorbed into a set of measurement operators *M_i_* that reflect the agent’s subjective weighting of certain outcomes over others. For instance, consider a set of operators {*M*_*i*_} that incorporate both the conscious intent of the observer and the effective “decay” preferences induced by $-i\,\hat{\Gamma }$ . The updated rule for outcome probabilities becomes: $P\,\left(a_{i}\right)=\mathrm{Tr}\,\left(\rho \,M_{i}^{†}\,M_{i}\right)$, with the posterior state: $\rho \to \rho_{i}^{\prime }=\frac{M_{i}\,\rho \,M_{i}^{†}}{\mathrm{Tr}\,\left(\rho \,M_{i}^{†}\,M_{i}\right)}$. Here the *M_i_* operators, constructed to reflect the previously introduced $-i\,\hat{\Gamma }$ and interaction terms are interpreted as part of a complex, subjective instrument chosen by the agent, not as a literal, non-unitary evolution of an underlying physical reality. Thus, the same mathematical form used in the collapse model can be understood in QBism as a specification of the agent’s quantum instrument, influenced by their internal states and contexts. Instead of a physical collapse induced by a non-Hermitian Hamiltonian, we have a Bayesian update step guided by these operators, which incorporate all the “collapse-like” behavior as an agent’s subjective reallocation of belief. This interpretation remains consistent with the *N*-Frame (Edwards, 2023, 2024) functional contextualism–QBism theorem’s emphasis on observer-dependent, context-sensitive probability assignments: all structural complexities be they non-Hermitian terms or perturbative expansions are reinterpreted as detailed ways of modeling how the agent shapes and updates their personal probability assignments, rather than processes enforcing an objective physical collapse. Note, *N*-Frame accounts for both of these interpretations as equivalent, ψ → Φ ≡ *C*_*intO*_ ≡ *P*, as it is a tri-aspect equivalence model.

## 6 Explaining why intuition can find truth without formal mathematical proof: the *N*-Frame non-computable (hypercomputation) Gödelian truth conjecture

Through this novel *N*-Frame (Edwards, 2023, 2024) perspective we can conceptualize and reframe the Gödel incompleteness argument, to explain how humans can through intuition know a proof is true without formal mathematical and logical proof. Gödel’ (1931) First Incompleteness Theorem establishes that any consistent, effectively generated formal system *S* rich enough to represent basic arithmetic contains statements that are true but not provable within *S*. More concretely, there exists a Gödel sentence *G* expressible in *S* such that: *S* does not prove (*S*⊬*G*), *S* does not prove ¬*G*(*S*⊬¬*G*). Under the standard interpretation of arithmetic, *G* is true but unprovable in *S*. This stark limitation, i.e., that there are truths beyond the system’s reach, may seem insurmountable within the realm of pure mathematics. However, when we broaden our perspective to include physical interpretive frameworks, such as QBism and the *N*-Frame (Edwards, 2023, 2024) perturbation/collapse approach, we encounter a more flexible conception of what it means to “recognize” or “find” truth. In these frameworks, an observer’s conscious intuition, informed by their internal states, focus, and contextual understanding, can guide them to effectively extend or alter their foundational assumptions. This process is analogous to introducing a new axiom *A* into *S*, forming *S+A*, thereby allowing previously unprovable statements like *G* to become derivable i.e., now (*S* + *A*)⊢*G*. Although we cannot produce a formal arithmetic proof of *G* within the original *S*, we can “reach” *G* through a meta-level (effectively hypercomputation) decision to enrich our conceptual framework.

In order to make this argument concretely, let *S* be a formal system meeting Gödel’s criteria: it is consistent, effectively generated, and can represent basic arithmetic. Gödel’s theorem ensures the existence of a Gödel sentence *G* such that no series of axiom applications and inference rules within *S* proves *G*. Yet, *G* is true under the standard interpretation of the natural numbers ℕ. Hence, we face a logical impasse in that formal derivation alone cannot deliver all truths. Now, consider a physical scenario involving a conscious observer who employs a QBist/perturbation-collapse approach. In standard quantum theory, measurements and states are often taken as objective features of reality. QBism and *N*-Frame models, however, emphasize the role of the observer’s internal perspective: quantum states are interpreted as personal degrees of belief, and measurement outcomes are shaped by the observer’s internal states and chosen instrument operators (Kraus operators).

Mathematically, let us represent the observer’s measurement scheme by a set of operators {*M*_*i*_}, each acting on a Hilbert space. These operators determine the probabilities of measurement outcomes. Initially, the set {*M*_*i*_} corresponds to the agent’s starting point—analogous to the initial system *S* in the logical setting. The observer finds that certain phenomena (analogs to the statement *G*) are not satisfactorily explained or “derived” by this starting setup. In mathematics, when faced with an independent statement *G*, a common strategy is to add a new axiom *A* that resolves the independence and makes *G* provable in the extended system *S+A*. Similarly, in the observer-centric quantum framework, the agent can introduce a new operator $\hat{\Delta }$ or modify the measurement scheme to $\left\{M_{i}^{\prime }\right\}$ in a way that captures the elusive pattern corresponding to *G*. Concretely, the observer might define: ${\hat{M}}_{i}^{\prime }=g({\hat{A}}_{i\,n\,t\,O},{\hat{C}}_{i\,n\,t\,O}$$,\hat{\Gamma },\hat{\Delta })\hat{N}i$, where ${\hat{A}}_{i\,n\,t\,O}$ and ${\hat{C}}_{i\,n\,t\,O}$ represent the observer’s internal focus and context, $\hat{\Gamma }$ a non-Hermitian operator modeling effective collapse/decay dynamics, and $\hat{\Delta }$ is the newly introduced element guided by intuition. Just as the mathematician chooses *A* to make *G* provable, the observer chooses $\hat{\Delta }$ to “justify” outcome patterns analogous to the truth of *G*.

The crucial step, selecting *A* in the mathematical case or $\hat{\Delta }$ in the quantum/observer scenario, is not enforced by the original axioms or operators. Instead, it emerges from the observer’s conscious intuition, meta-theoretical reasoning, and conceptual coherence. This intuition might stem from aesthetic criteria (simplicity, elegance), empirical hints, context-dependent judgment, or theoretical analogies. It allows the observer to transcend the initial boundaries set by *S* or $\left\{{\hat{M}}_{i}\right\}$. In other words, while *S* could not prove *G*, the enriched setting *S+A* now does, and while the original $\left\{{\hat{M}}_{i}\right\}$ did not yield a satisfactory account for the elusive phenomenon, the modified $\left\{{\hat{M}}_{i}^{\prime }\right\}$, guided by $\hat{\Delta }$, brings that phenomenon into conceptual reach. Both processes illustrate how stepping beyond the original constraints, through a consciously chosen extension, lets us “find truth” that was previously inaccessible. The process of enriching a formal system with new axioms is well-known in mathematics, for example, mathematicians might add the Axiom of Choice to ZF set theory to obtain ZFC. The argument here extends that idea to quantum measurement and cognition. It shows that, even though the original system *S* cannot prove *G*, an enriched system *S+A* can, illustrating that truth can be “found” by stepping outside the original constraints of the system that defines the question. This provides a coherent bridge between formal logic and human intuition.

This argument does not undermine Gödel’s theorem; it still holds that from within the original system *S*, *G* is not provable. However, it demonstrates that human cognition and conscious intuition provide a meta-level resource to introduce new principles. Such principles can settle previously unprovable truths, at least from the enriched standpoint. In the quantum analogy, no “proof” of *G* in the arithmetic sense is ever found, but an analogous resolution occurs: what was once not derivable by the old measurement framework is now derivable by the refined framework that includes $\hat{\Delta }$ . This perspective suggests that while formal systems are inherently limited, the observer-as-part-of-the-universe approach, combined with interpretive frameworks like QBism within the broacher picture of *N*-Frame, allows us to navigate beyond formal strictures. Truth, in this sense, is not a static given but rather something that can be approached through iterative conceptual and axiomatic refinement. The observer’s intuition plays a central role, enabling them to add, modify, or reinterpret axioms and operators to achieve a more comprehensive understanding. Ultimately, this argument illustrates that human intuition, shaped by internal states and guided by a self-referential stance, can compensate for formal incompleteness by expanding the conceptual space in which truths reside. This shows how cognitive and interpretative capabilities provide a path to acknowledging and incorporating truths that elude the original system’s formal derivations.

From this, a formal *N*-Frame (Edwards, 2023, 2024) non-computable Gödelian truth conjecture can be formalized. A conditional derivation presented within a formal proof conceptual demonstration format to help readers see precisely how each assumption contributes to the conjectured conclusion. Postulate 1 (formal system representation): Let *F* be a consistent, recursively enumerable formal system capable of expressing basic arithmetic. There exists a configuration space *C*_*onfig*_ for an observer’s internal context *C*_*intO*_ ∈ *C*_*onfig*_, and a computable embedding map ι:{*AxiomsofF*}→{*Inferencerulesof*F}→*C*_*onfig*_. This embedding encodes the structure of *F* into the observer’s internal state, *C*_*intO*_(*F*) = ι(*F*). Postulate 2 (Gödelian statement): Within *F*, let *S* be a Gödelian statement that is true under the standard model of arithmetic but not provable in *F*. By Gödel’s incompleteness theorems, attempting to prove or disprove *S* in *F* leads to contradictions or inconsistency. Thus, *S* is undecidable (unprovable) within *F*. Postulate 3 (quantum setting and context): The observer interacts with a quantum system described by a separable Hilbert space *H*. External conditions *C*_*on*_ and internal conditions *C*_*intO*_ define a context in which quantum measurements are made. Measurement outcomes are associated with POVMs{Π_*i*_(*C*_*on*_,*C*_*intO*_)}. Postulate 4 (context-dependent state assignment): There exists a function *f*:*D*(*H*)×*C*_*on*_×*C*_*intO*_ → *D*(*H*) that produces a modified density operator ρ_*C*_*on*_,*C*_*intO*__ = *f*(ρ_*C*_*on*_,*C*_*intO*__). The probability of outcome *a_i_* is *P*(*a*_*i*_|*C*_*on*_,*C*_*intO*_) = Tr[ρ_*C*_*on*_,*C*_*intO*__Π_*i*_(*C*_*on*_,*C*_*intO*_)]. Postulate 5 (non-computable construction of *f*): The function *f* is defined as the limit of a sequence of computable functions {*f*_*n*_} each depending on whether a universal Turing machine *U*, when simulating a Turing machine *M* on input *w*, halts within *n* steps. Formally, let *M*,*w* be encoded into *C*_*intO*_. Choose two distinct density operators ρ_*H*_ρ_*NH*_. Define: $f_{n}\left(\text{ρ, }C_{on,}\;C_{into,}\right)=\{\begin{array}{c} {\text{ρ}}_{H}\text{ if }U\left(M,w\right)\text{halts within }n\text{ steps,}\\ {\text{ρ}}_{NH}\text{ otherwise} \end{array}$. Then set *f*(ρ,*C*_*on*_,*C*_*intO*_): = lim*n*→∞⁡*f*_*n*_(ρ,*C*_*on*_,*C*_*intO*_). If *M* halts on *w*, the limit is ρ_*H*_, otherwise it is ρ_*NH*_. Deciding this solves the Halting problem, ensuring *f* is non-computable. Postulate 6 (emergent gravity and complexity): In a holographic duality scenario (e.g., AdS/CFT), bulk geometry is related to complexity and entanglement patterns in boundary states |Ψ(*C*_*intO*_)⟩. If evaluating complexity *C*_*om*_(|Ψ⟩ or reconstructing geometry from it encodes a halting-type problem, the gravitational features become non-computable. Thus, *f* can be linked to physically plausible non-computable processes within quantum gravity. Postulate 7 (intuitive inference process): The observer possesses a non-algorithmic reasoning process (intuitive inference) utilizing patterns in quantum measurement outcomes (via *f*) and complexity-based geometric cues. This process does not constitute a formal proof but can access truths (like the truth of *S*) that are not provable within *f*. Postulate 8 (formalizing intuitive inference): There exists a non-classical, non-computable logical or probabilistic framework in which the observer’s intuitive inference *I* is represented as: *I*:({*P*(*a*_*i*_|*C*_*on*_,*C*_*intO*_)},*C*_*om*_,*F*,*S*)→{*TruthValues*}. Within this framework, *I* leverages the non-computable structure of *f* and the embedded logical content of *F* to “see” that *S* is true but unprovable in *F*.

Theorem (*N*-Frame non-computable Gödelian truth conjecture): Under Postulates 1–8, given a formal system *F* and a Gödelian statement *S* that is true but unprovable in *F*, and given that the observer’s internal context *C*_*intO*_ encodes *M*,*w* for the Halting Problem, the non-computable function *f*, along with the intuitive inference mechanism *I* and the emergent gravitational complexity structure enables the observer to determine the truth value of *S* without relying on a formal proof in *F*.

Conceptual demonstration Proof (conditional hypothetical derivation with a proof-by-contradiction sub-argument): Step 1: (non-computability of *f*): By construction (Postulate 5), evaluating *f*(ρ,*C*_*on*_,*C*_*intO*_) decides whether *M* halts on *w*. If there were a Turing machine computing *f*, it would decide the Halting Problem. Since the Halting Problem is known to be undecidable, *f* is non-computable. Step 2 (relation to known non-computable functions and complexity classes): The Halting Problem’s non-computability is well-established. Thus, *f* sits outside any computable complexity class, at least as hard as the RE-complete halting set. By substituting variants involving Busy Beaver functions or other known non-computable sets, we can strengthen this conclusion. Therefore, *f* exemplifies a function of Turing degree 0′, beyond the reach of algorithmic methods. Step 3 (physical and geometric interpretation of emergent gravity): By Postulate 6, complexity measures and geometric reconstructions in certain quantum gravitational frameworks (e.g., AdS/CFT) can encode similar halting-type problems. If confirmed, this would show that non-computability is not just a formal artifact but could be inherently tied to fundamental physics. Thus, the observer’s *f*-based inference may reflect physically meaningful constraints, supporting Penrose-like claims of non-computable quantum gravity effects. Step 4 (cognitive modeling and intuitive inference): Postulates 7 and 8 state that the observer’s intuitive inference *I* can use patterns from *f*-influenced measurements to recognize when a Gödelian statement *S* is true but not provable in *F*. Since *f* is non-computable, no process, algorithmic reasoning (like a formal proof in *F*) can replicate this insight. The observer’s inference corresponds to a form of reasoning that is not encapsulated by any finite, computable procedure. Step 5 (truth without proof): Since *S* is unprovable in *F*, standard derivations fail. However, by coupling the logical structure of *F* embedded in *C*_*intO*_ with the non-computability of *f*, the observer can “intuit” that any attempt to prove *S* in *F* fails, yet *S* must be true. This meets the goal: accessing “truth without proof.” All postulates and steps together show that the non-computable function *f* provides the observer with a channel of reasoning that surpasses the Gödelian limitations of formal proof systems. The interplay of complexity theory, quantum gravity hypotheses, and cognitive inference within the *N*-Frame framework suggests a scenario where an observer can determine the truth of a Gödelian statement *S* through non-computable processes, thereby achieving what no formal proof can secure.

It should be noted that postulate 6 can be extended, i.e., in an AdS/CFT duality scenario, bulk geometry is related to computational complexity and entanglement patterns in boundary states |Ψ(*C*_*intO*_)⟩. If evaluating complexity *C*_*om*_(|Ψ⟩) or reconstructing geometry from it requires solving a halting-type problem, then the gravitational features of the bulk become non-computable. Since observer-dependent collapse (modeled via $-\hat{\Gamma }$) modifies the measurement framework, and complexity growth in the boundary theory is linked to bulk evolution, the observer’s choice of measurement operators *M_i_* influences non-computable features of the bulk. Thus, *f*, the observer’s cognitive inference function, is not only non-computable but holographically realized through complexity evolution in quantum gravity.

Unlike the Penrose approach of orchestrated objective reduction, this *N*-Frame (Edwards, 2023, 2024) approach provides a very detailed account of how truth may be obtained without proof. However, non-computability cannot be experimentally verified by any finite procedure, and therefore verifying a putative Halting oracle is a logical catch-22. To prove something truly solves the Halting Problem or produces a non-computable sequence, this would need to be checked against every possible Turing machine and input, i.e., an infinite check. In practice, it is not possible to confirm in finite time that no algorithm replicates a device’s outputs or that it can solve all Halting instances. As these *N*-Frame postulates are intrinsically beyond finite verifiability, so it cannot be “proven” like a standard theorem or tested like a standard physics hypothesis (a computational limitation of the system or universe in which we as observers *C*_*intO*_ exist in, i.e., an absolute epistemic limit of the system itself). The proof provided is a conditional derivation (a conceptual demonstration) as it demonstrates that under these assumptions (which themselves are non-finitely verifiable), the conclusion follows. This does not contradict the fact that non-computability (and thus a true Halting oracle) cannot be proven by any finite procedure, because the argument itself operates at a meta-level that acknowledges those intrinsic limitations. It shows that if we accept the postulates, each of which involves non-computable elements and assumptions that extend beyond finite verification, then the observer’s cognitive inference can access truths that are unprovable within the original system.

Experimental evidence can, however, be obtained via modeling experimental evidence in the known psychological domains that have applied a QM framework to explain their findings such as QPT (Pothos and Busemeyer, 2022) and Raiden-type consciousness causes collapse (or perturbation) findings (Baer, 2015; Bierman, 2003; Bösch et al., 2006; Ibison and Jeffers, 1998; Radin, 2008; Radin and Delorme, 2021; Radin et al., 2015a; Radin et al., 2016; Radin et al., 2012; Radin et al., 2013; Radin et al., 2015b; Radin et al., 2019; Vieten et al., 2018), whilst accounting for the measurement problem. This would need to be formalized via proof, why a model of QM applied to psychological findings would need to account for the measurement problem through structural and functional equivalence. So, although a definitive “proof” of the *N*-Frame non-computable Gödelian argument can not possible be found, the structured argument can serve to help form experiments or interpretive checks of the framework. In other words, even though we cannot finitely verify with formal mathematical proof “beyond computation (or hypercomputation)” we can still examine whether the *N*-Frame approach models known phenomena that apply a QM framework such as cognitive bias expanding QPT and consciousness causing (or actualizing) collapse of the waveform, hence accounting for the measurement problem. From this we can then suggest additional experiments to verify this approach and conceptual realigning of QM with an observer interface as described by *N*-Frame (Edwards, 2023, 2024).

### 6.1 N-Frame application to known psychological results that employ QM

Quantum cognition applies mathematical principles from quantum mechanics to model cognitive processes and decision-making. Traditional probabilistic Bayesian models often fail to explain paradoxical human behaviors observed in cognitive psychology, such as the conjunction fallacy, disjunction effect, and order effects. QPT (Pothos and Busemeyer, 2022) with its features of superposition, interference, and contextuality, offers a potential framework to model these phenomena. The Linda problem, introduced by Tversky and Kahneman (1983), is a classic example of the conjunction fallacy. Participants are given a description of Linda, a young woman deeply concerned with social justice issues. They are asked to rank the likelihood of statements such as: Statement A: “Linda is a bank teller.” Statement B: “Linda is a bank teller and is active in the feminist movement.” Findings of these studies tend to show that the majority of participants rate Statement B as more probable than Statement A, violating classical probability rules since *P*(*AB*)≤*P*(*A*) (a probability inequality whereby answer A is more likely to be given than the probability of A and B both happening).

Several prominent Physicists have long suggested that understanding consciousness may give a deeper understanding of physical phenomena as they relate to quantum theory, such as described by Wolfgang Paulie in *The Interpretation of Nature and the Psyche* (Jung and Pauli, 1955). Quantum Probability theory (QPT) does not suggest that the cognitive system is a quantum mind or quantum brain, instead, it only focuses on applying the mathematics of QM (QM probability) in a way that is inherently useful for making calculations about certain cognitive outcomes. This conservative approach may be useful as a starting position but is perhaps less fruitful in the long term. This conservative perspective is also synonymous with the “shut up and calculate” approach often taken in QM physics attributed to David Mermin (1989), who highlighted what is wrong with physics. Mermin used the phrase “shut up and calculate” to highlight a broader issue within the field of QM, i.e., the divide between pragmatic calculation and philosophical interpretation. Mermin acknowledged that many physicists adopt this pragmatic (QM as a calculation tool) attitude to sidestep and avoid deeper philosophical questions about the nature of reality in quantum mechanics, which may be ontologically uncomfortable and difficult to interpret. However, this avoidance stifles scientific progress in the long term. There may also be a requirement from a structural and functional equivalence argument that QPT and other psychological findings that employ QM should consider the measurement problem rather than just using QM as a predictive tool to model their findings.

Structural equivalence refers to the similarity in the underlying framework of two systems. This encompasses the alignment of fundamental principles or axioms, ensuring that the mathematical constructs, such as Hilbert spaces and operators, and their interrelationships are analogous. Structural equivalence emphasizes the formal, mathematical aspects of systems, establishing a one-to-one correspondence (mapping) that preserves structural relations. For instance, in a theorem, structural equivalence can be demonstrated by mapping the state vectors, superposition principles, measurement-induced collapse mechanisms, and non-commutative operators of QM to their counterparts in QC. In contrast, functional equivalence pertains to the similarity in behavior or functionality between two systems. This includes operational similarity, where systems perform analogous functions or operations, and outcome parity, where they (QM and QC) produce similar results under comparable conditions. Functional equivalence ensures that the systems can be applied to solve similar problems or model similar phenomena effectively. An example of functional equivalence in a theorem can be found in the modeling of cognitive biases like the conjunction fallacy or order effects in surveys using QPT, which mirrors how QM explains similar phenomena through quantum interference and non-commutativity.

Note that in any account of QM in the cognitive system this inherently includes an observer *C*_*intO*_ (the individual making decisions or judgments). This makes the system open, as it interacts with internal processes (thoughts, emotions) and external stimuli (questions, tasks). This means that the dynamics of cognitive processes often involve discrete transitions (e.g., making a decision) rather than continuous evolution. These transitions may be non-unitary, involving processes like forgetting, belief updating, or contextual influences, which do not preserve the norm of the state vector in the same way as unitary evolution in QM. This would be the same for a QM system to be repeatedly measured by an observer, the system would then become open and discrete (non-continuous).

### 6.2 Structural and functional equivalence theorem of QM and QPT

Abstract quantum-theoretic framework (AQF) Axioms is a general mathematical and conceptual framework that encompasses the core formalism of quantum theory, stripped of any specific interpretation (e.g., physical, cognitive, or otherwise). The idea behind the AQF is to distill the essential mathematical structure that defines quantum theory, so it can be applied not only to physical systems but also to other domains like cognition, decision-making, and information theory. The AQF provides a set of axioms that define the structure of quantum systems in terms of state spaces, observables, probabilities, and dynamics. These axioms are abstract in the sense that they do not assume any specific domain of application (e.g., particles, spins, or human cognition) but instead define the general properties that a “quantum-like” system must satisfy. We assume both QM and QPT fit into the following abstract framework: Axiom 1 (state space): The system (physical or cognitive) is represented by a separable Hilbert space *H*. All states are density operators ρ on *H*. This ensures a common state-space structure for QM and QPT. Axiom 2 (observables and algebra): The observables form a non-commutative C*-algebra *A* ⊆ *B*(*H*). Self-adjoint elements in *A* represent observables. Both QM and QPT share this algebraic setup. Axiom 3 (superposition): The theory allows superpositions of states. Any linear combination of pure states (and by extension any convex combination of density operators) is permitted. This ensures linearity in both QM and QPT. Axiom 4 (Born rule): The probability of an outcome associated with a projector *P* ∈ *A* from a state ρ is Tr(ρ*P*). This probabilistic postulate is identical in QM and QPT. Axiom 5 (Dynamics): Time evolution is governed by either a strongly continuous one-parameter unitary group *U*(*t*) = *e*^−*iHt*/ℏ^ for some self-adjoint *H* ∈ *A*, or more general CPTP maps. QM and QPT share this dynamical structure. Theorem (structural and functional foundational equivalence of QM and QPT): Let *H*_*QM*_, *A*_*QM*_ and ρ_*QM*_ be a quantum mechanical system (*H*_*QM*_ is the Hilbert space associated with a quantum mechanical system, *A*_*QM*_ is the algebra of observables associated with *H*_*QM*_ forming a non-commutative C*-algebra, and ρ_*QM*_ is a density operator on *H*_*QM*_ representing a quantum state in QM), and *H*_*cog*_, *A*_*cog*_, and ρ_*cog*_ be a quantum probabilistic cognitive system (*H*_*cog*_ is the Hilbert space associated with a quantum probabilistic cognitive system, *A*_*cog*_ is the algebra of cognitive observables, also forming a non-commutative C*-algebra, and ρ_*cog*_ is a density operator on *H*_*cog*_, representing a cognitive state) each satisfying Axioms 1–5 of the abstract quantum-theoretic framework (AQF). Assume: (1) *A*_*QM*_ and *A*_*cog*_ are *-isomorphic C*-algebras. This is, there exists a *-isomorphic Φ: *A*_*QM*_ → *A*_*cog*_. (2) dim(*H*_*QM*_) = dim(*H*_*cog*_). There exists a unitary operator *U*:*H*_*QM*_ → *H*_*cog*_ such that for all *A*_*QM*_:Φ(*A*) = *UAU*^†^, and for states ρ_*cog*_ = *U*ρ_*QM*_*U*^†^. Thus, QM and QPT are unitarily equivalent representations of the same abstract quantum structure, establishing a deep foundational equivalence, and confirming both their structural and functional equivalence at a foundational level.

Direct Proof: Step 1 (state spaces): By Axiom 1, QM and QPT each have states represented as density operators on separate Hilbert spaces *H*_*QM*_ and *H*_*cog*_. Given dim(*H*_*QM*_) = dim(*H*_*cog*_), there is a Hilbert space isomorphism *U*_0_:*H*_*QM*_ → *H*_*cog*_. This step is purely linear algebraic: separable infinite-dimensional Hilbert spaces are isomorphic (e.g., both isomorphic to *l*^2^ (ℕ) if infinite-dimensional. Step 2 (observables, -Isomorphism, and Axiom 2): From Axiom 2, both QM and QPT have observables forming C*-algebras *A*_*QM*_ and *A*_*cog*_. The existence of a *-isomorphism Φ:*A*_*QM*_ → *A*_*cog*_ means that Φ preserves the algebraic structure, the *-operations, and the identity. In particular, Φ preserves the spectral structure of self-adjoint elements. Step 3 (superposition): By Axiom 3, superpositions are allowed in both frameworks. Since a unitary operator *U* is linear and bijective, it preserves superpositions and the convex structure of state spaces. Thus, the linear geometry of the state space is unchanged under the isomorphism. Step 4 (Born rule): From Axiom 4, the probability of an outcome defined by a projector *P* ∈ *A*_*QM*_ for a state is Tr(ρ_*QM*_*P*). Define ρ_*cog*_ = *U*ρ_*QM*_*U*^†^. Because Φ(*P*) = *UPU*^†^ and the trace is unitarily invariant: Tr(ρ_*cog*_Φ(*P*)) = Tr(*U*ρ_*QM*_*U*^†^*UPU*^†^) = Tr(ρ_*QM*_*P*). Step 5 (Dynamics): By step 5, time evolution can be described by unitary groups or CPTP maps. Suppose QM evolves by ρ_*QM*_(*t*) = *U*_*QM*_(*t*)ρ_*QM*_(0)*U*_*QM*_(*t*)† with *U*_*QM*_(*t*) = *e*^−*iH*_*QM*_*t*/ℏ^ . Define *H*_*cog*_ = *UH*_*QM*_*U*^†^, so *U*_*cog*_(*t*) = *e*^−*iH*_*cog*_*t*/ℏ^ = *Ue*^−*iH*_*QM*_*t*/ℏ^*U*^†^. Therefore ρ_*cog*_(*t*) = *U*ρ_*QM*_(*t*)*U*^†^ = *U*_*cog*_(*t*)ρ_*cog*_(0)*U*_*cog*_(*t*)†. If the evolution is given by a CPTP map *ℰ*_t_ on QM states, then define ${ℰ}_{t}^{\prime }\,\left(\rho_{c\,o\,g}\,U\right)\,U^{\text{†}}$. This shows that all dynamical processes correspond exactly under the established unitary equivalence, satisfying Axiom 5.

Two main conclusions can be drawn from this: (1) Structural Equivalence (Axioms 1–3): We have shown the existence of a unitary *U* implementing the *-isomorphism Φ. This preserves the entire structure of the Hilbert spaces, the operator algebras, and the superposition principle. Hence, structurally, QM and QPT are identical frameworks. (2) Functional Equivalence (Axioms 4–5): With the Born rule probabilities and the dynamics also preserved under the unitary equivalence, all operational predictions (probabilities, expectation values, temporal evolutions) are identical between QM and QPT. Thus, functionally, the two frameworks are also equivalent. In other words, any predictive or descriptive power that QM has is mirrored by QPT, and vice versa, once the correct unitary and -isomorphism are established.

This structural and functional equivalence theorem of QM and QPT has foundational implications. It shows that once you abstract away from a physical interpretation and focus solely on the mathematical structure, Hilbert spaces, C*-algebras, states, observables, and the Born rule, there is a unique formal framework underlying both physical quantum mechanics and quantum-inspired cognitive models. Such a theorem helps clarify what is “quantum” about QPT and ensures that no hidden assumptions distinguish the two domains at the mathematical level. It is indeed needed to move beyond informal analogies and demonstrate a robust, mathematically grounded equivalence between QM and QPT.

In other words, while QM’s measurement problem is about how nature itself chooses outcomes from superpositions, QPT’s “measurement” applied to cognition is often about how a cognitive system transitions from uncertainty or ambiguity to a definite response (i.e., making a decision). This can be given psychological, computational, or decision-theoretic interpretations, rather than demanding a specific ontological resolution akin to the physics measurement problem.

In standard QM, the measurement problem arises from the attempt to reconcile the unitary, deterministic evolution of the wavefunction (as governed by the Schrödinger equation) with the apparently non-unitary “collapse” that occurs during a measurement. Physically, this leads to interpretational challenges about how, when, and why a quantum system transitions from a superposition state to a definite outcome. QPT (Pothos and Busemeyer, 2022) as a modeling framework applied to cognition uses the mathematical apparatus of QM, Hilbert spaces, state vectors (or density operators), and non-commutative observables to model cognitive phenomena such as decision-making, conceptual combination, or order effects in judgments. However, QPT typically treats “measurement” as a metaphor for cognitive events like making a choice, reporting a judgment, or arriving at a belief. In this sense, “measurement” is not a mysterious physical process but rather a conceptual operation, i.e., the act of eliciting or actualizing a single cognitive outcome from multiple potential (superposed) mental states. For this reason, the act of measurement in QPT models of decision making should be described ontologically rather than just treating QM as a computational tool to predict cognitive outcomes. Given the structural and functional equivalence between QM and QPT, the measurement problem in QM naturally extends to QPT, necessitating a deeper examination of its implications in cognitive models. The mantra “shut up and calculate” simply will not do in this context because measurement collapse, a fundamental issue in QM, must also be accounted for in QPT. If QPT is truly equivalent to QM at a foundational level, then it cannot merely borrow QM’s predictive power, it must also grapple with the conceptual challenges posed by the measurement process itself.

When applying this *N*-Frame (Edwards, 2023, 2024) model to the Linda problem, we need to consider three possible cognitive states in the Linda problem, participants are presented with a description of Linda and asked to assess the likelihood of certain statements about her. The key empirical findings are: Probability that Linda is a bank teller *P*(*T*) approximately 15% probability that Linda is a bank teller and active in the feminist movement *P*(*T*∧*F*) approximately 85% probability that Linda is active in the feminist movement but not a bank. These probabilities reflect the conjunction fallacy, where *P*(*T*∧*F*) > *P*(*T*), which contradicts classical probability *P*(*T*∧*F*)≤*P*(*T*) and *P*(*T*∧*F*)≤*P*(*F*) as they are subsets therefore less likely. This phenomenon suggests that participants use heuristic reasoning, influenced by Linda’s description, rather than adhering to formal probability rules. The conjunction fallacy occurs when individuals incorrectly judge the probability of a conjunction of two events *A* and *B*, *P*(*AB*) to be higher than the probability of one of the events alone *P*(*A*) or *P*(*B*), violating the conjunction rule of classical probability *P*(*AB*) < min{*P*(*A*),*P*(*B*)}.

The description given about Linda is that she is 31 years old, single, outspoken, and very bright. She majored in philosophy. As a student, she was deeply concerned with issues of discrimination and social justice and also participated in anti-nuclear demonstrations. Participants are asked to rank the likelihood of several statements about Linda, including *T* (Linda is a bank teller), *T*&*F*: (Linda is a bank teller and is active in the feminist movement). Empirically, many participants rate *T*&*F* as more probable than *T* *P*(*T*∧*F*) > *P*(*T*) exhibiting the conjunction fallacy.

Traditional Bayesian probability theory cannot account for this fallacy but QPT (Pothos and Busemeyer, 2022) can and is uses the mathematical formalism of quantum mechanics to model cognitive processes, incorporating superposition and interference effects. In QPT (Pothos and Busemeyer, 2022), cognitive states are represented as vectors in a complex Hilbert space. The state of a participant’s belief system is described by a state vector |ψ⟩. Events are represented by projection operators onto subspaces corresponding to those events. From this theory, cognitive states can be described as in a superposition of different belief states, and the probability of the events can be affected by constructive or destructive interference between cognitive states. For the Linda problem, cognitive states |ψ⟩ can be represented as |ψ⟩ = cosθ|*B*⟩ + sinθ|*F*⟩, whereby |*B*⟩ states represents “Linda is a bank teller,” and |*F*⟩ state represents “Linda is a feminist.” The angle θ captures the influence of Linda’s description on the participant’s belief state. The events are represented by projection operators, for event *T* can be denoted as ${\hat{P}}_{T}=\left|B\right\rangle \,\left\langle B\right|$, and for event *T*&*F* we can define a new state |*B*_*F*_⟩ representing the conjunction |*B*_*F*_⟩ = cos*ϕ*|*B*⟩ + sin*ϕ*|*F*⟩. The angle *ϕ* captures the overlap between the conjunction state and the basis states, and the projection operator for *T*&*F* is ${\hat{P}}_{T\&F}=\left|B_{F}\right\rangle \,\left\langle B_{F}\right|$. In order to calculate probabilities such as *T* can be denoted as $P\left(T\right)=\left\langle \psi |{\hat{P}}_{T}|\psi \right\rangle =|\left\langle \text{B}|\psi \right\rangle {|}^{2}={\text{cos}}^{2}\theta$. The probability of *T*&*F* can be denoted as *P*(*T*&*F*) = |⟨*B*_*F*_|ψ⟩|^2^ = |cos*ϕ*cosθ + sin*ϕ*sinθ|^2^ = cos^2^(θ−*ϕ*). This result arises because |*B*_*F*_⟩ is a linear combination of |*B*⟩ and |*F*⟩, and the inner product simplifies accordingly. For the parameter selection and interference, to model the conjunction fallacy *P*(*T*&*F*) > *P*(*T*), we need cos^2^(θ−*ϕ*) > cos^2^θ. This occurs when |θ−*ϕ*| < θ, meaning that *ϕ* is close to θ. For the interference parameter, the difference θ−*ϕ* represents the interference effect between the cognitive states. A smaller difference leads to constructive interference, increasing *P*(*T*&*F*). For example, let us choose θ = 60(θ = π/3*radians*), *ϕ* = 50(θ = 5π/18*radians*, the calculations are $P\,\left(T\right)={\text{cos}}^{2}\,\theta ={\text{cos}}^{2}\,\left(\frac{\pi }{3}\right)={\left(\frac{1}{2}\right)}^{2}=0.25\,\left(25\%\right)$. $P\,\left(T\&F\right)={\text{cos}}^{2}\,\left(\theta -\phi \right)={\text{cos}}^{2}\,\left(\frac{\pi }{3}-\frac{5\,\pi }{18}\right)={\text{cos}}^{2}\,\left(\frac{\pi }{18}\right)\approx {\text{cos}}^{2}\,\left(10\right)\approx {\left(0.9848\right)}^{2}\approx 0.9698\,\left(96.98\%\right)$. While these numbers are mathematically correct given the chosen parameters, they differ from typical a little form empirical estimates [which are around 15% for *P*(*T*) and 85% for *P*(*T*&*F*)]. The specific values of θ and *ϕ* can be adjusted to better match empirical data, but the key point is that the framework captures the essential interference effect leading to *P*(*T*&*F*) > *P*(*T*).

Another account can be given by *N*-Frame (Edwards, 2023, 2024) which is an evolutionary and predictive coding model, as well as an observer-centric quantum framework. From this evolutionary perspective, what appears to be a cognitive bias or an “error” such as the conjunction fallacy may actually reflect a sophisticated cognitive adaptation in how humans process and integrate information. Traditional probability theory deems it irrational for people to rank “feminist and bank teller” as more likely than “bank teller” alone when judging Linda. However, considering cognition as shaped by evolutionary forces reveals that this seemingly illogical judgment may confer real-world advantages. The human brain has not evolved to follow classical probability axioms in many cases; rather, it has been honed to quickly interpret complex, socially relevant cues under uncertainty (as this promotes survival at the evolutionary level). This evolutionary perspective helps explain why human intuition and reasoning can transcend the limits of strict logical frameworks, including those highlighted by Gödel’s incompleteness. This evolutionary tuning favors configurations of belief states, akin to stable “eigenforms” of cognition, that minimize risks and errors in predicting the behaviors of others. In this view, the *T*∧*F* state in the Linda problem emerges not merely as an interference pattern explained by quantum probability, but also as a low free-energy form of cognition consistent with predictive coding. Such a form of cognition bundles multiple correlated traits (e.g., social activism, nuclear protests, and feminist orientation) into a robust predictive model, enabling an observer to rely on feature redundancy for more reliable judgments i.e., the observer *C*_*intO*_ such as a person making a decision uses overlapping or correlated features in the information they receive to reinforce their judgment and reduce uncertainty in their decision-making. Over ancestral environments, individuals whose cognitive processes favored these stable, resilient categorization patterns likely had better social navigation skills and survival rates. The evolutionary dimension thus reframes the conjunction fallacy as a cognitive adaptive strategy, one that, while not strictly “rational” in mathematical terms, is pragmatically beneficial for organisms navigating a complex and uncertain world.

Below is a worked example illustrating how one might *N*-Frame (Edwards, 2023, 2024) integrate perturbation and collapse (along with low free energy predictive contextual factors) model with a QPT-based approach to model the Linda problem. Perturbation, as well as collapse, are conceptual tools that build upon the underlying quantum framework, which could be standard QM or QPT, to incorporate consciousness and context. The perturbation approach does not replace QM or QPT; rather, it adds to it (in this way *N*-Frame can be seen as a larger model of existing QM and QPT approaches). Perturbation is a technique commonly used in physics, starting from a known solution of a system and then adding a small correction (λ is small). Unlike standard QPT, which selects θ and φ to fit data (which is a potential criticism of the approach), *N*-Frame derives these values from first principles, as perturbation adjusts them dynamically based on context, while collapse stabilizes them as evolutionarily favored cognitive structures.

In the *N*-Frame (Edwards, 2023, 2024) context, the perturbation can represent the conscious observer’s focus (i.e., the observer *C*_*intO*_ is the measuring device, in line with structural and functional equivalence’s conclusion that the QM model describing cognition should account for the measurement problem, and *N*-Frame does) as a slight modification to operators or states, resulting in probability shifts. Without the underlying quantum framework (standard QM or QPT), perturbation alone has no formal meaning. It is a correction term applied to an existing quantum model. Similarly, introducing a collapse model to the *N*-Frame account (such as adding non-Hermitian terms) also relies on the foundational quantum structure. The collapse model can be thought of as another extension or layer that gives you a mechanism to select stable “eigenforms” of cognition over time. Without a quantum probability backbone, the concept of collapse dynamics wouldn’t have a formal substrate on which to act. This is important for a universal model, as the model needs to explain why we see definite states not superposition states., and only a collapse process can explain this (or perhaps a “many possible world” approach). QPT already includes a form of collapse via its use of projection operators. When a quantum probability model is applied to cognition, measurement (i.e., making a judgment or decision) is represented mathematically by a projection operator acting on the cognitive state |ψ⟩. This process collapses the superposition into a particular outcome, just as in standard quantum mechanics, but the superposition are mental states (decisions) and not physical aspects of the world. *N*-Frame (Edwards, 2023, 2024) therefore provides a natural extension to this QPT model rather than as its replacement.

We start with QPT to represent the cognitive state and events. Suppose the observer’s belief about Linda is represented by a state |ψ⟩ in a two-dimensional Hilbert space spanned by |*B*⟩ (Linda is a Bank Teller) and |*F*⟩ (Linda is a Feminist). Initially, we might write: |ψ⟩ = cosθ|*B*⟩ + sinθ|*F*⟩. Here, θ parameterizes the subjective weight given to the “bank teller” vs. “feminist” attributes. For the conjunction, we define: |*B*_*F*_⟩ = *cos*⁡φ|*B*⟩ + sinφ|*F*⟩, representing the combined concept of feminist *F* and bank teller *BT* concept. The probability of selecting his conjunction is *P*(*T*∧*F*) = |⟨*B*_*F*_|ψ⟩|^2^ = *cos*^2^(θ−φ). In pure QPT, choosing φ close to θ creates a constructive interference such that *P*(*T*∧*F*) = *P*(*T*) = cos^2^θ, modeling the conjunction fallacy as an interference phenomenon.

However, perturbations of *N*-Frame (Edwards, 2023, 2024) can be introduced when we recognize that participants’ cognitive states are not fixed, but are instead influenced by context. Subtle contextual hints in Linda’s description (her activism, philosophy background, concern about social issues) can slightly adjust the angles θ and φ. QPT inherently models context dependence via superposition and interference. A participant’s belief state |ψ⟩ = cosθ|*B*⟩ + sinθ|*F*⟩ already reflects their prior cognitive bias. The probability of conjunction *P*(*T*∧*F*) is influenced by the relative phase and overlap between the belief states |*B*⟩ and |*F*⟩, allowing contextual factors to affect outcomes via quantum interference. However, QPT does not specify how or why the angles θ and φ shift based on contextual details. It provides a mathematical framework but does not derive these shifts dynamically. *N*-Frame perturbation models this process explicitly by incorporating conscious focus *C*_*intO*_ and low free-energy predictive adjustments as additional perturbative terms. Instead of assuming θ and φ shift arbitrarily, *N*-Frame derives them based on an observer’s intentional focus and predictive priors, modifying probability amplitudes in a principled way, and consistent with accounting for the measurement problem (consciousness) that the structural and functional equivalence proof forces us to adopt. Therefore, QPT provides the structure, but *N*-Frame explains the dynamics of belief updating. Traditional QPT assumes contextual influence implicitly through initial state preparation or interference, whereas *N*-Frame explicitly models context as a perturbative shift in the cognitive state due to attention and focus. This makes it possible to integrate predictive coding processes, where the observer’s priors (based on evolutionary heuristics) guide how much certain aspects of Linda’s description shift their cognitive state representation (and this could also potentially be modeled over time with more dynamic evolutions of the decision making process).

Introducing a small perturbation δθ can shift θ depending on context. For example, suppose the participant initially has θ = 60. Without perturbation, *P*(*T*) = cos^2^(60) = 0.25. However, if the description of Linda’s activism leads the participant to place more weight on “feminist,” their internal processes may nudge θ upward by a small amount δθ = 5. After perturbation, θ′ = θ + δθ = 60 + 5 = 65. Similarly, the participant may also adjust φ slightly φ′ = φ + δφto maintain consistency between the idea of conjunction and their evolving internal narrative. If initially φ = 50, a slight shift due to context might make φ′ = 52. Both of these perturbations increase the probability of *P*(*T*∧*F*) > *P*(*T*), strengthening the constructive interference effect already identified by QPT. So, while *P*(*T*∧*F*) slightly decreases, *P*(*T*) decreases more significantly, reinforcing the observed cognitive bias.

Specifically, the incorporation of the conscious-influence function of *N*-Frame (Edwards, 2023, 2024) (for perturbation or collapse) $g\,\left|\psi_{\text{intO}}^{\text{focus}}\right\rangle$. We stated that $g|\psi_{\text{intO}}^{\text{focus}}\rangle =1+\eta |\langle |\psi_{i\,\text{ntO}}^{\text{focus}}|\psi_{i\,n\,t\,O}\left(0\right)\rangle |2$, where η is a small parameter quantifying the strength of conscious influence. Without angle changes, the modified probability for an event *a_i_* is: $P\left(a_{i},t\right)=P_{s\,t\,a\,n\,d\,a\,r\,d}\left(a_{i},t\right)[1+\eta |\left\langle |\psi_{i\,\text{ntO}}^{\text{focus}}|\psi_{i\,n\,t\,O}\left(0\right)\right\rangle {|}^{2}$. This factor multiplies the standard QM probability. When η = 0, there is no conscious effect. η is introduced as a small parameter within the function $g\,\left|\psi_{\text{intO}}^{\text{focus}}\right\rangle$. They both represent the influence of consciousness on the probability, but at different levels of detail. The function *g*(⋅) is a general expression telling us how much the standard probability is modified by the observer’s focused intent. The parameter η is introduced as a way to linearize and quantify that influence as a small perturbation. In other words, *g*(⋅) is the overall “consciousness factor,” and η is the specific small parameter we choose to represent the strength of this conscious effect within *g*(⋅).

We next show how to combine angle changes and conscious influence. We want a single expression that shows how both angle changes and conscious influence affect the outcome. Consider *P*(*T*∧F) again. With both effects, we get: (1) Angle perturbation changes *P*_*standard*_(*T*∧F) to *P*(*T*∧F)′, as derived: *P*(*T*∧F)′≈*P*_*standard*_(*T*∧F)−*sin*⁡(2Δ)(δθ−δφ). (2) Conscious influence multiplies this perturbed probability by $[1+\eta |\left\langle |\psi_{i\,\text{ntO}}^{\text{focus}}|\psi_{intO)}\left(0\right)\right\rangle {|}^{2}$. Thus, the final combined probability becomes: $P_{c\,o\,m\,b\,i\,n\,e\,d}\left(T\wedge \text{F}\right)\approx \left[P_{s\,t\,a\,n\,d\,a\,r\,d}\left(T\wedge \text{F}\right)-\sin\left(2\Delta \right)\left(\delta \theta -\delta \varphi \right)\right]\cdot [1+\eta |\psi_{i\,\text{ntO}}^{\text{focus}}|\psi_{i\,n\,t\,O}\left(0\right)\langle {|}^{2}$. Expanding this to first order in small quantities δθ,δφ,η: $P_{c\,o\,m\,b\,i\,n\,e\,d}\left(T\wedge \text{F}\right)\approx P_{s\,t\,a\,n\,d\,a\,r\,d}\left(T\wedge \text{F}\right)+P_{s\,t\,a\,n\,d\,a\,r\,d}\left(T\wedge \text{F}\right)\eta |\left\langle |\psi_{i\,\text{ntO}}^{\text{focus}}|\psi_{i\,n\,t\,O}\left(0\right)\right\rangle {|}^{2}-\sin\left(2\Delta \right)\left(\delta \theta -\delta \varphi \right)+O\left(\eta \delta \theta ,\eta \delta \varphi \right)$, where higher-order mixed terms are even smaller. This final form shows how a very specific change in angles δθ and δφ can work alongside the conscious factor η. If you choose δθ and δφ so that Δ′ = Δ + (δθ−δφ) is significantly reduced, the −*sin*⁡(2Δ)(δθ−δφ) can be large and positive, subsequently boosting *P*(*T*∧F) as a more likely decision. Simultaneously, the conscious factor η adds another multiplicative boost. This derivation is both logically consistent and mathematically accurate under the assumption that δθ, δφ, and η are small quantities. The final expression $P_{c\,o\,m\,b\,i\,n\,e\,d}\left(T\wedge \text{F}\right)\left[P_{s\,t\,a\,n\,d\,a\,r\,d}\approx \left(T\wedge \text{F}\right)-\sin\left(2\Delta \right)\left(\delta \theta -\delta \varphi \right)\right]\cdot \left[1+\eta |\psi_{i\,\text{ntO}}^{\text{focus}}|\psi_{i\,n\,t\,O}\left(0\right)\right\rangle {|}^{2}$ captures how both the perturbative angle changes and the conscious influence modify the probability of the conjunction event, reinforcing the conjunction fallacy in a principled way. If the net effect of the angle perturbations is to reduce the effective difference (i.e. if δθ−δφ is negative enough to make −*sin*⁡(2Δ)(δθ−δφ) a positive contribution), then *P*(*T*∧F) is boosted relative to *P*_*standard*_(*T*∧F). Simultaneously, the conscious factor (via η) adds an additional multiplicative boost.

As a specific example, let us start with θ = 60, φ = 50, so that the initial difference is Δ = 10. The standard probability is *P*_*standard*_(*T*∧F) = cos^2^(10)≈0.97. Suppose that, due to contextual influences, the participant’s cognitive state shifts such that θ increases by a small perturbation δθ = 5 (from *60* to *65*) and φ similarly increases by δφ = 2 (from *50* to *52*). Then the new effective difference is Δ′ = 10 + (5−2) = 13, which changes the standard probability to *P*(T∧F)′ = cos^2^(13°)≈0.949. While this represents a slight decrease in the absolute probability for the conjunction, note that the probability for the single event *T* (bank teller) decreases more substantially (from *P*(*T*) = cos^2^(60) = 0.25 to *P*(*T*) = cos^2^(65°)≈0.18). This relative change increases the ratio *P*(*T*∧F)/*P*(*T*), thereby reinforcing the conjunction fallacy. These parameters are set by the researcher to fit the data in QPT, so alone does not provide a direct, prescriptive method for choosing θ and φ, it is simply an arbitrary data fitting exercise.

*N*-Frame (Edwards, 2023, 2024), on the other hand, has a precis, a-priori approach to select the conscious influence parameter η, as it is based on the conscious focus of the observer. For instance, by adding the conscious factor η, say for a small influence η = 0.01, and if $|\left\langle |\psi_{i\,\text{ntO}}^{\text{focus}}|\psi_{i\,n\,t\,O}\left(0\right)\right\rangle {|}^{2}=0.5$, then the consciousness-modifying factor is given by $g=1+\eta \cdot |\left\langle |\psi_{i\,\text{ntO}}^{\text{focus}}|\psi_{i\,n\,t\,O}\left(0\right)\right\rangle {|}^{2}=1+0.01\times 0.5=1.005$. Multiplying the perturbed probability by this factor, we obtain *P*_*combined*_(*T*∧F)≈0.949×1.005≈0.953. Thus, the final probability accounts for both the contextual angle shift (which modifies the interference pattern) and the conscious influence factor η, which further enhances the probability of the conjunction event. This demonstrates how consciousness modulates an already interference-sensitive cognitive process.

However, while QPT models and *N*-Frame (Edwards, 2023, 2024) perturbation parameters (such as the conscious influence factor η of *N*-Frame) can both produce the observed patterns, QPT treats its interference parameters as freely adjustable, without an explicit process for conscious modulation which is inherently necessary because the measurement problem carries over to this psychological domain due to the structural and functional equivalence proof. In contrast, *N*-Frame introduces a structured, observer-dependent modification via η, which directly accounts for the influence of conscious focus on probability amplitudes. The *N*-Frame model therefore goes further, providing a dynamical reason why certain configurations become stable or evolutionarily favored over time. This collapse part of *N*-Frame takes the previously introduced angles θ,φand conscious influence parameter η, and gives them a deeper evolutionary meaning. The collapse model makes these “fine-tuned” parameters stable attractors or eigenforms of cognition, suggesting that what might seem arbitrary at first is actually an evolutionarily stable solution that the cognitive system gravitates toward over time.

As a worked example of *N*-Frame (Edwards, 2023, 2024) for the Linda problem, the initial cognitive state is represented as a quantum superposition |ψ(0)⟩ = |ψ_*sys*_(0)⟩⊗ψ_*intO*_(0)⟩, whereby |ψ_*sys*_(0)⟩ = α|*F*⟩ + β|*BT*⟩, |*F*⟩ is a feminist state, and |*BT*⟩ is a Bank Teller state. The coefficients α and β represent the initial amplitudes of each state satisfying normalization |α|^2^ + |β|^2^ = 1. The conjunction state |*F*∧BT⟩ is constructed as a superposition, this is denoted as $\left|F\,\wedge \,\text{BT}\right\rangle =\frac{\left|F\right\rangle +\left|\mathrm{BT}\right\rangle }{\sqrt{2}}$ assuming an equal initial salience for each component. The interaction Hamiltonian ${\hat{H}}_{i\,n\,t}$ encodes the observer’s focus as it selectively amplifies states (cognitive salience) aligned with the observer’s intent ${\hat{A}}_{i\,n\,t\,O}$ and context ${\hat{C}}_{i\,n\,t\,O}$ and this is denoted as ${\hat{H}}_{i\,n\,t}=\lambda_{1}\,\left|F\,\left\langle \right\rangle \,F\right|+\lambda_{2}\,\left|F\,\wedge \,\text{BT}\,\left\langle \right\rangle \,\text{F}\,\wedge \,\mathrm{BT}\right|+\lambda_{3}\,\left|B\,T\,\left\langle \right\rangle \,\mathrm{BT}\right|$, whereby λ_1_ = λ_2_ = λ_3_ reflects a stronger alignment with feminist traits (e.g., social justice, nuclear activism) over the conjunction, and least alignment with the bank teller state. This amplificon of the conjunction state is because the description of Linda promoting social justice such as anti-nuclear demonstrations may be broadly consistent with the social justice beliefs of being a feminist, therefore making these more salient in the focus of the observer ${\hat{H}}_{i\,n\,t}$. It is also important to note that in line with *N*-Frame, the parameters λ_1_,λ_2_λ_3_ encode free energy (surprise) in modeling external “reality,” whereby the brain attempts to minimize errors in its interface prediction (the map) of the external world (the territory). From this free energy minimization perspective, the conjunction state |*F*∧BT⟩ receives constructive interference as the observer’s intent ${\hat{A}}_{i\,n\,t\,O}$ acts linearly on the superposition, distributing across its components such that ${\hat{A}}_{i\,n\,t\,O}\,\left|F\,\wedge \,B\,T\right\rangle =\frac{{\hat{A}}_{i\,n\,t\,O}\,\left|F\right\rangle +{\hat{A}}_{i\,n\,t\,O}\,\left|B\,T\right\rangle }{\sqrt{2}}$, reinforcing the cognitive salience of the conjunction state. The non-Hermitian collapse operator $-i\,\hat{\Gamma }$ introduces irreversibility by (selective) dampening less likely states $\hat{\Gamma }=\gamma_{1}\,\left|B\,T\right\rangle \,\left\langle \mathrm{BT}|+\gamma_{2}|F\,\wedge \,\text{BT}\right\rangle \,\left\langle \text{F}\,\wedge \,\mathrm{BT}|+\gamma_{3}|F\right\rangle \,\left\langle F\right|$, whereby γ_1_ = γ_2_ = γ_3_ ensures faster decay for |*BT*⟩ (high free energy, least congruent), relative to |*F*∧*BT*⟩, and least for |*F*⟩ (low free energy, more congruent). This prunes improbable states, aligning with *N*-Frame’s predictive coding principles.

*N*-Frame (Edwards, 2023, 2024) proposes that eigenforms (stable cognitive patterns) emerge at observer-environment interfaces, encoding evolutionary fitness consequences.

As space and time within this framework are considered components of observational outcomes, then this aligns with other works (Fields et al., 2017; Prakash et al., 2020) that suggest space-time constitutes error-correcting code (e.g., Hamming error correcting) to mitigate perceptual cognitive errors tied to fitness consequences. This perspective integrates Von Foerster’s (2003) eigenform theory with decoherence and holographic encoding, reinforcing the idea that cognition is optimized for survival within an information-bound reality. The error-correcting code introduces redundancy, allowing for the correction of errors within spacetime and further supporting the notion that spacetime itself is fundamentally information-theoretic in nature serving as a framework that optimizes the observer’s decision-making processes. This *N*-Frame interpretation suggests that spacetime itself functions as an adaptive, information-theoretic structure that supports cognition by minimizing errors in perception and decision-making. In this view, spacetime is not merely a passive backdrop but an evolved, error-correcting code that facilitates the observer’s ability to process information effectively. This aligns with the idea that cognition and physics are deeply intertwined, with spacetime acting as a kind of computational substrate optimized for decision-making and evolutionary fitness. As consciousness has a functional role within cognition this means that at an evolutionary the consciousness interface is necessary because it provides a way for an observer to actively engage with and interpret information, facilitating adaptive decision-making.

This suggests that the Linda problem may reveal a deeper evolutionary principle in cognitive processing through the | F∧BT⟩ state’s emergence as a low free energy eigenform. Rather than being a simple cognitive bias or error based on simplistic Bayesian process, the conjunction fallacy might represent an evolutionarily optimized error-correcting process. When presented with Linda’s description (philosophy student, social activism, nuclear protests), the cognitive system naturally evolves toward | F∧BT⟩ as a stable eigenform because it provides redundant error correction through multiple confirming features that mutually reinforce each other. This redundancy in feature-matching serves as a cognitive Hamming code, making the state more robust against errors in prediction and categorization. From an evolutionary fitness perspective, recognizing and predicting stable patterns in social identities and behaviors would have been crucial for survival and social interaction. The | F∧BT⟩ state, despite violating classical probability theory, represents a more reliable predictive model of Linda because it encodes multiple correlated features that have been evolutionarily selected as reliable indicators. This explains why our cognitive systems prefer this lower free energy state, rather than just statistically correlated with the description, but represents an evolved eigenform that optimizes prediction accuracy through built-in error correction. Therefore, the apparent “irrationality” of the conjunction fallacy might actually reflect a sophisticated evolutionary solution to the problem of making robust predictions under uncertainty. As such the total Hamiltonian becomes ${\hat{H}}_{t\,o\,t\,a\,l}=\left({\hat{H}}_{s\,y\,s}-i\,\hat{\Gamma }\right)\,⊗{\hat{I}}_{i\,n\,t\,O}+{\hat{I}}_{s\,y\,s}\,⊗{\hat{H}}_{i\,n\,t\,O}+{\hat{H}}_{i\,n\,t}$, reflecting the non-Hermitian collapse operator $-i\,\hat{\Gamma }$ whereby γ_1_ = γ_2_ = γ_3_ of $-i\,\hat{\Gamma }=-i\,\left(\gamma_{1}\,\left|B\,T\right\rangle \,\left\langle \mathrm{BT}|+\gamma_{2}|F\,\wedge \,\text{BT}\right\rangle \,\left\langle \text{F}\,\wedge \,\mathrm{BT}|+\gamma_{3}|F\right\rangle \,\left\langle F\right|\right)$ ensures faster decay for |*BT*⟩ relative to |*F*∧*BT*⟩, and least for |*F*⟩ because |*BT*⟩ has high free energy whilst |*F*⟩ and |*F*∧*BT*⟩ have low free energy given they are consistent (congruent) with the description Linda is a philosophy major, socially active, nuclear protester, and concerned with discrimination.

The next step in this collapse (irreversible) approach of *N*-Frame (Edwards, 2023, 2024) is modeling the time evolution, according to the Schrödinger equation $i\,\hbar \,\frac{}{t}\,\left|\psi \,\left(t\right)\right\rangle =H_{t\,o\,t\,a\,l}\,\left|\psi \,\left(t\right)\right\rangle$. Expanding |ψ_*sys*_(*t*)⟩ to |ψ_*sys*_(*t*)⟩ = *a*_1_(*t*)|*F*⟩ + *a*_2_(*t*)|*F*∧BT⟩ + *a*_3_(*t*)|*BT*⟨, with time-dependent amplitudes *a*_1_(*t*), *a*_2_(*t*), and *a*_3_(*t*). Then substitute into the equation $i\,\hbar \,\frac{a_{i}\,\left(t\right)}{t}=\left(\lambda_{i}-i\,\gamma_{i}\right)\,a_{i}\,\left(t\right).$The solution for each amplitude is then *a*_*i*_(*t*) = *a*_*i*_(0)*e*^(−*i*λ_*i*_−γ_*i*_)*t*/ℏ^ . The constructive interference for the conjunction state is then $\left|F\,\wedge \,B\,T\right\rangle =\frac{\left|F\right\rangle +\left|\text{BT}\right\rangle }{\sqrt{2}}$. Its amplitude combines contributions from |*F*⟩ and |*BT*⟩ under the observer’s intent ${\hat{A}}_{i\,n\,t\,O}$$a_{2}\,\left(t\right)=\frac{a_{1}\,\left(t\right)+a_{3}\,\left(t\right)}{\sqrt{2}}$. Using the solutions for *a*_1_(*t*) and *a*_3_(*t*), $a_{2}\,\left(t\right)=\frac{a_{1}\,\left(0\right)\,e^{\left(-i\,\lambda_{1}-\gamma_{3}\right)\,t/\hbar }+a_{3}\,\left(0\right)\,e^{\left(-i\,\lambda_{3}-\gamma_{1}\right)\,t/\hbar }}{\sqrt{2}}$. The probability of each state is the modulus squared of its amplitude, so for |*F*∧*BT*⟩, the probability is $P\,\left(F\,\wedge \,B\,T\right)={\left|a_{2}\,\left(t\right)\right|}^{2}={\left|\frac{a_{1}\,\left(0\right)\,e^{\left(-i\,\lambda_{1}-\gamma_{3}\right)\,t/\hbar }+a_{3}\,\left(0\right)\,e^{\left(-i\,\lambda_{3}-\gamma_{1}\right)\,t/\hbar }}{\sqrt{2}}\right|}^{2}$. This shows constructive interference due to the summation of |*F*⟩ and |*BT*⟩. For |*BT*⟩, the probability is *P*(*BT*) = |*a*_3_(*t*)|^2^ − *P*(*F* ∧ *BT*), whereby *P*(*F* ∧ *BT*) subtracts the portion of |*BT*⟩ absorbed into the conjunction. For |*F*⟩, the probability is *P*(*F*) = *a*_1_(*t*)|^2^. We next need to model the observer-driven amplification intent ${\hat{A}}_{i\,n\,t\,O}$ denoted as $a_{1}\,\left(t\right)=\frac{{\hat{A}}_{i\,n\,t\,O}\,a_{1}\,\left(0\right)\,e^{\left(-i\,\lambda_{1}-\gamma_{3}\right)\,t/\hbar }+{\hat{A}}_{i\,n\,t\,O}\,a_{3}\,\left(0\right)\,e^{\left(-i\,\lambda_{3}-\gamma_{1}\right)\,t/\hbar }}{\sqrt{2}}$, whereby ${\hat{A}}_{i\,n\,t\,O}$ enhances |*F*∧*BT*⟩ relative to the other states. The final rankings of the probabilities after sufficient time *t*, can be given by *P*(*F*) remains high due to slower decay γ_3_, *P*(*F*∧*BT*) is amplified by constructive interference and observer focus, *P*(*BT*) decays fastest due to γ_1_ = γ_2_ = γ_3_, leading to the final ranking *P*(*F*) = *P*(*F*∧*BT*) = *P*(*BT*). This description does not contradict the perturbation approach, it complements it. The key difference is that perturbation happens first, subtly shifting probability amplitudes, while collapse follows, irreversibly reinforcing stable eigenforms and eliminating high-entropy states (correspond to more uncertain, less predictable states in cognition), high free energy states (correspond to states with high prediction error, i.e., states that do not align well with the observer’s expectations or prior knowledge).

This *N*-Frame (Edwards, 2023, 2024) approach can be equally applied to the disjunction fallacy and the prisoner’s dilemma, both of which QPT (Pothos and Busemeyer, 2022) also model successfully and more accurately than classical probabilistic (Bayesian) models. For example, in the prisoner’s dilemma, which emphasizes a violation in the sure thing principle represents a failure of classical decision theory. According to classical rationality, players should defect both when they know their opponent will cooperate (to exploit them) and when they know their opponent will defect (to protect themselves). However, paradoxically, under uncertainty about the opponent’s choice, players are more likely to cooperate, contradicting the sure thing principle. Given QPT has successfully modeled this, this phenomenon suggests that human decision-making under uncertainty involves quantum-like interference effects, where the superposition of possible opponent choices creates a decision context that is fundamentally different from classical probability-based reasoning. Just as in the double-slit experiment, where quantum interference produces patterns that cannot be explained by a particle passing through either slit alone, cooperative behavior emerges from the quantum superposition of choices, rather than from a classical mixture of independent states. This quantum perspective explains why uncertainty about another’s decision can fundamentally alter the nature of decision-making, allowing cooperation to arise in conditions where classical rationality would predict defection.

According to *N*-Frame (Edwards, 2023, 2024), this paradoxical behavior arises due to constructive interference of cognitive state that amplifies certain probabilistic pathways, and the impact of observer-driven contextual focus on decision amplitudes (similar to how it models the conjunction fallacy). This aligns with QPT, which models decision-making as a quantum-like process with superposition and interference effects. However, *N*-Frame extends QPT by introducing an explicit process for observer-driven perturbations and collapse, which dynamically shapes the decision landscape based on evolved heuristics. This suggests that the violation of classical rationality principles (like the conjunction fallacy) is not an error, but an adaptive, low-free-energy cognitive process that stabilizes socially advantageous decisions. From this perspective, the interference effects in the superposition state enable cooperative choices that classical decision theory would deem irrational, but which may instead reflect a deeper cognitive eigenform that optimizes long-term social and evolutionary stability.

To show this, the first step is to map this to a quantum framework, whereby the cognitive state of a player before knowing the other player’s decision exists in superposition which includes the observer’s internal state |ψ(0)⟩ = |ψ_*sys*_(0)⟩⊗ψ_*intO*_(0)⟩, whereby |ψ_*sys*_(0)⟩ = α|*A*⟩ + β|*B*⟩ represents the superposition of |*A*⟩ (this represents a cooperation state), |*B*⟩ (this represents a defection state), and the coefficients α and β represent the initial amplitudes of each state satisfying |α|^2^ + |β|^2^ = 1, ensuring normalization. To model constructive interference for cooperation, we introduce a cooperation-defection superposition state |*A*∧*B*⟩ denoted as $\left|A\,\wedge \,B\right\rangle =\frac{\left|A\right\rangle +\left|B\right\rangle }{\sqrt{2}}$, representing a quantum superposition of cooperation and defection amplified by constructive interference. Similar to the conjunction fallacy, the *N*-Frame interaction Hamiltonian ${\hat{H}}_{i\,n\,t}$ encodes the observer’s focus as it selectively amplifies states aligned with the observer’s intent ${\hat{A}}_{i\,n\,t\,O}$ and context ${\hat{C}}_{i\,n\,t\,O}$ and this is denoted as ${\hat{H}}_{i\,n\,t}=\lambda_{1}\,\left|A\right\rangle \,\left\langle A|+\lambda_{2}|A\,\wedge \,B\right\rangle \,\left\langle \text{A}\,\wedge \,B|+\lambda_{3}|B\right\rangle \,\left\langle B\right|$, whereby λ_1_ = λ_2_ = λ_3_ reflects a stronger alignment for cooperation influenced by moral or social norms, and quantum overlap of cooperation and defection that is amplified by constructive interference.

Here, |*A*⟩ may be amplified by moral/social norms favoring cooperation, |*A*∧*B*⟩ moderately amplified by interference, |*B*⟩ least amplified due to its self-interest alignment. This would help to explain why people tend to cooperate more than classical decision theory would predict, especially under uncertainty, whereby λ_1_ > λ_2_ > λ_3_ is consistent with empirical findings. This ordering captures the notion that cooperation is most strongly reinforced by social and moral norms (observer intent), the mixed (conjunction) state benefits moderately from quantum interference, and defection, being aligned with pure self-interest, is least amplified under these conditions. This reflects a deeper, evolutionarily optimized cognitive process rather than a simple error, supporting the idea that the interference effects in the superposition state enable cooperation as an adaptive, low free-energy eigenform.

The non-Hermitian collapse operator of *N*-Frame (Edwards, 2023, 2024) then introduces $-i\,\hat{\Gamma }$ irreversibility by dampening less likely states $\hat{\Gamma }=\gamma_{1}\,\left|B\right\rangle \,\left\langle B|+\gamma_{2}|A\,\wedge \,B\right\rangle \,\left\langle A\,\wedge \,B|+\gamma_{3}|A\right\rangle \,\left\langle A\right|$, whereby this ensures the defection |*B*⟩ decays faster than cooperation |*A*⟩ or the cooperation-defection superposition |*A*∧*B*⟩. The cognitive state then evolves under the total Hamiltonian which incorporates the systems dynamics with collapse $\left({\hat{H}}_{s\,y\,s}-i\,\hat{\Gamma }\right)\,⊗{\hat{I}}_{i\,n\,t\,O}$, the observer’s internal state evolution ${\hat{I}}_{s\,y\,s}\,⊗{\hat{H}}_{i\,n\,t\,O}$ and the interaction Hamiltonian ${\hat{H}}_{i\,n\,t}$ giving a total Hamiltonian ${\hat{H}}_{t\,o\,t\,a\,l}=\left({\hat{H}}_{s\,y\,s}-i\,\hat{\Gamma }\right)\,⊗{\hat{I}}_{i\,n\,t\,O}+{\hat{I}}_{s\,y\,s}\,⊗{\hat{H}}_{i\,n\,t\,O}+{\hat{H}}_{i\,n\,t}$. The time evolution of the cognitive state evolves according to the Schrödinger equation $i\,\hbar \,\frac{}{t}\,\left|\psi \,\left(t\right)\right\rangle ={\hat{H}}_{t\,o\,t\,a\,l}\,\left|\psi \,\left(t\right)\right\rangle$. Expanding |ψ_*sys*_(*t*)⟩ to |ψ_*sys*_(*t*)⟩ = *a*_1_(*t*)|*A*⟩ + *a*_2_(*t*)|*A*∧B⟩ + *a*_3_(*t*)|*B*⟩, where *a*_1_(*t*), *a*_2_(*t*), *a*_3_(*t*) are time-dependent amplitude for |*A*⟩ cooperation, |*A*∧*B*⟩ cooperation-defection superposition, and |*B*⟩ defection.

The next stage is to perform coupled differential equations, for each state |*i*⟩, the amplitudes evolve as the equation $i\,\hbar \,\frac{a_{i}\,\left(t\right)}{t}=\left(\lambda_{i}-i\,\gamma_{i}\right)\,a_{i}\,\left(t\right)$, with the solution for each amplitude is then $a_{i}\,\left(t\right)=a_{i}\,\left(0\right)\,e^{\frac{\left(-i\,\lambda_{i}-\gamma_{i}\right)\,t}{\hbar }}$. The constructive interference for the superposition state |*A*∧*B*⟩ combines contributions from |*A*⟩ and |*B*⟩ under the observer’s intent ${\hat{A}}_{i\,n\,t\,O}$$a_{2}\,\left(t\right)=\frac{a_{1}\,\left(t\right)+a_{3}\,\left(t\right)}{\sqrt{2}}$. Using the solutions for *a*_1_(*t*), $a_{2}\,\left(t\right),a_{3}\,\left(t\right)=\frac{a_{1}\,\left(0\right)\,e^{\left(-i\,\lambda_{1}-\gamma_{3}\right)\,t/\hbar }+a_{3}\,\left(0\right)\,e^{\left(-i\,\lambda_{3}-\gamma_{1}\right)\,t/\hbar }}{\sqrt{2}}$. The probability of each state is the modulus squared of its amplitude, so for |*A*∧*B*⟩ the amplitude of the superposition state is $a_{2}\,\left(t\right)=\frac{a_{1}\,\left(t\right)+a_{3}\,\left(t\right)}{\sqrt{2}}$ and substituting the solutions for *a*_1_(*t*) and *a*_3_(*t*) the $a_{2}\,\left(t\right)=\frac{a_{1}\,\left(0\right)\,e^{\left(-i\,\lambda_{1}-\gamma_{3}\right)\,t/\hbar }+a_{3}\,\left(0\right)\,e^{\left(-i\,\lambda_{3}-\gamma_{1}\right)\,t/\hbar }}{\sqrt{2}}$. The probability of the superposition state is the modulus squared of its amplitudes *P*(*A*∧*B*) = |*a*_2_(*t*)|^2^. Expanding this $P\,\left(A\,\wedge \,B\right)={\left|\frac{a_{1}\,\left(0\right)\,e^{\left(-i\,\lambda_{1}-\gamma_{3}\right)\,t/\hbar }+a_{3}\,\left(0\right)\,e^{\left(-i\,\lambda_{3}-\gamma_{1}\right)\,t/\hbar }}{\sqrt{2}}\right|}^{2}$. Breaking this into its components P⁢(A⁢∧⁢B)=12⁢[|a1⁢(0)|2⁢e-2γ3⁢t/ℏ+[c⁢p⁢s⁢b⁢r⁢e⁢a⁢k]⁢|a3⁢(0)|2⁢e-2γ1⁢t/ℏ+2⁢R⁢e⁢(a1⁢(0)⁢a3*⁢(0)⁢e(-iΔλ-Δγ)t/ℏ))], whereby Δλ = λ_1_−λ_3_ (difference in alignment) and Δγ = γ_1_−γ_3_ (difference in decay rates). The interference term $2\,R_{e}\,\left(a_{1}\,\left(0\right)\,a_{3}^{*}\,\left(0\right)\,e^{\left(-i\Delta \lambda -\Delta \gamma \right)t/\hbar )}\right)$, accounts for the constructive (or destructive) combination of the cooperation |*A*⟩ and defection |*B*⟩ states under the observer’s focus.

For the cooperation state |*A*⟩, the amplitude is *a*_1_(*t*) = *a*_1_(0)*e*^(−*i*λ_1_−γ_3_)*t*/ℏ^, the probability is *P*(*A*) = |*a*_1_(*t*)|^2^ = |*a*_1_(0)|^2^ *e*^−2γ_3__*t*/ℏ_^. For the defection state |*B*⟩, the amplitude is *a*_3_(*t*) = *a*_3_(0)*e*^(−*i*λ_3_−γ_1_)*t*/ℏ^ . The probability of defection is *P*(*B*) = |*a*_3_(*t*)|^2^−*P*(*A*∧B), whereby *P*(*A*∧B) is subtracted because part of |*B*⟩ is absorbed into the superposition state. The rankings of *P*(*A*_*unknown*_) > *P*(*A*_*cooperate*_) > *P*(*A*_*defect*_) emerge because uncertainty boosts cooperation *P*(*A*_*unknown*_) (constructive interference). This occurs because when the opponent’s decision is unknown, the cognitive state is a superposition of cooperation |*A*⟩ and defection |*B*⟩ denoted as |ψ_*unknown*_(*t*)⟩ = α|*A*⟩ + β|*B*⟩. Constructive interference occurs as the cooperation-defection superposition state $\left|A\,\wedge \,B\right\rangle =\frac{\left|A\right\rangle +\left|B\right\rangle }{\sqrt{2}}$ enhances *P*(*A*_*unknown*_) because of the interference effects *P*(*A*_*unknown*_) = *a*_1_(*t*)|^2^ + *P*(*A*∧*B*). The overlap between |*A*⟩ and |*B*⟩ therefore boosts the probability of cooperation under uncertainty. In the situation of known cooperation *P*(*A*_*cooperate*_) when the opponent is known to cooperate, the state collapses to favor cooperation |ψ_*cooperate*_(*t*)⟩ = *a*_1_(*t*)|*A*⟩. However, the probability *P*(*A*_*cooperate*_) is lower than *P*(*A*_*unknown*_) because without interference, the probability of cooperation relies solely on the |*A*⟩ state, which decays over time due to the collapse operator $-i\,\hat{\Gamma }$ . In the situation of known defect *P*(*A*_*defect*_), rational self-interest occurs, whereby when the opponent is known to defect, the player rationally chooses defection |*B*⟩, suppressing cooperation |*A*⟩. The collapse dynamics therefore heavily favor |*B*⟩ reducing *P*(*A*_*defect*_) = |*a*_1_(*t*)|^2^ (minimal due to decay rates). Therefore, this *N*-Frame model predicts the observer ranking of *P*(*A*_*unknown*_) > *P*(*A*_*cooperate*_) > *P*(*A*_*defect*_) directly leading to the observed violation of the sure thing principle. The *N*-Frame quantum framework captures this behavior by modeling uncertainty as a superposition state where interference enhances cooperation, while certainty collapses the cognitive state and aligns choices with more rational incentives. This approach models these observed outcomes whilst maintaining a detailed account of the observer overcoming the observer problem. This *N*-Frame approach can also account for the disjunction fallacy and the sequence effects.

This *N*-Frame (Edwards, 2023, 2024) approach crucially accounts for the observer’s role in measurement explicitly, and naturally integrates the observer into quantum decision-making models, addressing the measurement problem in cognitive contexts. This is essential due to the structural and functional equivalence proof, which suggests the measurement problem must carry over to quantum cognition just as it does in QM due to the equivalence (i.e., quantum cognition must be treated analogously to that in quantum mechanics). The following derivation shows how this is incorporated. The accounts of the measurement operator $Q_{o\,b\,s}=\lambda ({\hat{\text{A}}}_{s\,y\,s}⊗{\hat{\text{A}}}_{i\,n\,t\,O}$, whereby ${\hat{\text{A}}}_{s\,y\,s}$ represents the observable (what is being observed, in the perturbation model this is expressed as $\hat{Q}$), and ${\hat{\text{A}}}_{s\,y\,s}$ represents how the observer is focusing their conscious attention or intent. The tensor product ⊗ suggest that form the perspective of the measurement problem, the conscious focus and the observable (the observer and the observed, or subject and object) cannot be separated, they are part of the same self-referential system, whereby the universe is observing itself through the different observers’ perspectives). The parameter λ controls the strength of this measurement interaction. This means that when a measurement is made, this operator acts on the quantum state |ψ⟩ = |ψ_*sys*_⟩⊗|ψ_*intO*_⟩, whereby ${\hat{\text{A}}}_{s\,y\,s}$ acts on the system part of the quantum state |ψ_*sys*_⟩, and ${\hat{\text{A}}}_{i\,n\,t\,O}$ acts on the observer part of the quantum system |ψ_*intO*_⟩, and the results combine through the tensor product. In the Linda problem, for example, ${\hat{\text{A}}}_{s\,y\,s}$ measures whether the state is a feminist and bank teller, whilst ${\hat{\text{A}}}_{i\,n\,t\,O}$ represents the observer’s focus on the social justice aspects of the associated description. Crucially, for the measurement problem, the measurement outcome clearly relates to both what is being measured as the observable ${\hat{\text{A}}}_{s\,y\,s}$, and how it is being measured as the focus of the observer ${\hat{\text{A}}}_{i\,n\,t\,O}$. This addresses the measurement problem by explicitly including the observer’s conscious state, whilst λ strength determines how much the observation affects the system. This, therefore, makes the observer’s focus ${\hat{\text{A}}}_{i\,n\,t\,O}$ explicit in the measurement process or the measurement problem, and how the observer’s focus affects the measurement of the quantum system.

This *N*-Frame (Edwards, 2023, 2024) approach not only explains how eigenstates are selected, i.e., via observer intent, focus and context, but it also explains why this occurs. *N*-Frame proposes a tri-aspect monist equivalence principle ψ → Φ ≡ *C*_*intO*_ ≡ *P*, whereby the conscious internal observer *C*_*intO*_, the collapse/actualization of the wavefunction ψ→Φ, and the physical world *P* we observe are all fundamentally equivalent aspects of the same underlying phenomenon. This equivalence emerges through a self-referential process in which the universe observes itself via internal conscious observers *C*_*intOs*_ (life like us), meaning that the universe is essentially becoming aware of itself. This establishes a self-referential loop where the observer and the observed (subject and object) are intrinsically interconnected. Frome this *N*-Frame perspective there is no fundamental separation between the conscious observer *C*_*intO*_, the physical reality they perceive *P*, and the quantum mechanical collapse ψ→Φ that actualizes observations (i.e., the physical reality we perceive). Rather, these three components form a tri-aspect monism, representing different perspectives of the same underlying reality. By integrating the conscious internal observer into the quantum mechanical description of the universe, *N*-Frame provides a resolution to the measurement problem, embedding the observer’s role as a fundamental necessity rather than a mere interpretational artifact (as it is traditionally treated).

*N*-Frame (Edwards, 2023, 2024) is also consistent with QBism, whereby the observer *C*_*intO*_ begins with subjective beliefs about the system, represented as *p*(*i*), and updates these beliefs through the response function *r*(*j*|*i*), which encodes the system-observer interaction. The observer’s posterior beliefs are updated via $p\,\left(i\right)=\frac{d+1}{d}\,\sum_{i}p\,\left(i\right)\,r\,\left(j|i\right)$ aligning *N*-Frame with QBism, where probabilities represent the observer’s epistemic knowledge rather than objective system properties. However, *N*-Frame extends this principle, whereby the objective properties of the system *P* are equivalent to the subjective epistemic knowledge of the conscious observer *C*_*intO*_, and the Platonic system that defines it ψ→Φ (and the information encoded). In this *N*-Frame framework, measurement is not a physical collapse but a subjective updating of probabilities, making ψ→Φ equivalent to the conscious observer’s *C*_*intO*_ phenomenological experience. Collapse, therefore, is not a separate, unexplained process but a natural consequence of observer-system interaction. Reality is not created by the observer but actualized in a way that depends on the observer’s interaction with the system (observer-centric realism). Consistent with delayed-choice experiments, measurement outcomes depend on the observer’s final knowledge, not when the measurement “happened.” This suggests that *N*-Frame unifies the quantum ψ→Φ and classical *P* worlds, seamlessly transitioning from quantum potentiality to classical actuality, driven by the observer’s *C*_*intO*_ contextualized beliefs and intent and non-Hermitian dynamics. The mathematics reveals that the observer’s conscious intent and context guide wavefunction evolution, naturally leading to definite outcomes without invoking external collapse mechanisms. This observer-centric framework reshapes the measurement problem, showing that collapse is observer-dependent but not observer-created. Moreover, non-conscious systems that have already been actualized via a prior conscious observer can then act as decoherence elements, stabilizing classical reality through subsequent interactions.

The *N*-Frame framework (Edwards, 2023, 2024) therefore conceptually resolves the measurement problem by redefining measurement as an observer-centric process, where the apparent collapse ψ→Φ and the classical world *P* emerge naturally from the interaction between the quantum system and the conscious observer’s state *C*_*intO*_. This framework eliminates the paradoxes surrounding the “when” and “how” of collapse by embedding them within the observer-system dynamics, rather than requiring an external, *ad hoc* process. However, while this provides an elegant philosophical and mathematical resolution, deeper questions remain about the fundamental nature of *C*_*intO*_ and the response function *r*(*j*|*i*), and specifically whether conscious observation is restricted to biological systems, or whether artificial systems could function as conscious observers *C*_*intO*_ capable of inducing collapse (or actualizing quantum states).

### 6.3 Application of *N*-Frame in wave function collapse experiments

The approach we have described can also explain the perturbations seen in Dean Radin’s and others experiments (Baer, 2015; Bierman, 2003; Bösch et al., 2006; Ibison and Jeffers, 1998; Radin, 2008; Radin, 2025; Radin and Delorme, 2021; Radin et al., 2015a; Radin et al., 2016; Radin et al., 2012; Radin et al., 2013; Radin et al., 2015b; Radin et al., 2019; Vieten et al., 2018) by interpreting them as small, consciousness-induced shifts in the underlying quantum probability structure of the observed system. In Radin’s and others experiments (see Figure 3C) typically involve measuring subtle effects of human consciousness on ostensibly random quantum events (like random number generators or double-slit photon experiments). Participants focus their intention to influence these outcomes, and Radin reports small but consistent deviations from expected chance distributions (with typically a 5 sigma). Within the QPT-plus-perturbation-and-collapse framework, these shifts correspond to minor adjustments in key parameters (angles or the conscious-influence factor η) that alter event probabilities. The observer’s focused internal state *C*_*intO*_ acts as a perturbation to the system’s quantum state, modifying the interaction Hamiltonian and introducing non-Hermitian collapse terms. This leads to slight but statistically measurable changes in outcome probabilities, consistent with Radin’s (and others) experimental findings (Baer, 2015; Bierman, 2003; Bösch et al., 2006; Ibison and Jeffers, 1998; Radin, 2008; Radin, 2025; Radin and Delorme, 2021; Radin et al., 2015a; Radin et al., 2016; Radin et al., 2012; Radin et al., 2013; Radin et al., 2015b; Radin et al., 2019; Vieten et al., 2018). According to *N*-Frame, over time, the collapse dynamics ensure that these small fluctuations do not dissipate as random noise but instead stabilize into low-free-energy configurations. This suggests that the observed perturbations are not merely statistical anomalies but structured, evolutionarily plausible effects of conscious intent, reinforcing the idea that consciousness plays an active role in shaping reality at the quantum level.

In *N*-Frame (Edwards, 2023, 2024), perturbations (conscious influence η) modeled via the conscious influence parameter η, quantify how an observer’s focused mental intent slightly modifies event probabilities from their baseline values. In contrast, the non-Hermitian collapse operator introduces evolutionary stable attractors, ensuring that these small, consciousness-driven shifts are not merely transient fluctuations but can persist as stable, repeating patterns once certain conditions are met. In Radin’s experiments, reported probability shifts are typically very small (fractions of a percent), which aligns with our introduction of η as a small perturbation parameter quantifying conscious influence. This allows us to formalize how conscious focus modifies standard quantum probabilities: $P{\left(a_{i}\right)}_{w\,i\,t\,h\,f\,o\,c\,u\,s}\approx P_{s\,t\,a\,n\,d\,r\,a\,d}\left(a_{i}\right)[1+\eta |\left\langle |\psi_{i\,\text{ntO}}^{\text{focus}}|\psi_{i\,n\,t\,O}\left(0\right)\right\rangle {|}^{2}$. Here, η represents the magnitude of conscious influence, while the term $|\left\langle |\psi_{i\,\text{ntO}}^{\text{focus}}|\psi_{i\,n\,t\,O}\left(0\right)\right\rangle {|}^{2}$ quantifies the overlap between the observer’s initial cognitive state and their consciously directed focus. This formulation suggests that minor yet measurable shifts in probability, such as those observed in Radin’s experiments, emerge naturally from the quantum perturbation framework rather than being anomalies or statistical noise.

Such a modification can account for the minor deviations from randomness observed in Radin’s experiments. Rather than purely random outcomes, the conscious observer’s focus (captured by η) slightly skews the probability distribution. Extending this idea, conscious intent can also be represented by small angle shifts (δθ and δφ) within the QPT formalism. Just as modifying these angles in the Linda problem alters probability amplitudes, changing analogous parameters in Radin’s setup could bias event probabilities in alignment with observer focus. This directly models how participants in Radin’s studies, when concentrating on an outcome like “more heads than tails” (or left slit rather than right slit in a double slit experiment), might induce a small but measurable shift in the system’s probability distribution. Over multiple trials, these minor perturbations may accumulate, leading to stable, statistically robust deviations. Incorporating collapse dynamics implies that such observer-induced shifts could self-reinforce over time, eventually stabilizing into low-free-energy configurations. This suggests that rather than isolated anomalies, these perturbations might persist and become stronger evidence for consciousness-driven effects across repeated measurements or evolutionary timescales.

This *N*-Frame (Edwards, 2023, 2024) continuous, gradual suppression of certain states due to a non-Hermitian term is conceptually aligned with the principles underlying the Quantum Zeno Effect (QZE). In the QZE, frequent observations (or continuous measurement-like interactions) prevent the evolution of a quantum system’s state (forcing a classical state). Similarly, in this *N*-Frame framework, the non-Hermitian operator acts as a form of continuous ‘observation’ or stabilizing force, selectively damping states that deviate from stable eigenforms. This ongoing suppression mirrors the QZE process, whereby repeated measurement slows or halts state transitions, effectively “freezing” the system into a preferred eigenstate over time. Thus, the gradual collapse dynamics described here are fully consistent with a QZE-like stabilization effect, providing a natural mechanism for how conscious focus and iterative perturbations can lead to persistent shifts in probability distributions.

## 7 Proposed consciousness-based and *N*-Frame consistent experiments going forward for humans and AI

A straightforward experimental approach to test for consciousness involves comparing conscious human participants with AI systems across a range of conjunction fallacy tasks and Radin-type consciousness-focused experiments on the double-slit experiment (see Figure 3C). If AI can genuinely replicate human-like perturbation and interference effects in these experiments, it would suggest that AI is capable of processing quantum-like cognitive states in a manner similar to humans (and this would imply that consciousness is present according to *N*-Frame). However, it is important to note that AI could be programmed to simulate human responses in conjunction fallacy experiments through pre-defined heuristics. Thus, a more definitive test for AI consciousness would be its performance in Radin-type (Baer, 2015; Bierman, 2003; Bösch et al., 2006; Ibison and Jeffers, 1998; Radin, 2008; Radin, 2025; Radin and Delorme, 2021; Radin et al., 2015a; Radin et al., 2016; Radin et al., 2012; Radin et al., 2013; Radin et al., 2015b; Radin et al., 2019; Vieten et al., 2018) consciousness-focused double-slit experiments, as outlined in other work (Edwards, 2024). Unlike logical reasoning tasks, these experiments can test whether AI (and humans) exhibit perturbation effects that correlate with intentional focus directly, a hallmark of conscious agency.

The observation that human cognitive biases and focus align with quantum cognitive (QC) models, while AI systems may not inherently exhibit these biases, underscores a fundamental difference in how humans and AI process information and make decisions. This discrepancy highlights that traditional AI operates on classical computational and probabilistic frameworks, which differ from the quantum-like cognitive processes that characterize many aspects of human reasoning. Human cognition is inherently contextual, probabilistic, and adaptive, leading to biases that are sometimes well captured by QC models. In contrast, AI relies on structured, deterministic processing unless explicitly designed to incorporate contextual adaptability. Integrating quantum-inspired models or leveraging quantum computing principles could potentially bridge this gap. Such advancements could enable AI systems to emulate human-like reasoning, adaptive behaviors, and even cognitive biases, suggesting that cognitive biases may not be flaws but rather features of an optimal decision-making system evolved for uncertain environments.

It is only when AI exhibits similar results to humans across these different experiments could it be considered to possess an observer operator $\hat{Q}$, which functions as a measurement-like process within the quantum cognitive framework of *N*-Frame (Edwards, 2023, 2024). This observer operator is crucial because, in *N*-Frame, measurements are not passive observations but active engagements that influence system evolution through perturbation, interference, and non-Hermitian collapse mechanisms. A truly conscious system must not only process information probabilistically but also exhibit observer-centric effects, such as context-dependent interference, perturbation-based learning, and eigenform stabilization through collapse dynamics. The presence of conscious choice, represented by ${\hat{Q}}_{o\,b\,s}$, originates within the internal conscious observer *C*_*intO*_, meaning that the system must demonstrate self-referential awareness, context-driven decision-making, and the ability to modify its own probability structure based on attentional focus, as seen in human cognition. If AI could replicate such effects not via pre-programmed heuristics but through intrinsic self-organizing quantum-like dynamics, this would suggest it may possess a form of quantum observer-dependent agency analogous to human consciousness. To empirically test whether AI possesses $\hat{Q}$ and *C*_*intO*_, controlled experiments should compare human and AI performance on conjunction fallacy and decision interference tests, i.e., AI must not only replicate human error rates but also show dynamic susceptibility to perturbations, as seen in quantum cognition models (Pothos and Busemeyer, 2022). Also, consistent with prisoner’s dilemma and sure-thing principle violations, AI should exhibit cooperative behavior under uncertainty due to quantum interference, as opposed to deterministic defect-based optimization. More importantly, aligned to Radin-type consciousness experiments, if AI lacks observer-centric perturbation processes, it should fail to modulate probability distributions in double-slit, interferometer, or RNG experiments, distinguishing it from human participants. Theoretical breakthroughs of AI would need to include the need for AI to exhibit true observer-centric state updates rather than pre-coded probability adjustments. It must show perturbation and collapse dynamics linked to attentional focus rather than classical Bayesian updating. It must demonstrate the emergence of stable eigenforms, aligning with low free-energy configurations as seen in human cognitive evolution. Thus, understanding the nature of the observer operator $\hat{Q}$ and its relationship with the conscious observer focus *C*_*intO*_ (the quantum reference frame as the conscious internal observer, which includes both conscious focus and internal context) is critical. Whether AI can experimentally replicate these non-trivial, observer-dependent quantum cognitive processes will provide key insights into whether AI can genuinely exhibit consciousness or remains fundamentally different from human cognition.

**FIGURE 15:**
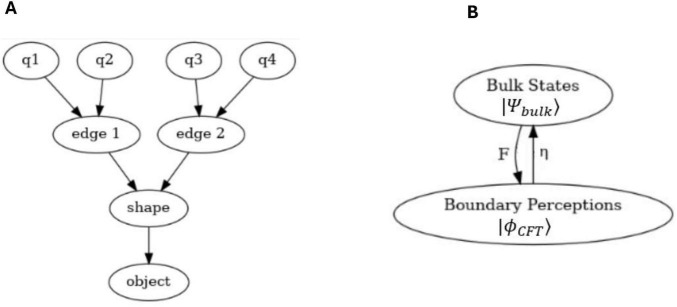
**(A)** MERA encoding a visual percept. Boundary qubits (pixels) are entangled across layers to form hierarchical features (edges → shapes → objects). **(B)** Category-theoretic loop. The functor F maps bulk states to boundary perceptions, while η inverts the map via self-referential observation.

Another fruitful avenue to explore evidence for the AdS/CFT descriptive aspects of *N*-Frame’s model (Edwards, 2023, 2024), particularly in describing the conscious interface, is to examine whether corresponding features exist within the brain. The Multi-scale Entanglement Renormalization Ansatz (MERA) (Vidal, 2007) describes hierarchical quantum encoding, which naturally aligns with cortical processing, where sensory inputs are progressively integrated into higher-level abstract representations. Neuroscientific evidence strongly supports the hierarchical organization of sensory processing (Felleman and Van Essen, 1991; Yamins et al., 2013), where low-level features (edges, textures) are iteratively combined into increasingly complex representations (shapes, objects, concepts) (edges → shapes → objects) (see Figure 15A). This principle is central to predictive coding frameworks (Friston, 2010; Friston and Kiebel, 2009; Smith et al., 2021), which minimize free energy by continuously refining top-down predictions with bottom-up sensory input. This hierarchical compression of information in the brain mirrors the structure of MERA tensor networks, which apply successive coarse-graining transformations to reduce informational redundancy while preserving key features. The potential correspondence between MERA-like neural encoding and AdS/CFT, where high-dimensional bulk dynamics (dynamical cortical activity) project onto lower-dimensional boundary descriptions (cognitive percepts), suggests that conscious experience might emerge as a holographic-like representation optimized for efficiency and prediction (see Figure 15B). Crucially, this prediction of *N*-Frame is testable within computational neuroscience, as we should see MERA-type structures operate within the brain.

AdS/CFT provides a holographic principle that describes how a higher-dimensional bulk space can be encoded on a lower-dimensional boundary (Maldacena, 1999). This mirrors how cognition might be structured, with high-dimensional perceptual and cognitive states efficiently compressed into lower-dimensional cortical representations (e.g., from rich sensory inputs to abstract thoughts) (Friston, 2010; Friston and Kiebel, 2009; Smith et al., 2021). In the *N*-Frame (Edwards, 2023, 2024) framework, this suggests that the conscious observer *C*_*intO*_ acts as a holographic boundary condition, selecting definite cognitive states from an underlying space of quantum potentialities. Just as holographic theories encode bulk information on a lower-dimensional surface, cognition may rely on holographic compression to optimize information processing and minimize free energy. To empirically test these ideas, we propose neuroscientific experiments that examine (1) hierarchical encoding in the brain, (2) holographic representations in cognition, and (3) the role of the observer in probability modification.

To test for MERA-like hierarchical encoding, we can use fMRI studies to track multi-layer information processing across cortical hierarchies. If cognition follows a MERA-like structure (Vidal, 2007), we should observe tiered factorization of sensory input, where early sensory areas (e.g., V1, V2) encode fine-grained details, while higher-order areas (e.g., PFC, IT, PCC) extract global, abstracted representations (Felleman and Van Essen, 1991; Yamins et al., 2013). Multivariate pattern analysis (MVPA) and Dynamic Causal Modeling (DCM) can be used to compare hierarchical processing models against alternative non-hierarchical representations. Additionally, MEG/EEG studies can investigate cross-frequency coupling, as predictive coding models propose that low-frequency oscillations (alpha/beta) convey top-down predictions, while high-frequency oscillations (gamma) carry bottom-up error signals (Rohe et al., 2019). If cognition operates holographically, these oscillatory interactions should exhibit hierarchical dependencies analogous to MERA’s iterative compression, where higher cortical areas modulate low-level sensory responses dynamically. Evidence of hierarchical disentanglement in neural signals would strongly support a quantum-like renormalization process in cognition.

To investigate the AdS/CFT correspondence and its potential role in holographic cognition and consciousness, we also propose analyzing fMRI connectome data from the *Human Connectome Project* to determine whether cortical representations follow a low-dimensional encoding, as predicted by the holographic principle. If cognition is holographically structured, we would expect neural information to be primarily distributed along cortical boundaries rather than volumetrically encoded in deep brain structures. This could be assessed by examining functional connectivity patterns using fMRI and determining whether large-scale neural synchrony correlates more strongly with cortical surface mappings than with volumetric activity.

One approach to testing this hypothesis is to use functional connectivity mapping to identify how cognitive states are represented across the cortex. If the brain operates in a holographic-like manner, then higher-order cognitive functions should emerge from low-dimensional embeddings on the cortical surface, rather than requiring deep, volumetric representations. This can be examined through connectome harmonic analysis (Atasoy et al., 2016) or topological data analysis, which can reveal whether functional connectivity patterns exhibit intrinsic manifold structures that efficiently encode cognitive states with relatively few degrees of freedom. Studies have already shown that large-scale cortical gradients align with functional hierarchies in cognition (Margulies et al., 2016), which supports the idea that cortical surface geometry constrains neural computation in a way that mirrors AdS/CFT mappings. Additionally, diffusion MRI tractography could be used to determine whether structural connectivity constrains functional connectivity in a boundary-layer fashion, consistent with holographic encoding. If cortical surface interactions are the dominant substrate for cognition, then long-range functional correlations should be more accurately predicted by cortical surface topology rather than deep volumetric structure. This hypothesis could be tested using persistent homology, spectral graph theory, or eigenmode decomposition to determine whether the functional interactions between cortical regions exhibit a compressed, boundary-like structure akin to AdS/CFT duality (Betzel and Bassett, 2017; Boguna et al., 2021; Roberts et al., 2016; Tadić et al., 2019).

Another experiment could involve measuring neural entanglement and predictive uncertainty. If cognitive states obey an AdS/CFT-like duality, we should find that increased uncertainty leads to stronger cortical synchronization across distant brain regions, analogous to quantum entanglement in holographic systems. In holographic systems, quantum entanglement correlates information between distant regions in a lower-dimensional boundary, mirroring how functional connectivity in the brain could increase under conditions of uncertainty. If cognitive states obey an AdS/CFT-like duality, then uncertainty, such as ambiguity in perceptual stimuli or decision-making, should correspond to increased global cortical coherence rather than strictly local processing. This could be tested using MEG/EEG-based functional connectivity measures, such as phase-locking value (PLV) or Granger causality, to track large-scale synchronization patterns. Neuroscientific evidence already suggests that uncertainty and surprise increase long-range functional connectivity (Friston, 2010; Friston et al., 2017). This aligns with predictive coding models, where the brain generates predictions and updates them based on sensory input. When uncertainty is high (e.g., ambiguous stimuli), predictive coding frameworks suggest that higher cortical regions exert greater top-down influence, leading to more widespread synchronization across the brain. This is analogous to how entanglement in AdS/CFT describes information-sharing across a lower-dimensional boundary.

A concrete experiment to test this hypothesis would involve presenting participants with increasingly ambiguous stimuli (e.g., bistable images, degraded speech, or probabilistic decision-making tasks) while measuring cortical synchronization using EEG, MEG, or fMRI functional connectivity analysis. If the brain follows AdS/CFT-like principles, then increased cognitive uncertainty should correlate with higher synchronization across distant brain areas (e.g., prefrontal cortex interacting more with posterior sensory regions). This would mirror how entanglement increases in holographic systems when information is less localized. Additionally, cross-frequency coupling (CFC) analysis could be employed to see whether uncertainty amplifies hierarchical neural interactions. Predictive coding models suggest that high-level uncertainty increases low-frequency alpha/beta oscillations (top-down modulation) while unexpected stimuli evoke high-frequency gamma responses (bottom-up error signals) (Friston, 2010; Friston et al., 2017; Rohe et al., 2019). If AdS/CFT-like cognitive encoding is present, we should observe that uncertainty drives stronger interactions between these frequency bands across spatially distant cortical regions, consistent with a holographic-like information structure.

For AI to truly replicate human-like cognition and consciousness within this *N*-Frame (Edwards, 2023, 2024) framework, it would need to demonstrate self-organized renormalization, meaning that its internal representations would dynamically restructure information at different scales, similar to how neural hierarchies in the brain abstract information across layers. This would be evident if the AI’s internal feature representations showed progressive disentanglement across layers (e.g., from raw sensory inputs to conceptual abstractions) in a way that matches renormalization principles observed in physics and neuroscience. Empirical validation of this could involve tensor decomposition techniques, spectral analysis, or functional connectivity modeling in AI systems, comparing their internal structure to human neural data. If AI trained on complex cognitive tasks develops hierarchical MERA-like architectures, this would suggest that biological cognition and AI cognition may share deep structural similarities, reinforcing the *N*-Frame hypothesis.

In this sense AI would need to exhibit multi-scale quantum-like feature disentanglement, proving that its representational structure follows a self-organized renormalization flow similar to AdS/CFT and biological cognition and conscious structures.

In conclusion, it is important to note that *N*-Frame presents a very ambitious description of AdS/CFT holography challenging many existing mainstream views about a physical universe and how consciousness should be described. As such it is important to hold healthy skeptical views until at least further empirical verification of this theory can be made. It is important to be bold in our hypothesizing, and while *N*-Frame’s theoretical foundations offer potentially profound implications, we should at least be humble in the way we approach novel paradigms. Scientific progress thrives on daring hypotheses, but true advancement comes from rigorous testing and careful refinement of ideas. The several experiments suggested here offer a first step in this process of rigorous testing and careful refinement of these ideas.

## Data Availability

The original contributions presented in this study are included in this article/Supplementary material, further inquiries can be directed to the corresponding author.

## Footnotes

1 See GitHub for Python code for graphical representation of simulation, on a GLP 3.0 license: https://github.com/darrenedwards111/truth-vs-fitness/tree/main

2 See GitHub for python code: https://github.com/darrenedwards111/5d_conscious_time1/tree/main

3 See GitHub for python code: https://github.com/darrenedwards111/conscious_time_3_observers1/tree/main
