# Supplementary material for: Further N-Frame networking dynamics of conscious observer-self agents via a functional contextual interface: predictive coding, double-slit quantum mechanical experiment, and decision-making fallacy modeling as applied to the measurement problem in humans and AI
Source: Front Comput Neurosci. 2025 Apr 1;19:1551960. doi: 10.3389/fncom.2025.1551960 (PMC11996842; doi:10.3389/fncom.2025.1551960)
Supplement: Supplementary file 1 [file Data_Sheet_1.pdf]

Supplementary Material: Further *N*-Frame dynamics

Supplementary 1 – The revised hypercomputational Church-Turing thesis.

Conjecture: While most physical processes are computable and can be simulated by a Turing machine, spacetime itself may encode hypercomputational structures. Some aspects of physical reality, particularly those related to quantum gravity, black hole interiors, and the observer's C_{intO} role, involve processes that are fundamentally noncomputable and cannot be fully captured by any algorithmic model, including quantum computers.

The key components of this revision include: (1) Physics is not strictly computable. The evolution of spacetime geometry near singularities, in quantum gravity, or in relation to observers may involve non-Turing-computable functions. This means that even perfect knowledge of initial conditions does not guarantee that the system's future state can be computed. (2) Quantum mechanics is computable, but quantum gravity may not be. Quantum field theories (QFTs) and quantum mechanics obey the standard Church-Turing thesis since quantum computers can simulate their dynamics. However, quantum gravity may encode noncomputable interactions, particularly in cases where spacetime emerges from more fundamental non-Turing-computable physics. (3) Hypercomputational processes exist in nature. Certain spacetime solutions, such as black hole interiors, require infinite computational resources to predict their full evolution. The observer's interaction with spacetime (in the *N*-Frame model) introduces hypercomputational effects beyond Turing computation that may explain this. (4) AI cannot fully simulate consciousness unless it interacts with hypercomputational physics. If consciousness depends on noncomputable interactions with spacetime, then no AI based purely on Turing computation can be conscious. This places a fundamental limit on AI and machine learning as models of human cognition.

The implications of the revised hypercomputational Church-Turing-Edwards thesis suggests that: (1) Not all of physics can be simulated on a Computer. If spacetime encodes noncomputable structures, then no computer (classical or quantum) can simulate all aspects of reality. This fundamentally limits computational physics, meaning that certain predictions in quantum gravity may be unknowable. (2) The observer C_{intO} plays a fundamental role in reality. If an observer's boundary action influences bulk spacetime in a hypercomputational way, then the act of measurement itself may be noncomputable. This suggests that physics is not fully independent of the observer, challenging the idea of an objective, algorithmically predictable universe. (3) Quantum gravity theories must incorporate noncomputability. Current quantum gravity theories (like loop quantum gravity and string theory) assume that physics is computable. However, if spacetime has hypercomputational aspects, then a new framework such as *N*-Frame is required, potentially involving nonalgorithmic path integrals, observer-dependent gravitational states, and geometric structures beyond Turing computation.

Mathematical formulation of the revision. In standard computability theory a function $f(x)$ is Turing-computable if there exists an algorithm that computes $f(x)$ for all x. A physical theory is computable if all observables $O(x)$ can be computed by a finite algorithm. In the revised thesis, we introduce hypercomputable physics: $O(x) = f(x) + H(x)$, whereby $f(x)$ is the Turing-computable part of physics; $H(x)$ is a hypercomputational term that cannot be generated by any finite algorithm. If $H(x) \neq 0$ in spacetime physics, then no Turing machine can fully predict physical observables, the evolution of spacetime is not fully computable, and the laws of physics may be incomplete from a computational perspective.

Experimental tests for the revised thesis to validate the hypercomputational nature of spacetime, we must identify physical effects that defy computability: Black hole information retrieval beyond Turing computation. If information is preserved in a noncomputable form inside black holes, then no algorithm can reconstruct the full information state. Hawking

radiation may encode hypercomputable correlations, making black hole evaporation unpredictable from a standard computational perspective. Quantum measurement and moncomputability. If quantum measurement involves an observer's interaction with hypercomputational spacetime, then certain wavefunction collapse events may be fundamentally uncomputable. This would imply free will cannot be reduced to algorithms. AI consciousness and hypercomputational constraints. If AI is limited to Turing computation, but consciousness requires hypercomputational physics, then AI can never truly achieve conscious self-awareness. This could be tested by identifying cognitive processes that exceed known computational models.

Supplementary 2 – The evolution of the universe, a teleological universe.

It is difficult to say where did the fine-tuned parameters come from. Some argue for some all-powerful theological God, such as described in the Christin faith (Meyer, 1999, 2020). However, perhaps a more empirically valid assumption is to assume that we live in a multiverse as suggested by many physicists (perhaps linked to 5D conscious time), and that there is some evolutionary selection at this muti-verse level. Others, such as Campbell have also used the idea of Universal Darwinism (Campbell, 2011) to explore how the application of Darwinian theory of evolution can extend beyond biology to fields such as cosmology and quantum physics, proposing a universal framework of Darwinism. This seems broadly consistent with cosmological natural selection by Smolin (Smolin, 1992, 2004). Cambell, however, suggests that the process of natural selection and adaptation fundamental to biological evolution, might also explain phenomena across a wide range of scientific domains, highlighting the role of knowledge and in reducing entropy.

So, cosmological evolution seems to be selecting for emergent complexity within our universe, but from simple fundamental laws. An example of this is the "eightfold way" that

describes symmetry group of fundamental subatomic particles known as hadrons by discovering the Lie algebra for the special unitary group 3 (Gell-Mann, 1961; Gell-Mann & Yuval, 1964) that led to the quark model of standard fundamental physics (da Veiga & O'Carroll, 2007; Sener & Schulten, 2015). These quark dynamics are crucial for the stability and characteristics of the protons and neutrons that make up atomic nuclei. Without stable protons and neutrons and the forces that hold them together in nuclei, atoms as we know them could not exist, and without atoms complex life could not exists, so these simple rules play an important role in describing the emergence of complexity, as they have a crucial role for atomic stability.

Simplicity can be found in physics (specifically thermodynamics and chemistry) such as the Ideal Gas law by $\acute{E}$mile Clapeyron in 1834 and derived from even simpler laws by Boyle's law, Charles's, Avogadro's principle, and Gay-Lussac's law (Arnaud et al., 2011; Laugier & Garai, 2007) which is expressed as $PV = nRT$, whereby P is the pressure of the gas, V is the volume of the gas, n is the amount of substance, R is the ideal gas constant, and T is the temperature. This provides a good approximation of the behavior of many gases under most conditions, as it describes the molecules as in constant, random motion. As the gas system's temperature increases, the kinetic energy of the gas molecules increases, leading to more random motion and, consequently, an increase in entropy. This is an example of simple rules explaining highly random entopic phenomena.

Entropy and complexity are related concepts, but these are not opposite concepts, rather the concept of entropy is on the opposite continuum to order, and complexity is on the opposite continuum to simplicity. The relationship between entropy and complexity varies depending on context, in physics such as thermodynamics, entropy is the measure or disorder or randomness. An increase in randomness is an increase in entropy. Complexity is the degree to which a system is structured, but in some cases as complexity increases so does entropy.

For example, Gell-Mann (Gell-Mann, 1997) also referred to the complexity that emerges from these simple beginnings, such as the complex network or relationships linking the human race to itself and the rest of the biosphere, where all aspects affect each other within this complex network. This complex network leads to highly entropy behavior in chaos theory, such as the butterfly effect. In other situations, high entropy does not mean high complexity, such as in the example of highly disordered gas states that have high entropy and low complexity.

Entropy describes the degree of uncertainty in a system. At the micro level, humans as organic self-organizing systems, resist entropy (in this case metabolic entropy) as part of their intrinsic process for survival and development (Aoki, 1991). This resistance to entropy is fundamentally tied to the principles of thermodynamics, which describe the behavior of energy and matter. In thermodynamics, entropy is a measure of disorder or randomness in a system. For a living organism, an increase in entropy would correspond to a breakdown in the order that is necessary for life processes. However, living organisms maintain their internal order and complexity by constantly exchanging energy and matter with their environment. This process is fundamentally tied to metabolism, the set of life-sustaining chemical reactions that enables organisms to grow, reproduce, maintain their structures, and respond to their environments. Metabolism involves both the buildup of complex molecules from simpler ones (anabolism) and the breakdown of complex molecules into simpler ones (catabolism), releasing energy. This energy is used to maintain the organism's low-entropy state, effectively resisting the natural tendency towards disorder. Through metabolic processes, organisms like humans import free energy (primarily in the form of food) and export entropy in the form of waste and heat to maintain their highly ordered state. Furthermore, self-organization is a key aspect of living systems, enabling them to develop complex structures and functions from relatively simple components without being directed by an external source. This self-

organization is evident in processes ranging from the formation of cellular structures to the development of complex organisms and ecosystems. It is driven by the laws of physics and chemistry, operating under the constraints of biological evolution and the organism's interaction with its environment.

Through the principles of cosmological natural selection, the multiverse evolves towards a state characterized by universes optimized for complexity. So, the second question is why should the principles of cosmological natural selection necessarily allow for universes optimized for complexity that embody a form of teleological evolution, where meaning in the universe is evolutionarily selected? For this, a deeper dive into complex life forms such as human evolution is required. If cosmic evolution or universal Darwinism selects complexity that form conscious organisms with conscious states, and these conscious organisms form purposeful values, then it becomes clear that we could describe the universe as a teleological universe, especially when we consider the self-referential nature of the universe as *N*-Frame describes, where the conscious observer is an active participant in the reality is creates self-referentially.

Supplementary 3 – Classical dynamics of the gravitational field in the bulk AdS space are derived from the holographic principle of the AdS/CFT correspondence.

The Einstein equations being referred to in this context are the classical gravitational field equations derived from the bulk action in the AdS/CFT correspondence. Specifically, when the full boundary action (including observer-specific terms) is extremized, it leads to equations of motion that, in the appropriate limit (large N or large spin), correspond to the Einstein equations in the bulk AdS space.

This framework is particularly important in the *N*-Frame model, where the observer's influence is explicitly encoded in the boundary dynamics. The observer's boundary action

S_{obs} extends beyond standard AdS/CFT boundary conditions by incorporating both Turing-computable and hypercomputational capabilities. This enrichment of the boundary action modifies the extremization process and leads to an emergent bulk geometry that reflects not only conventional holographic physics but also the cognitive constraints and computational properties of the observer.

In the AdS/CFT duality, the bulk theory (a gravitational theory in AdS space) is holographically dual to a conformal field theory (CFT) living on the boundary. This means that every physical process occurring in the bulk $d + 1$-dimensional spacetime can be encoded in terms of a d -dimensional CFT on the boundary. The standard bulk action is given by the Einstein-Hilbert action: $S_{\text{bulk}} = \frac{1}{2k}\int_M d^{d+1}x\sqrt{-g_{\mu\nu}}(R - 2\Lambda + L_{matter})$, whereby $g_{\mu\nu}$ is the bulk metric; R is the Ricci scalar; Λ is the cosmological constant, which is negative for AdS spaces; L_{matter} represents any additional bulk matter contribution; and $k = 8\pi G_N$ is the gravitational coupling constant.

To properly define the variational principle, we must also include boundary terms in the action. In the standard AdS/CFT setup, the total action is: $S_{\text{total}} = S_{\text{bulk}} + S_{\text{boundary}}$ where the boundary action includes: (1) The Gibbons-Hawking-York (GHY) term: $S_{\text{GHY}} = \frac{1}{k}\int_{\partial M} d^d x\sqrt{-\gamma}K$, ensuring a well-posed variational principle. (2) The observer-specific contribution S_{obs}, unique to the *N*-Frame model: $S_{obs} = \int_{\partial \text{M}_{obs}} d^d x\sqrt{-\gamma}(L_{\text{eff}}(\phi_{\text{obs}}) + L_H\big(H(\phi_{\text{obs}})\big))$, whereby γ_{ab} is the induced metric on the observer's boundary ∂M_{obs}; $L_{\text{eff}}(\phi_{\text{obs}})$ is the Turing-computable part of the observer's effective Lagrangian density; $L_H\big(H(\phi_{\text{obs}})\big)$ represents the hypercomputational contribution, extending beyond standard algorithmic computability; and $H(\phi_{\text{obs}})$ introduces non-Turing-computable observer effects into the theory. This observer-specific boundary action is fundamentally different from

standard boundary terms in AdS/CFT because it explicitly depends on observer degrees of freedom, influencing how bulk fields evolve.

The Einstein field equations arise from extremizing the total action. The variation of the bulk action gives: $\delta S_{\text{bulk}} = \frac{1}{2k}\int_M d^{d+1}x\sqrt{-g_{\mu\nu}}(Rg_{\mu\nu} - \Lambda g_{\mu\nu} - 8\pi G_N T_{\mu\nu})\delta g^{\mu\nu}$. For consistency, we must ensure that the boundary variations vanish, which requires: $\delta S_{\text{boundary}} = 0$. In the standard AdS/CFT setting, Dirichlet boundary conditions $\delta g_{ab} = 0$ are imposed to fix the metric at the boundary, ensuring consistency with the dual CFT stress tensor. However, in the *N*-Frame model, we now have an additional observer-dependent boundary contribution, meaning the extremization condition is modified as: $K_{ab} - K\gamma_{ab} = k(T_{ab}^{\text{CFT}} + T_{ab}^{\text{obs}})$, whereby K_{ab} is the extrinsic curvature of the boundary; K is its trace; T_{ab}^{CFT} is the holographic stress-energy tensor of the boundary CFT; and T_{ab}^{obs} is the observer-specific stress-energy tensor arising from S_{obs}. Since the bulk metric must satisfy the Einstein field equations, the modified form is: $R_{\mu\nu} - \frac{1}{2}Rg_{\mu\nu} + \Lambda g_{\mu\nu} = 8\pi G_N(T_{\mu\nu}^{\text{matter}} + T_{\mu\nu}^{\text{obs}})$.

The observer's influence on Bulk gravity can be shown, whereby the presence of $T_{\mu\nu}^{\text{obs}}$ means that the bulk metric is influenced by observer-specific effects, potentially modifying how spacetime curvature responds to stress-energy. If hypercomputational contributions exist i.e., $L_H\big(H(\phi_{\text{obs}})\big) \neq 0$, then the bulk equations of motion become nonlocal in an observer-dependent way. The geometry itself encodes non-Turing-computable information, and the classical Einstein equations emerge only at a semiclassical limit where hypercomputational contributions become negligible.

The implications of observer-specific contributions include: (1) The standard AdS/CFT duality is modified by allowing the observer's cognitive constraints to alter boundary conditions, leading to bulk metric modifications. (2) Instead of static Dirichlet conditions, the observer's action introduces a dynamically evolving boundary condition,

making the bulk solution history-dependent. (3) If S_{obs} includes hypercomputational terms, this suggests a connection between quantum gravity and computational complexity theory, as well as the nature of spacetime emergence and information processing. The extremization of the total action, including the observer's contribution, modifies the Einstein field equations by introducing a new observer-dependent stress-energy term. This means that in the N-Frame model, spacetime curvature is influenced not just by traditional holographic stress-energy but also by observer-based computational properties.

Supplementary 4 – How the hypercomputational contribution integrates into the variational principle to yield a modified Lagrangian formulation.

The connection is established through the variational principle. In conventional physics, a Lagrangian is a function (or density) that, when integrated over spacetime to form an action S and then extremized (i.e., when $\delta S = 0$), yields the equations of motion (such as Einstein's equations). In our framework, the observer's boundary action S_{obs} contributes directly to the total action. Let's break this down mathematically: (1) Total Action: We write the total action as $S_{\text{total}} = S_{\text{bulk}} + S_{\text{boundary}}$, whereby S_{bulk} is the standard gravitational (or matter) action and $S_{\text{boundary}} = S_{\text{GHY}} + S_{\text{obs}}$. (2) Observer's Boundary Action: The observer's contribution is given by $S_{obs} = \int_{\partial \mathrm{M}_{obs}} d^d x \sqrt{-\gamma}(L_{\text{eff}}(\phi_{\text{obs}}) + L_H(H(\phi_{\text{obs}})))$, whereby $L_{\text{eff}}(\phi_{\text{obs}})$ is the standard (Turing-computable) part of the observer's Lagrangian density, $L_H(H(\phi_{\text{obs}}))$ is the additional hypercomputational contribution, $\sqrt{-\gamma}\, d^d x$ is the invariant measure on the observer's boundary $\partial \mathrm{M}_{obs}$. (3) Mapping to Emergent Geometry: The bulk geometry is determined by a mapping of the form $g = F(b, H(b))$, whereby b represents the Turing-computable boundary data (derived from ϕ_{obs}) and $H(b)$ represents the hypercomputational augmentation. The presence of $H(b)$ means that the effective boundary information is

enriched beyond what is algorithmically predictable. (4) Derivation via Extremization: The total action S_{total} is then used in a path-integral or variational formulation: $Z = \int \mathrm{D}[g] \exp\left(\frac{i}{\hbar} S_{\text{total}}[g, \phi_{\text{obs}}, H(\phi_{\text{obs}})]\right)$. In the semiclassical (large-limit) regime, the path integral is dominated by configurations $g = g_{\text{cl}}$ that extremize S_{total}, i.e., $\delta S_{\text{total}} = 0$.

The condition $\delta S_{\text{total}} = 0$ produces Euler–Lagrange equations, whereby these are the classical equations of motion (for example, Einstein's equations in the gravitational context). Since S_{total} includes both the standard term $L_{\text{eff}}(\phi_{\text{obs}})$ and the hypercomputational term $L_H\big(H(\phi_{\text{obs}})\big)$, the resulting equations of motion (and hence the emergent Lagrangian laws) naturally incorporate both computable and hypercomputable contributions.

So, why does this lead to a Lagrangian? The entire procedure of obtaining the dynamics of the system is based on the variational principle. By constructing an action S_{total} that includes these enriched boundary contributions, and then extremizing that action, we derive a Lagrangian formulation of the system's dynamics. The additional hypercomputational terms modify the effective Lagrangian, leading to classical equations that reflect the observer's enhanced processing capabilities. In essence, the hypercomputational terms ensure that the emergent bulk geometry is not solely the product of algorithmic, Turing-computable processes; they enrich the boundary data such that the variational procedure yields equations of motion (i.e., a Lagrangian formulation) that encapsulate both standard and non-algorithmic aspects. This enriched Lagrangian is what governs the dynamics of the universe in our model.

Supplementary 5 – Conscious time.

It is also possible to one could embed this extra "conscious dimension" into a unified field-theoretic framework, reminiscent of how Einstein unified "metric" and "field" (in his general

relativity theory) by treating the spacetime metric itself as the gravitational field (Einstein, 1916) (see Supplementary 2 for further details).

Let M_5 be a 5-dimensional manifold with coordinates x^{μ}, τ whereby $\mu = 0,1,2,3,$ and extra dimensions τ is an extra dimension that can be identified as "conscious." The 4D subspace (spanned by x^{μ}) is interpreted as ordinary spacetime, while τ extends beyond standard physics. For a unified metric and conscious dimension we can propose a single 5D metric $G_{AB}(A, B - 0,1,2,3,4)$ that includes τ. This can be denoted generically as $ds^2 = g_{\mu,\nu}(x^{\alpha}, \tau)dx^{\mu}dx^{\nu} + X^2(x^{\alpha}, \tau)d\tau^2$, whereby (1) $g_{\mu,\nu}$ depends on both x^{α} (the usual 4D) and possibly on τ. (2) $X(x^{\alpha}, \tau)$ is a scalar function ("warp factor" or coupling) controlling how τ enters the geometry. This single 5D metric is intended to unify (in analogy to Einstein's approach for 4D gravity) both the notion of "spacetime" and the extra "conscious dimension." In principle, the entire geometry G_{AB} is "the field."

For the 5D action, consider a 5D Einstein-Hilbert action, plus possible matter and consciousness terms: $S_{5D} = \int d^5 x\sqrt{-G}[R_5 + L_{mat}\ (G_{AB}, \Phi_i, \dots)\ + \alpha L_C(G_{AB}, C, \dots)]$, whereby R_5 is the Ricci scalar computed from G_{AB}. L_{mat} is a standard matter Lagrangian for fields Φ_i (e.g., electromagnetic or scalar fields) living in 5D. L_C is a new "consciousness" Lagrangian or some set of terms that attempt to encode "computational" or "observer-interface" effects. A coupling constant α sets the strength of "conscious" terms relative to gravity and matter. Varying this action with respect to G_{AB} yields a set of 5D field equations:

$\delta_{G_{AB}} S_{5D} = 0 \Rightarrow R_{AB} - \frac{1}{2}R_5 G_{AB} = k(T_{AB}^{matter} + T_{AB}^{consciousness}$, whereby R_{AB} is the 5D Ricci tensor (built from the 5D metric G_{AB}). $R_5 \equiv G^{AB}R_{AB}$ is the 5D Ricci scalar. T_{AB} is the 5D stress-energy (or energy–momentum) tensor associated with matter and consciousness fields, whereby T_{AB}^{matter} is the usual stress-energy for standard fields (electromagnetism, scalar fields, etc.), and $T_{AB}^{consciousness}$ is a hypothetical term captures whatever "energy–

momentum" attribute that can be applied to a consciousness (or observer-centric) field. k is a coupling constant (in 4D one often has $k = \frac{8\pi G}{c^4}$, in 5D it may differ).

To apply an "energy–momentum" to a "consciousness field," we would need to decide what type of field represents consciousness such as scalar field Φ, vector field C^μ or more exotic fields (spinor, tensor, etc.). In pure geometry form one might try to have no separate "conscious field" but rely on the 5D metric alone. This is akin to Kaluza–Klein or early unified theories where electromagnetism emerges from an extra dimension. Here, we require an ansatz (special form) for G_{AB} that, upon dimensional reduction from 5D to 4D, yields something interpretable as "conscious states" or "observer boundary." When applying an extra conscious field, a scalar field example could include a conscious field $C(x^\mu, \tau)$ or $\tau(x^\mu)$ inside L_C which yields extra field equations, whereby the full system is then

$$C\begin{cases} R_5^{AB} - \frac{1}{2} R_5 G_{AB} = T_{mat}^{AB} + T_C^{AB}, \\ \Box_5 - \frac{\partial V}{\partial C} = 0, \qquad etc. \end{cases}$$

with $\Box_5$ the 5D d'Alembert operator in the geometry G_{AB}.

"Consciousness" then shows up as an additional source of curvature or stress-energy.

To link this to the idea of a computational boundary or "Markov blanket": 4D Hypersurfaces in M_5 (e.g., $\tau = const$) might act as "slices" where the observer's internal states reside. The boundary's geometry or measure could limit how much information can be "processed" or "encoded," analogous to how horizon area limits black hole entropy. Additional conditions in L_C could impose "computational constraints," e.g. a "Landauer-type cost," yielding partial differential equations that shape how states on each slice $\tau = const$ evolve.
